# Iron chelation targets lipid metabolism to reduce white matter injury in germinal matrix hemorrhage

**DOI:** 10.1038/s41419-026-08866-z

**Published:** 2026-05-23

**Authors:** Bokun Cheng, Akanksha Mishra, Xusheng Zhang, Zaw Myo Hein, Nourelhoda Gouda, Karen Schaeffer, Mikhail Kislin, Yunping Qiu, Fereshteh Zandkarimi, Irwin J. Kurland, Praveen Ballabh

**Affiliations:** 1 Department of Pediatrics, Albert Einstein College of Medicine, Department of Medicine, Bronx, NY USA; 2 Dominick P. Purpura Department of Neuroscience, Albert Einstein College of Medicine, Bronx, NY USA; 3 Computational Genomics Core, Albert Einstein College of Medicine, Bronx, NY USA; 4 Department of Basic Medical Sciences, College of Medicine, Ajman University, Ajman, UAE; 5 Division of Endocrinology, Department of Medicine, Albert Einstein College of Medicine, Bronx, NY USA; 6 Department of Chemistry, Columbia University, New York, NY 10027 USA

**Keywords:** Acute inflammation, Stroke

## Abstract

Germinal matrix hemorrhage-intraventricular hemorrhage (GMH-IVH) leads to neural cell death and inflammation, resulting in white matter injury in premature infants. Here, we show that IVH-induced iron deposition, ferroptosis, myelination failure, and disruption in lipid and oxylipin metabolism are mitigated by iron chelation using deferoxamine. We also identify the specific lipid and oxylipin metabolites and enzymes dysregulated in IVH, which are rescued by deferoxamine treatment. Our findings reveal that IVH causes iron accumulation, ferroptosis, and apoptosis, and leads to gene enrichment that contributes to cell death and oxidative injury in premature rabbits and human infants. IVH activates phospholipases (PLA2) and lysophosphatidylcholine acyltransferase-3 (LPCAT3), elevating levels of polyunsaturated fatty acids, lysophospholipids, and triacylglycerols, which contribute to neural cell injury. Furthermore, IVH upregulates cyclooxygenase, lipoxygenase, and cytochrome-P450 enzymes, which increases oxylipin production, thereby exacerbating inflammation. Importantly, iron chelation using systemic deferoxamine reduces levels of cPLA2, LPCAT3, and oxylipin-generating enzymes, restoring levels of lysophospholipids, triacylglycerols, and oxylipins in kits with IVH. Consistently, deferoxamine treatment alleviates IVH-induced ferroptosis, inflammation, microglial lipid accumulation, myelination failure, and neurological dysfunction. The study identifies that iron-triggered enzymatic dysregulation of lipid and oxylipin metabolism increases the generation of oxylipins and pro-inflammatory lipid metabolites, contributing to white matter injury in IVH survivors. These effects are mitigated by iron chelation.

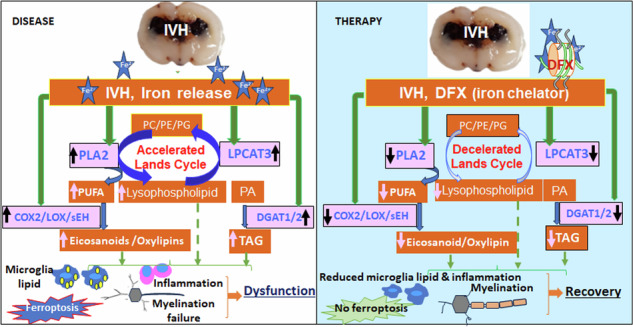

## Introduction

Germinal matrix hemorrhage (GMH)-intraventricular hemorrhage (IVH) remains the most common neurological complication of prematurity, occurring in 20% of all premature infants of ≤30 weeks of gestational age [1]. IVH leads to neural cell death, inflammation, and impaired myelination in the periventricular white matter, resulting in cerebral palsy and cognitive deficits in the survivors [2]. No treatment exists to alleviate the neurological consequences of IVH [3]. In infants with IVH, the ventricular clots release iron through RBC hemolysis, leading to cell death via apoptosis, necrosis, or ferroptosis. Ferroptosis is a process characterized by the iron-catalyzed peroxidation of polyunsaturated fatty acids (PUFA) within membrane phospholipids, leading to the formation of lipid peroxides, cell membrane disruption, and cell death [4]. Hence, we investigated whether IVH could induce ferroptosis and examined the genomic, proteomic, and lipidomic changes that might contribute to ferroptosis, inflammation, and white matter injury (WMI) in newborns affected by IVH. Additionally, we sought to identify previously unknown mechanisms by which iron chelation could alleviate the dysregulation in lipid and oxylipin metabolism, thereby reducing ferroptosis, myelination failure, and neurological dysfunction in survivors of IVH.

Iron homeostasis is critical for health. Excessive iron triggers free radical injury to cells, disrupts mitochondrial function, and affects lipid metabolism. Lipid metabolism plays a crucial role in regulating inflammation and cell injury. PUFA are esterified into phospholipids through the actions of enzymes lysophosphatidylcholine acyltransferase 3 (LPCAT3) and acyl-CoA synthetase long-chain family member 4 (ACSL4). These PUFA-containing phospholipids are the most important oxidizable molecules in the brain, which increase vulnerability to ferroptosis and inflammation [5–8]. Additionally, phospholipase A2 (cPLA2) mediates the hydrolysis of phospholipids releasing PUFA, which can be oxidized into a variety of oxylipins by cyclooxygenases (COX), lipoxygenases (LOX), and cytochrome (CYP) enzymes [9]. These oxylipins play a role in mediating ferroptosis and inflammatory response [10, 11]. The hydrolysis by cPLA2 also produces lyso-phospholipids, which would disrupt the blood-brain barrier and lead to the infiltration of microglia and neutrophils. Lyso-phospholipids are then reacylated by LPCAT3 to reform phospholipids [12]. This cycle of deacylation of phospholipids by PLA2 to produce lyso-phospholipids and free fatty acids (FFAs), followed by acylation by LPCAT3 to reform phospholipids, is known as the Lands cycle. Notably, distinct disruptions in the Lands cycle have been observed in conditions such as Alzheimer’s disease, vascular dementia, Parkinson’s disease, and stroke [13, 14]. However, the changes in lipidomics and oxylipins have not been studied in animal models or human infants with IVH, and no strategies for reversing these changes are currently known.

Microglial accumulation of lipids is associated with pro-inflammatory response and impaired phagocytosis [15]. The microglial droplets are enriched in triacylglycerol, which are synthesized by the enzyme diacylglycerol acyltransferase (DGAT)-1 or DGAT2. Notably, the inhibition of the DGAT2 enzyme reduces microglial lipid droplets, improves microglial phagocytosis, and mitigates neuronal damage in a mouse model of Alzheimer’s disease [16]. Together, the accumulation of microglial TAG contributes to inflammation and brain injury [17, 18].

The neonatal brain has high PUFA concentration, low antioxidant concentration, and a high oxygen consumption rate relative to adults [19]. Indeed, the brains of premature infants are vulnerable to oxidative damage [20], and the occurrence of IVH exacerbates the risk of oxidative injury by accumulating blood and releasing iron. Therefore, iron chelation could benefit premature infants with IVH by counteracting iron-mediated toxicity.

Deferoxamine, an FDA-approved iron chelator, limits cerebral edema and hematoma resorption, favoring the recovery trajectory in patients with intracranial hemorrhage [21, 22]. Iron chelation also offers neuroprotection in animal models of traumatic brain injury, Alzheimer’s disease, and other neurodegenerative diseases [23–25]. Children with Down syndrome and adults with Alzheimer’s disease often exhibit microbleeds, which can potentially improve with iron chelation [26, 27]. Despite this, the mechanistic links between iron chelation, lipid metabolism, and eicosanoid generation that lead to ferroptosis, inflammation, and WMI remain elusive [6, 28]. Based on these considerations, we hypothesized that IVH would induce ferroptosis, WMI, and dysregulated lipid and oxylipin metabolism in premature newborns. Additionally, iron chelation through deferoxamine (DFX) treatment might ameliorate lipid and eicosanoid metabolism, thereby reducing ferroptosis, WMI, and neurological deficits in newborns with IVH. These hypotheses were tested in our extensively published preterm rabbit (E29, Term=32 d) model of glycerol-induced IVH [2, 29, 30], and through analyses of autopsy samples from premature human infants. Our study identified that IVH disrupts enzymes and derivatives of lipid and oxylipin metabolism, and that iron chelation mitigates these metabolic disturbances, thereby minimizing WMI and neurological deficits in IVH survivors.

## Results

### In premature human infants, IVH leads to iron accumulation, ferroptosis, and apoptosis

**Fig. 1:**
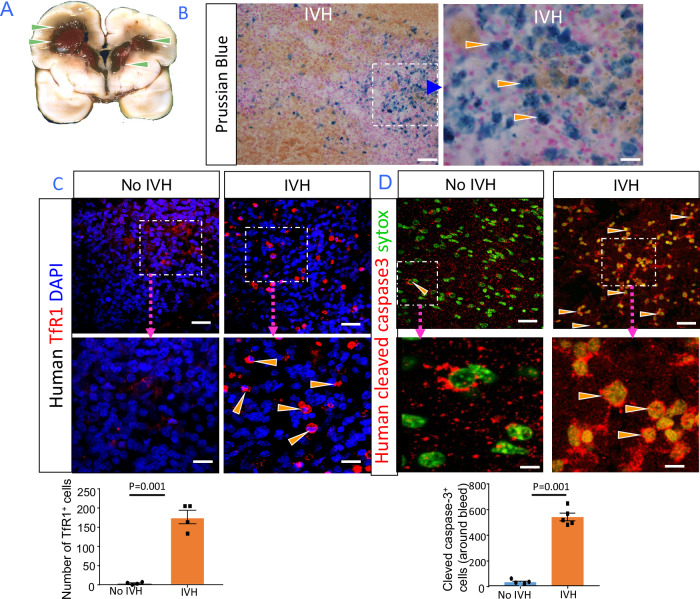
IVH leads to iron deposition, apoptosis, and ferroptosis in premature human infants. **A** Grade III intraventricular hemorrhage (IVH). Coronal slice of the forebrain from a 25-week premature infant showing blood (arrows) in the lateral ventricle. The ventricles are dilated. **B** Representative Prussian blue staining of the coronal section from the periventricular germinal matrix showing iron labeling. The right panel is the high-power view of the boxed area in the left panel. Note the abundance of iron staining (arrowhead). **C** Immunolabeling of the coronal section of a 23-week premature infant with TfR1 (3F3-FMA clone) antibody and DAPI. The lower panel is the high- power view of the boxed area in the upper panel. Note the abundance of ferroptotic (arrowhead) cells in IVH compared to controls without IVH. Bar graphs show mean ± SEM (*n* = 5 each). The student t-test was employed. Scale bars, 50 µm (upper panel) and 20 µm (lower panel). **D** Immunolabeling of the coronal section of a 23-week premature infant with cleaved caspase-3 antibody and sytox (nuclear stain). The lower panel is the high-power view of the boxed area in the upper panel. Note the abundance of apoptotic (arrowhead) cells in infants with IVH in contrast to controls without IVH. Bar graphs are mean ± SEM. The student t-test was employed. Scale bars, 50 µm (upper panel), 15 µm (lower panel).

To determine whether IVH leads to iron accumulation, ferroptosis, and apoptosis in the brains of premature infants with IVH, we evaluated coronal sections from preterm infants of 23-27 weeks of gestational age (autopsy samples, 4-7 postnatal days, Fig. 1A, Table S1). Prussian Blue staining showed an abundance of Fe^3+^ labeling around the ventricle (Fig. 1B). The iron labeling was more extensive in infants with severe IVH (Grade III and IV) relative to small IVH (Grade I and II).

We next evaluated ferroptosis in the periventricular germinal matrix and adjacent white matter of infants with grades III and IV hemorrhage by employing the transferrin receptor 1 (TfR1, clone: 3F3-FMA^+^) antibody, which has been validated for detecting ferroptosis in culture experiments [31]. Given the large size of human brains, we focused on the areas surrounding the hemorrhage in the germinal matrix. We observed an abundance of TfR1^+^ cells, which were significantly higher in infants with IVH compared to controls without IVH (Fig. 1C). However, we were unable to achieve satisfactory staining of human sections with other commercially available antibodies that mark ferroptosis, including 4HNE (4-hydroxynonenal) and anti-monoaldehyde (MDA).

We next evaluated apoptosis in premature infants with IVH compared to controls without IVH. Immunolabeling showed that cleaved caspase-3^+^ cells were abundant in the brain regions surrounding the hemorrhage and relatively scarce in areas distant from the hemorrhage. Accordingly, cleaved-caspase-3^+^ cells were more numerous in infants with IVH compared to controls without IVH (Fig. 1D). When analyses included sex as a covariate (ANCOVA), no differences were observed that could be attributed to the sex of the infants (Table S2). Together, IVH in premature human infants leads to iron overload, contributing to both ferroptosis and apoptosis of neural cells.

### In prematurely delivered rabbit kits, IVH leads to iron deposits, oxidative stress, and ferroptosis

**Fig. 2:**
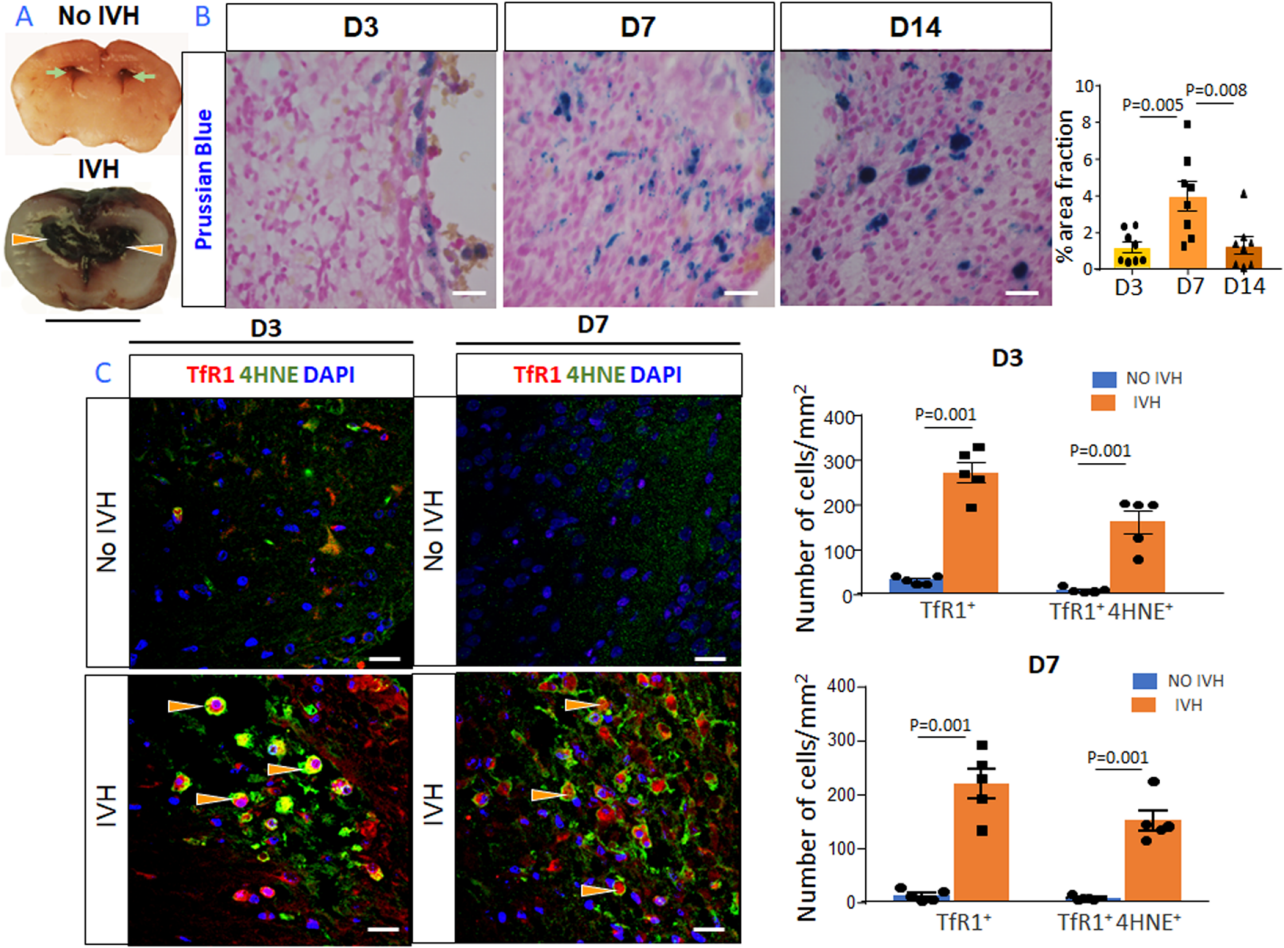
IVH leads to iron deposition, apoptosis, and ferroptosis in premature rabbit kits. **A** Coronal slice through the brain (head of the caudate nucleus level) of E29 rabbit kit showing slit-like ventricles without any hemorrhage (arrows, top) and severe hemorrhage resulting in the fusion of the two ventricles (arrowheads, bottom). (Scale bar, 1 cm.) **B** Cryosections from IVH kits of 3, 7, and 14 days of age were processed for Prussian blue staining, and the area fraction of iron labeling was quantified using MetaMorph software (Molecular Devices). Data are mean ± SEM (*n* = 5 ea.). One-way ANOVA and post-hoc Tukey test were employed. Scale bars, 20 µm. **C** Representative immunofluorescence of coronal sections from D3 and D7 kits labeled with TfR1 (3F3-FMA clone), 4HNE antibody and DAPI. Note the abundance of ferroptosis (TfR1^+^DAPI^+^, arrowhead) in kits with IVH compared to controls without IVH. Bar charts are mean ± SEM. 2-way ANOVA was employed. Scale bars, 20 µm. *P*-values as indicated.

We employed our well-established rabbit model of IVH in E29 kits (term=32 d), in which we induce IVH by IP glycerol and quantify the severity of IVH using head ultrasound at 24 h postnatal age (Fig. 2A) [29, 30]. Prussian blue staining revealed iron labeling predominantly around the lateral ventricles at postnatal day (D) 3, which peaked at D7 and then declined by D14. The quantification demonstrated that the area fraction of Fe3^+^ labeling was more extensive on D7 compared to D3 and diminished at D14 relative to D7 (Fig. 2B).

As iron triggers the generation of reactive oxygen species, we next quantified the lipid mediators of oxidative stress, including isofurans and isoprostanes, by mass spectrometry at D3. We also measured glutathione levels by EnzyChrom GSH/GSSG Assay Kit. We found higher isofuran levels and a lower ratio of reduced glutathione (GSH) to oxidized glutathione (GSSG) in rabbit kits with IVH compared with controls without IVH at D3 (Fig. S1A). Nevertheless, isoprostane and GSH levels were comparable between kits with and without IVH. This suggests that IVH incites oxidative stress, consistent with previous studies [32, 33].

Next, we evaluated ferroptosis in the kits with IVH versus those without IVH at D3 and D7. Transferrin receptor (TfR1, 3F3-FMA clone) antibody has been identified and validated as a ferroptosis-detecting antibody [31]. Hence, we employed TfR1 (3F3-FMA clone) and 4-Hydroxynonenal (4-HNE, free radical adduct) antibodies to identify ferroptotic cells. Double labeling showed remarkable co-localization in the immunoreactivities between these two immunosignals (Fig. 2C). There was an abundance of TfR1^+^4HNE^+^ cells in the periventricular regions of kits with IVH, which were more numerous on the medial wall compared to the lateral wall. Quantification revealed that TfR1^+^4HNE^+^ cells were more abundant in kits with IVH than in controls without IVH at D3 and D7 (Fig. 2C).

To determine the specific neural cell type undergoing ferroptosis, we double-labeled TfR1 with antibodies against Olig2 or Iba1. We noted that TfR1^+^ and 4-HNE^+^ cells co-localized mainly with Iba1^+^ microglia and a few Olig2^+^ cells in the periventricular region (Fig. S1B). Ferroptosis was not observed in GFAP^+^ astrocytes. This suggests that ferroptosis predominantly impacts microglia in IVH. Taken together, IVH results in iron accumulation and ferroptosis in the brain region around the ventricles.

### IVH results in the enrichment of genes that trigger ferroptosis and inflammation in scRNA-seq

To evaluate the effect of IVH on the transcriptomic profile of the principal types of periventricular neural cells, we carried out ScRNAseq of the dissected outer wall of the lateral ventricle (remnant of medial and lateral ganglionic eminences) from kits with and without IVH at 72 h of age.

**Fig. 3:**
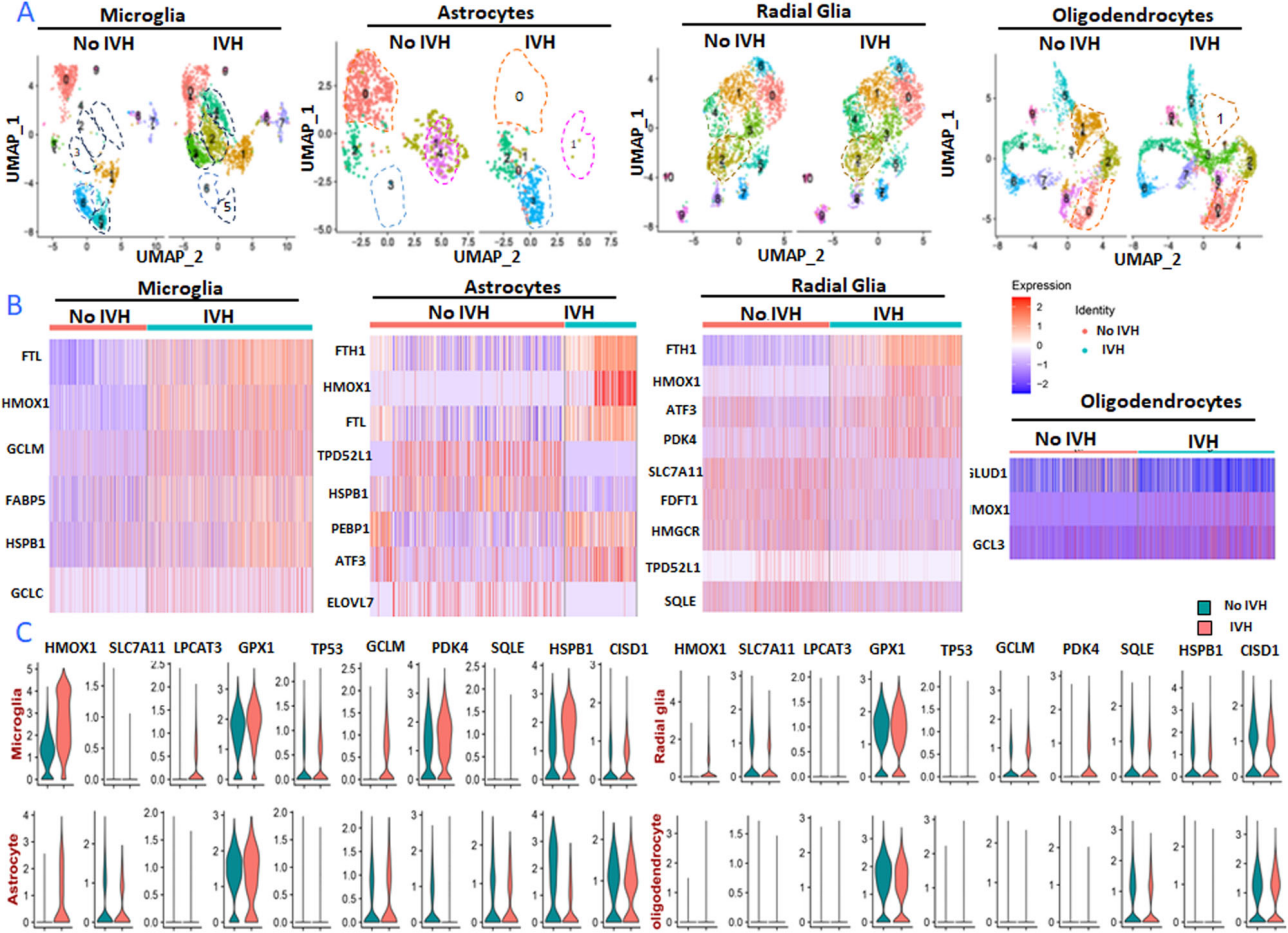
Single-cell transcriptome-wide profiling of microglia, astrocytes, radial glia, and oligodendrocytes from the lateral periventricular zone (medial and lateral ganglionic eminences) of kits with and without IVH. **A** UMAP (uniform manifold approximation) visualization of microglia (left), astrocyte (middle), radial glia (middle) and oligodendrocytes (right) showing their unbiased clustering into multiple subclusters (represented by distinct colors) in kits with and without IVH, as indicated. Note subclusters 1 (KIF20B, KIF11), 2 (NME2, CD63), 3 (LOC100339314, SPP1), and 4 (RPL31, TMSB10) in ‘IVH’ and subclusters 5 (TTR, P2RY12), and 6 (TTR, SPP1) in ‘no IVH’ show greater abundance among microglial cells. Subcluster 0 (HMGCS1, TSPAN7), 1 (WFIKKN2, ND1), and 4 (MSX1, CLDN3, WFIKKN2) in ‘no IVH’ and subcluster 3 (LOC100342544, RPS21) in the IVH category exhibit greater abundance among astrocytes. Subcluster 2 (TTR, FGFBP3) in ‘no IVH’ and subcluster 4 (LOC100342544, FTH) in the IVH category are dominant among radial glia clusters. Within oligodendrocyte clusters, subcluster 1 (TTR, LOC103348693) and 2 (SCRG1, B2M) in no IVH and subcluster 0 (BTG2, RPL26) in IVH kits dominate. **B** Heat Map for selected genes in microglia, astrocytes, radial glia, and oligodendrocytes are differentially expressed in kits with IVH compared to kits without IVH. The color represents the percentage expression of the genes. Annotations on the y-axis describe the name of the gene. The red, white, and blue colors represent high, average, and lower expression of a particular gene, respectively. **C** Violin plots of canonical genes for ferroptosis are differentially expressed in the microglia, astrocytes, radial glia, and oligodendrocytes of the lateral periventricular zone of kits with and without IVH. HMOX1, TP53, LPCAT3, PDK4, and HSPB1 genes were enriched in microglia. GCLM, PDK4, and HSPB1 genes accumulated whereas SLC7A11 gene was depleted in radial glia. Among astrocytes, expression of HMOX1 and GPX1 was elevated, while HSPB1, SQLE, and PDK4 were reduced. In oligodendrocytes, GPX1 and SQLE were depleted.

IVH altered the expression of several genes in microglia, astrocytes, radial glia, and oligodendrocytes. The distinct subclusters of genes in each of these cells were identified in kits with IVH vs. kits without IVH, as depicted in Fig. 3A. In the microglia, IVH upregulated 183 genes and downregulated 225 genes. IVH enriched microglia with genes triggering ferroptosis, including *HMOX1, FTL, GCLM, FABP5, HSPB1*, and *GCLC* (Fig. 3B). In astrocytes, 241 genes accumulated, and 212 genes reduced in kits with IVH relative to controls. The key genes regulating ferroptosis, *HMOX1, FTH1, FTL, PEPB1*, and *ATF3*, were enriched, whereas *TPD52L1, HSPB1*, and *ELOVL7* were depleted in astrocytes of kits with IVH (Fig. 3B). Among radial glia, 168 genes were upregulated, and 329 were downregulated in kits with IVH compared to controls. The elevation of *HMOX1, FTH1, ATF3, and PDK4* and reduction of *SLC7A11, FDFT1, HMGCR, SQLE* genes in the radial glia would increase ferroptosis sensitivity in kits with IVH.

Among genes regulating iron metabolism, heme oxygenase (*HMOX1)* and ferritin heavy and light chain *(FHL and FTL)* were enriched in astrocytes, microglia, oligodendrocytes, and proliferating progenitors of kits with IVH (Fig. 3B, C). *LPCAT3 was* abundant in both microglia and endothelia kits with IVH, but *ASCL3* and *ASCL4* were comparable between the two groups (Fig. 3B, C). IVH elevated *TP53* and its transcriptional target *SAT1* in microglia, promoting ferroptosis. IVH enriched *PKD4, FABP5,* *FABP7, and PEPB1* genes in the microglia and ependyma, supporting ferroptosis (Fig. 3C) [34]. Glutamate-cysteine ligase (*GCLC*) was elevated in microglia and proliferating cells of kits with IVH, protecting cells from ferroptosis.

Among genes limiting lipid peroxide generation, *SLC7A11* was reduced in the radial glia and astrocytes, CISD1 in ependyma, radial glia, and oligodendrocytes, and *GPX1* gene in oligodendrocytes and proliferative cells of kits with IVH. These changes would contribute to oxidative stress and ferroptosis [35]. Reducing the *HMGCR* in oligodendrocytes, proliferating progenitors, and radial glia and depletion in the *SQLE* gene in oligodendrocytes, radial glia, and proliferating cells would promote ferroptosis. *FSP1, DHFR, GCH1, and NRF2* genes were comparable in the two groups. Eicosanoid regulating genes, including LOX genes (*ALOX-5, -E3, -12B*, and *-15*), *COX* (*PTGS2* and *PTGS1*), *CYP27A1, CYP2J1*, and *EPHX2*, were comparable between kits with and without IVH. Together, IVH induces enrichment of genes favoring ferroptosis, iron metabolism, and oxidative stress mainly in microglia.

### DFX reduces periventricular iron deposition, ferroptosis, apoptosis, and inflammation

**Fig. 4:**
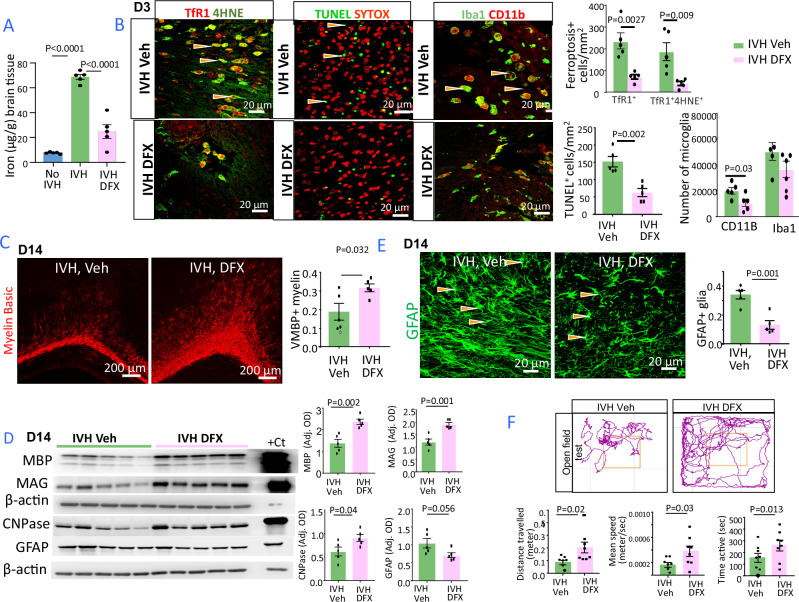
DFX treatment reduces iron level, ferroptosis, apoptosis, inflammation, and gliosis as well as promotes myelination. **A** Iron level was measured by ICP-MS. Bar graphs are mean ± SEM (n = 5 each). The one-way ANOVA and Tukey’s post-hoc test were employed. Iron levels were elevated in kits with IVH relative to kits without IVH. DFX treatment reduced iron levels in kits with IVH. **B** Representative immunofluorescence of coronal sections from DFX and vehicle-treated kits with IVH at D3. The sections are labeled with TfR1 and 4-HNE antibodies (left panel), TUNEL and Sytox (middle panel), and Iba1 and CD11B antibodies. Note the reduced abundance of TfR1^+^4-HNE^+^, TUNEL^+^, and CD11B^+^ cells (arrowhead) in DFX-treated kits (lower panel) relative to controls (upper panel). Bar graphs are mean ± SEM (*n* = 5 each). The Student t-test was employed. Scale bars, 20 µm. **C** Representative immunofluorescence of MBP in the corona radiata of DFX and vehicle-treated kits with IVH at D14. Note the greater abundance of MBP+ immunoreactivity in DFX-treated kits relative to controls. The bar chart shows the mean ± SEM (*n* = 5 per group). The volume fraction of MBP was higher in DFX-treated kits compared to controls. **D** Western blot analyses for MBP (13-23 kD), MAG (69kD), CNPase (46kD), and GFAP (46kD) were performed in homogenates from DFX and vehicle-treated kits with IVH. The adult rat brain was used as a positive control. The bar graph shows mean ± s.e.m (*n* = 5 ea). The student t-test was used. Values were normalized to β-actin levels. Scale bar and *P*-values as indicated. **E** A typical immunofluorescence of GFAP in the corona radiata of DFX- and vehicle-treated kits with IVH at D14. Note a reduced abundance of hypertrophied astrocytes (arrowhead) and arborization in the forebrain of DFX treated kits relative to vehicle controls. The bar chart indicates mean ± SEM (*n* = 5 in each group). The volume fraction of GFAP^+^ signals was less in DFX-treated kits than in controls. **F** DFX treatment enhanced motor function. The upper panels show representative tract plots of distance traveled by the vehicle and DFX-treated kits with IVH. Bart charts are mean ± s.e.m (*n* = 8 to 10 in each group). The student t-test was used. An open-field test was conducted using Any Maze on day 14. DFX-treated kits traveled longer distances, displayed more speed, and were more active in the center and whole arena than vehicle-treated kits.

To reduce iron-induced ferroptosis and oxidative injury in the brains of rabbit kits with IVH [32], we treated them with IM deferoxamine **(**DFX) or saline from D1 through D7 (DFX, 50 mg/kg twice daily). Prussian blue labeling demonstrated a significant reduction in the percentage area fraction of iron labeling in the periventricular region of DFX-treated kits compared to vehicle controls at D7 (Fig. S2), a difference not significant at D3. Iron assay by inductively coupled plasma mass spectrometry (ICP-MS) confirmed that the iron level was ten-fold higher in the brain tissues of kits with IVH compared to kits without IVH at D3, and it decreased significantly after 3 days of DFX treatment (Fig. 4A).

Next, we evaluated ferroptosis in DFX- vs. vehicle-treated kits by double-labeling coronal brain sections with TfR1 and 4HNE antibodies. We found that TfR1^+^ and TfR1^+^4HNE^+^ cells were reduced in the periventricular regions of DFX-treated kits compared to vehicle controls with IVH at D3 (Fig. 4B). We then assessed apoptosis in DFX- vs. vehicle-treated kits with IVH by performing TUNEL (Fluorescent in situ detection of DNA fragmentation) labeling of coronal sections. We found that TUNEL^+^ cells were significantly reduced in the periventricular region of the DFX-treated kits relative to controls at D3 (Fig. 4B). Including sex as a covariate revealed no differences attributable to infant sex (Table S2).

To assess the effect of DFX on inflammation, we compared microglia infiltration in the periventricular region of the cerebral cortex. CD11B^+^ activated microglia were reduced in DFX- compared to saline-treated kits with IVH at D3 (Fig. 4B), but Iba1^+^ microglia were comparable between the two groups. Together, DFX treatment showed efficacy by reducing iron levels, ferroptosis, apoptosis, and inflammation in kits with IVH.

### DFX reduces gliosis and improves myelination as well as neurological function

Our previous studies have shown that IVH leads to inflammation, myelination failure, astrogliosis, and neurological dysfunction in rabbit kits [2, 30]. Since DFX treatment reduced ferroptosis and attenuated inflammation, we postulated that DFX treatment would restore myelination and reduce gliosis in preterm kits with IVH. The stereological quantification of immunolabeled sections with myelin basic protein (MBP)-specific antibody showed that the volume fraction (myelin load) of MBP in the corpus callosum and corona radiata was increased in DFX-treated kits with IVH compared with vehicle controls (Fig. 4C). Consistent with immunohistochemistry, Western blot analyses showed that DFX treatment significantly enhanced MBP, myelin-associated glycoprotein (MAG), and CNPase expression in kits with IVH (Fig. 4D).

We next assessed the effect of DFX treatment on astrogliosis in kits with IVH. We quantified the volume fraction of GFAP-labeled astrocytes in coronal sections by unbiased stereology. We found that the volume fraction of GFAP^+^ astrocyte cell bodies and glial fibers was reduced in DFX-treated kits relative to vehicle controls (Fig. 4E). Consistent with these findings, western blot analyses showed that GFAP levels showed a trend toward decline in DFX-treated kits relative to vehicle controls (Fig. 4D). Together, the data suggest that DFX treatment promotes myelination and reduces gliosis.

The prematurely born infants with IVH often suffer from motor deficits [36]; and the rabbit kits with IVH display impaired motor function in the Open Field Test [30]. Hence, we assessed the impact of DFX treatment on motor function in kits with IVH using open-field tests at D14. We demonstrated that DFX-treated kits traveled a greater distance in the arena relative to controls (Fig. 4F). DFX treatment increased speed during locomotion (Fig. 4F). Moreover, the percentage of time spent active in the arena was higher in DFX-treated kits compared to controls. When sex was included as a covariate, no differences were observed that could be attributed to infant sex (Table S2). In conclusion, iron chelation with DFX treatment improves myelination and motor function of kits with IVH.

### IVH disrupts the cerebral lipid profile, and DFX rescues

As lipid metabolites play key roles in inducing an inflammatory response in traumatic brain injury [37], we postulated that IVH would disrupt the lipid profile and that treatment with DFX could restore it. To this end, we performed lipidomic profiling for (a) kits with versus without IVH at 24 and 72 h of age and (b) DFX- versus saline-treated kits with IVH at 72 h.

**Fig. 5:**
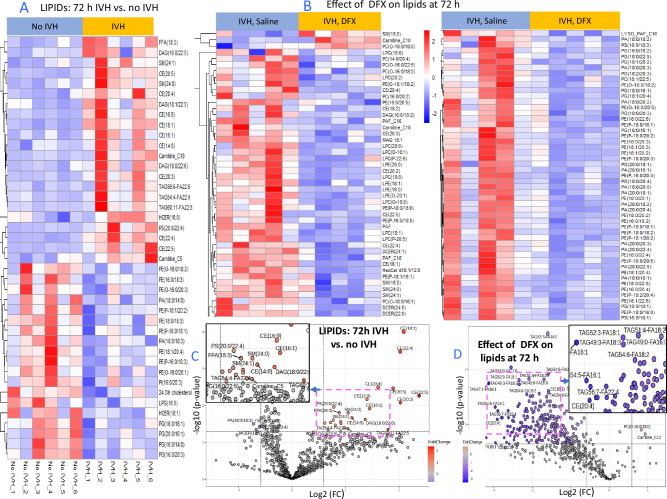
IVH disrupts phospholipid metabolism and increases many cholesterol esters (CEs), free fatty acid, triacyl glycerol (TAG) as well as carnitine levels, whereas DFX treatment rescues. **A** The heatmap depicts the effect of IVH on the lipid profile at 72 h of age. The X-axis shows IVH vs. no IVH at 72 h age. Annotations on the Y-axis show the type of lipid. The red, white and blue colors represent high, average and lower expression of lipids, respectively. **B** The heatmap displays the effect of DFX on the lipid profile at 72 h of age. Annotations on the X-axis show IVH with saline treatment vs. IVH with DFX treatment at 72 h age. Annotations on the Y-axis indicate the kind of lipid. The red, white and blue colors represent high, average and lower expression of lipids, respectively. **C** Volcano plots depict the impact of IVH on lipid profile at 72 h. Log2 (FC) values are plotted on the X-axis and -log10 (*p*-value) on the Y-axis. FFA, TAGs and CEs are elevated in IVH at 72 h age. The inset shows the magnified view of the boxed area in the image. **D** Volcano plot depicts the effect of DFX on lipid profile. The X-axis shows log2 (FC) value, and the Y-axis indicates -log10 (*p*-value). Note that many triacylglycerols (TAG) are downregulated upon DFX treatment. The inset shows the magnified view of the boxed area in the image.

PLS-DA analyses identified analytes of interest in kits with IVH versus those without IVH at 24 and 72 h after hemorrhage induction. We observed that many membrane phospholipid species, phosphatidylethanolamine (PE 16:0/18:1, 18:0/18:3,18:0/20:4, others) and phosphatidylglycerol (PG 16:0/14:0) were reduced in kits with IVH at 72 h (Fig. 5A, Figs. S3A, S4A–C). Likewise, some phosphatidylcholine types (PC14:0/14:0,14:0/18:3, 14:0/20:3, others) were depleted in kits with IVH at 72 h of age, while other PC types (16:0/14:0, 16:0/20:1,16:0/20:2, 20:0/18:1) were diminished in kits with IVH at both 24 and 72 h of age. In contrast, several species of lysophospholipids, including LPC, LPG, and LPI, were elevated in kits with IVH at both 24 and 72 h of age. The elevation in PE and a reduction in the PC levels would promote ferroptosis in kits with IVH [8, 38].

A decline in phospholipids that contain one saturated and one unsaturated side chain (C20) indicates the release of C20 polyunsaturated fatty acids (PUFA), such as arachidonic acid, for the synthesis of eicosanoids. We observed an accumulation of cholesterol esters in the brains of kits with IVH at both 24 and 72 h (Fig. 5A,–C, Figs. S3A, S3B). About 255 types of triacylglycerol (TAG) were significantly elevated in the brain of kits with IVH at 24 h age, with five classes of TAG remaining high even at 72 h (Fig. 5A,–C, Figs. S4A, 4B). This suggests that TAG generation was more pronounced at 24 h than at 72 h. Elevated levels of acylcarnitines—including C8, C10, C16, and C18—were observed in kits with IVH at 24 h, while C5 and C18 carnitine remained elevated at 72 h. This reflects the accumulation of fatty acids that were not effectively oxidized during both time points. Overall, the elevation of lysophospholipids and free fatty acids induced by IVH indicates the activation of phospholipase enzymes, leading to an accelerated Lands cycle. Furthermore, the increase in TAG suggests heightened activity of diacylglycerol acyltransferase (DGAT) enzymes. These changes would promote inflammation, gliosis, and tissue injury [39].

**Fig. 6:**
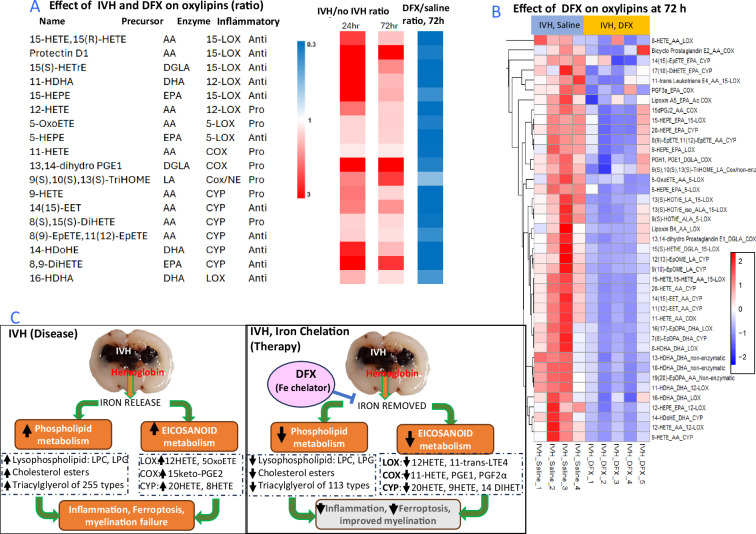
DFX reduces IVH-induced elevation in oxylipins. **A** The heatmap shows a) the ratio of eicosanoids in kits with IVH and without IVH (IVH/no IVH) at 24 and 72 h age as well as **B** the ratio of eicosanoids in DFX- and saline-treated (IVH, DFX/ IVH, saline) kits with IVH. Annotation on the X-axis depicts the name of eicosanoids, precursors, the enzymes involved in eicosanoid generation, anti- or proinflammatory type, and the ratio of their concentrations (IVH/no IVH and DFX/Saline treated IVH kits) as depicted. Note that all eicosanoids shown are elevated in kits with IVH at 24 and 72 h of age, and DFX therapy reduces their levels. The red and blue colors represent elevated and reduced ratio of eicosanoids, respectively. **B** The heatmap shows the effect of DFX on the levels of oxylipins/eicosanoids at 72 h of age. Note that most of the eicosanoids are reduced with DFX treatment. Annotation on the X-axis shows DFX vs. saline at 72 h age. Annotations on the Y-axis depict the type of eicosanoid. The red, white and blue colors represent high, average and lower expression of eicosanoids, respectively. **C** Schematic depicts that IVH accelerates the phospholipid and eicosanoid metabolism resulting in production of toxic lipid metabolites and inflammatory eicosanoids (oxylipins). This would induce inflammation, ferroptosis and myelination failure. Notably, iron chelation with deferoxamine (DFX) rescues these changes.

Next, we evaluated the impact of DFX treatment on lipid profiles in kits with IVH (Fig. 5B–D, Figs. S3B and S5A, S5B). Several cholesterol esters and dihydroceramide species were reduced in DFX- compared to saline-treated kits with IVH at 72 h. In addition, LPC and LPE were depleted in DFX-treated kits with IVH, which might reduce lysophosphatidic acid generation [40]. DFX treatment also reduced some phospholipids (PA, PE, and PG), platelet-activating factors (PAF), and lyso-PAF (C16). Additionally, DFX treatment depleted 113 TAG types in the brains of kits with IVH, suggesting DFX-induced downregulation in DGAT1/2 enzymes (Fig. S5A, S5B**)** [41, 42]. DFX increased C10 carnitine levels while decreasing C18 carnitine, indicating enhanced C18 fatty acid oxidation and improved mitochondrial function. Hence, the reduction in phospholipids, lysophospholipids, PAF, and TAG triggered by DFX would lower sensitivity to ferroptosis, inflammation, and injury (Fig. 6C) [39, 43].

Since DFX treatment reduced the elevated TAG levels induced by IVH, we hypothesized that DFX therapy would decrease TAG expression in microglia. To investigate this, we double-labeled brain sections using BODIPY (for lipid droplet staining) and Iba1 (a microglial marker) antibodies (Fig. S5C). Our analysis revealed that the number of both Iba1+ and Iba1+BODIPY+ cells was significantly lower in the periventricular brain regions of DFX-treated kits compared to the control group on Day 3. This result confirms that DFX treatment effectively depleted TAG in the microglia of kits with IVH.

### IVH escalates, and DFX reduces oxylipin production

IVH increased the generation of free fatty acids (precursors of oxylipins), induced cell death, and triggered inflammation. These effects were alleviated by DFX treatment. Therefore, we evaluated oxylipins in two contexts: (i) kits with vs. without IVH at 24 and 72 h of age, and (ii) IVH kits treated with DFX vs. controls at 72 h of age (Fig. 6, Fig. S6A, B). We quantified metabolites of the COX, LOX, and CYP pathways. In LOX pathway, we found that 12-HETE and 5-OxoETE, both pro-inflammatory molecules, were elevated by 2-3 times at 24 and 72 h. Among anti-inflammatory LOX oxylipins, lipoxin B4, and protectin D1 levels were enriched at both 24 and 72 h in kits with IVH. In the COX pathway, 15-keto Prostaglandin E2 was elevated in kits with IVH by 4 times at 24 h and by 5 times at 72 h, whereas 11-HETE was raised by two-fold at 24 h of age. Next, we evaluated the pro-inflammatory molecules of the CYP pathway. 20-HETE, a powerful pro-inflammatory eicosanoid, was elevated at both 24 (104-fold) and 72 h (30-fold). Additionally, 8-HETE, 9-HETE, and 14(15)-DiHET accumulated in kits with IVH at both 24 and 72 h. The key anti-inflammatory metabolites of the CYP pathway, including 14(15)-EET, 15-OxoETE, 14-HDoHE,13(14)-DiHDPA, 17(18)-DiHETE, 11(12)-EET, and 9 (10)-DiHOME were also significantly elevated in kits with IVH at both 24 and 72 h.

Next, we evaluated the effect of DFX treatment on oxylipin/eicosanoid generation at 72 h in kits with IVH (Fig. 6A, B). The pro-inflammatory metabolites of LOX pathway, including 12-HETE, 8-iso Prostaglandin F3α, 11-trans-leukotreine E4, and leukotriene E4, were reduced by 3-6-fold in DFX-treated kits with IVH relative to controls, respectively. However, DFX treatment also depleted several anti-inflammatory metabolites, including lipoxin A5, 5-HEPE, 9-HEPE, 12-HEPE, 15-HEPE, 15-HETE, 8-HDHA, 16-HDHA, 15dPGJ2, and 15(S)-HORTrE, compared to controls. We next assessed the impact of DFX treatment on the eicosanoids of the COX pathway (Fig. 6A, B). 11-HETE, 13,14-dihydro prostaglandin (PG) E1, PGF2a, and 11β-13,14-dihydro-15-keto PGF2α were reduced in

DFX-treated kits relative to controls at 72 h of age. Among the metabolites of the CYP pathway, DFX treatment reduced two inflammatory eicosanoids, 20-HETE and 9-HETE, by 6-fold. The anti-inflammatory oxylipins of CYP pathway, including 14-HDoHE, 8(9)-EpETE, 14(15)-EpETE, 17(18)-DiHETE, 20 HEPE, 11(12)-EET and 14(15)-EET, were also diminished on DFX treatment. Although DFX treatment decreased both pro- and anti-inflammatory oxylipins, the reduction of the major pro-inflammatory molecules, including 20-HETE, 12-HETE, 8-iso-PGF2α, 11-trans-leukotriene E4, and leukotriene E4, would contribute to neurological recovery in kits with IVH.

### IVH activates enzymes regulating iron and lipid metabolism, while DFX inhibits these enzymes

To identify the underlying enzymatic changes that led to the generation of toxic lipid metabolites and oxylipins, we assayed enzymes regulating iron and phospholipid metabolism in (a) kits with vs. without IVH, and (b) IVH kits treated with DFX vs. vehicle at postnatal days (D) 3 and 7.

**Fig. 7:**
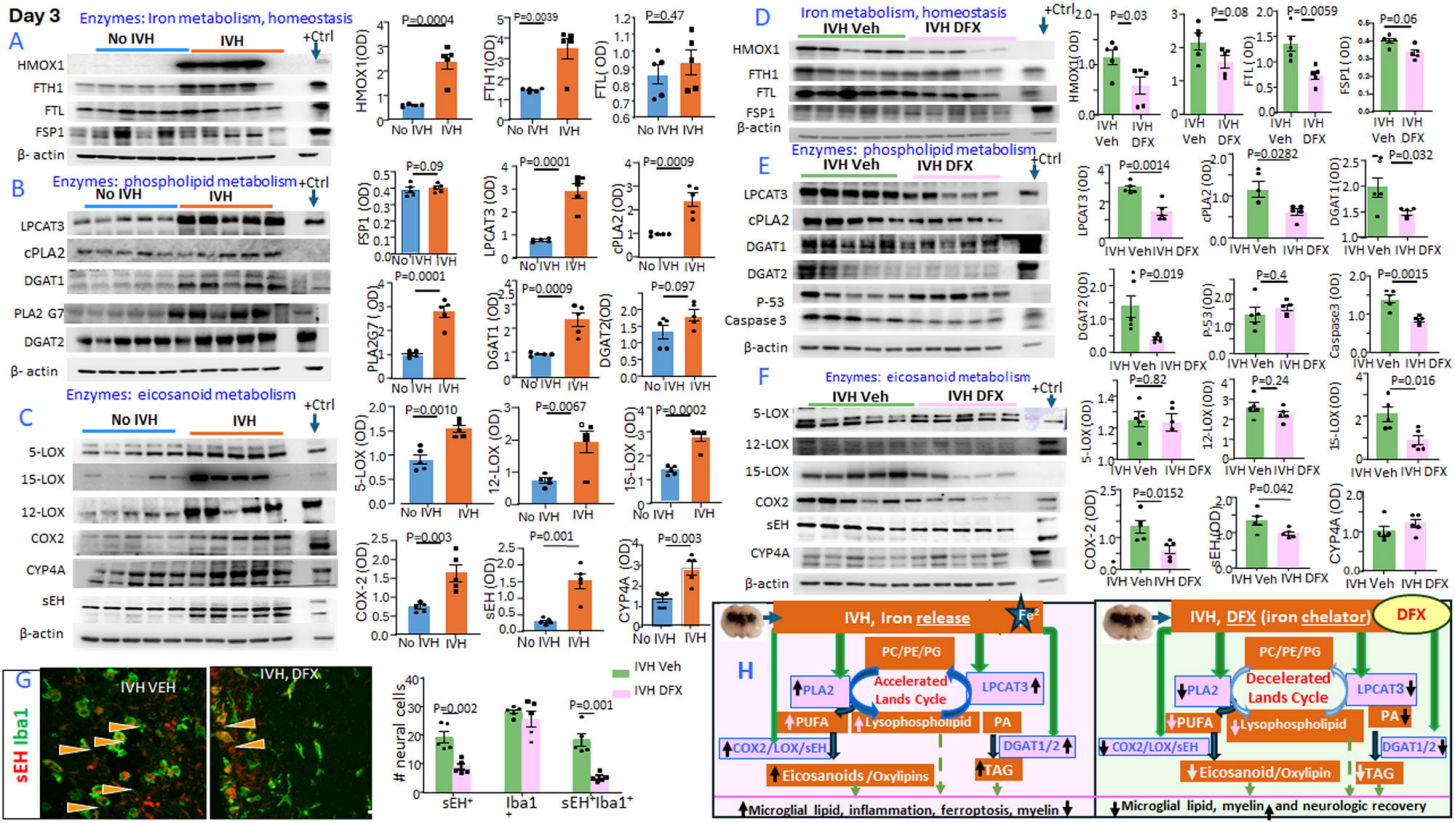
IVH activates key enzymes regulating iron, phospholipid, oxylipin enzymes and DFX treatment rescues. **A, C** Representative Western blot analyses on homogenates from kits with IVH vs. IVH for molecules regulating (i) iron metabolism, including HMOX1 (28-33kD), FTH1 (21kD), FLT1 (19kD) and FSP1 (40 kD), (ii) phospholipid metabolism, including LPCAT3 (52kD), cPLA2 (98 kD), DGAT1 (55 Kd), and PLA2G7 (29kD),and (iii) eicosanoid metabolism, including LOX5 (78kD), 12-LOX (76kD), 15-LOX (75kD), COX2 (70kD), Cyp4A (50-60 Kd, and sEH (62kD. Student t-test was employed Values were normalized to β-actin levels. The bar graph shows mean ± s.e.m (*n* = 5 ea). **D–F** Representative Western blot analyses on homogenates from IVH kits with treated with DFX or control for molecules regulating (i) iron metabolism, including HMOX1 (28-33kD), FTH1 (21kD), FLT1 (19kD), and FSP1 (40 kD), (ii) phospholipid metabolism, including LPCAT3 (52kD), cPLA2 (98 kD), DGAT1 (55 Kd), and PLA2G7 (29kD), and (iii) eicosanoid metabolism, including LOX5 (78kD), 12-LOX (76kD), 15-LOX (75kD), COX2 (70kD), Cyp4A (50-60 Kd, and sEH (62kD. Student t-test was employed Values were normalized to β-actin levels. The bar graph shows mean ± s.e.m (*n* = 5 ea). **G** Representative immunofluorescence of coronal sections from DFX- and vehicle-treated kits with IVH at D3. The sections were labeled with Iba1 and sEH-specific antibodies. Note that sEH (arrowhead) is abundantly expressed in Iba1^+^ microglia in vehicle-treated kits and scarcely in DFX-treated kits. Bar charts are mean ± SEM. Student t-test was employed. Scale bars, 20 µm. *P*-values as indicated. **H** The schematic shows IVH activates HMOX1, breaking down hemoglobin and releasing iron. IVH and released iron accelerate the Lands cycle by activating cPLA2 and LPCAT3 to release free fatty acid (FFA) and lyso-phospholipids. FFA is oxidized to oxylipins by COX, LOX, and CYP enzymes. In addition, IVH activates DGAT1 synthesizing TAGs. The DFX treatment mitigates each of these effects.

Western blot analyses showed that HMOX1 and FTH1 protein levels were increased in kits with IVH at both D3 and D7 (Fig. 7A, Fig. S7A). DMT1 expression was also reduced at D7 relative to the controls, but not at D3. The recently identified ferroptosis suppressor protein (FSP1) was similar between kits with and without IVH at D3. This indicates that IVH facilitates iron release by increasing HMOX1 levels while simultaneously reducing free iron by boosting FTH1 expression.

We then evaluated the enzymes of phospholipid (Lands cycle) metabolism. LPCAT3, cPLA2, and PLA2G7 were elevated at D3, but this increase was not seen at D7 (Fig. 7B, Fig. S7B**)**. The ASCL4 expression was comparable between kits with and without IVH at D3 and D7. The protein levels of P53 were elevated in kits with IVH at both D3 and D7 (Figs. S7B, S8A). DGAT1, which synthesizes TAG, was also elevated in kits with IVH at D3, but not DGAT2. An elevation in LPCAT3 and cPLA2, accompanied by an increase in lysophospholipids and free fatty acids, confirms the acceleration of the Lands cycle in IVH.

Among protectors of ferroptosis, SLC7A11, GPX1, GPX4, NRF2, and KEAP1 protein levels were comparable between kits with and without IVH at both D3 and D7 (Figs. S7C and S8A**)**. MBOAT1 and MBOAT2 are ferroptosis suppressors; MBOAT1 was elevated in IVH at D3, whereas MBOAT2 was not (Fig. S8A). RT-qPCR using TaqMan probes revealed that *HMOX1* expression was higher in kits with IVH relative to controls (Fig. S8B). However, mRNA expressions of *SLC7A11, NEF2L2, LPCAT3, GPX1*, and *DMT1* were comparable between kits with and without IVH. Together, IVH accelerates iron metabolism, the Lands cycle, and TAG production.

As DFX treatment reduced iron levels, decreased cell death, and promoted myelination, we hypothesized that DFX treatment would affect the enzymes involved in regulating iron, lipid, and oxylipin/eicosanoid metabolism. Western blot analyses revealed that HMOX1 expression was significantly reduced in DFX-treated kits compared to saline controls at D3 (Fig. 7B), but not at D7. DFX treatment elevated DMT1 levels at D7 (Fig. S7A), a difference that was not seen at D3. Consistent with protein expression, HMOX1 mRNA expression was reduced in DFX-treated kits compared to controls at D3 (Fig. S8D**)**.

Next, we quantified the pivotal enzymes that regulate lipid metabolism by Western blot analyses. LPCAT3 and cPLA2 expressions were reduced in DFX-treated kits relative to controls at D3 (Fig. 7E), but no such difference was noted at D7. DFX treatment also reduced DGAT1 and DGAT2 in kits with IVH at D3. ASCL4 and PLA2G7 levels were comparable between the two groups. The DFX-triggered downregulation of LPCAT3 and cPLA2 confirmed that iron chelation decelerates the Lands cycle in kits with IVH.

DFX treatment did not affect proteins that prevent ferroptosis, such as SLC7A11, GPX1, GPX4, FSP1, NRF2, MBOA1, and MBOAT2 at days 3 and 7 (Figs. 7D, S8C, S9C**)**. Accordingly, *SLC7A11, NRF2 (NEF2L2), GPX1, LPCAT3, DMT1* mRNA levels were similar in DFX and vehicle-treated kits (Fig. S8D**)**. DFX therapy also elevated P53 at D7 (Fig. S9B), not at D3. Notably, DFX treatment reduced cleaved-caspase-3 expression in kits with IVH at both D3 and D7 (Fig. 7E, Fig. S9B), thereby reducing apoptosis in IVH survivors. Analysis of the effects of IVH and DFX treatment, with sex included as a covariate, revealed no significant differences in enzyme levels across any gender (Tables S2 and S3).

Together, IVH (i) releases iron by upregulating HMOX1, (ii) accelerates the Lands cycle by activating cPLA2 and LPCAT3, (iii) raises TAG synthesis by stimulating DGAT1, and (iv) causes apoptosis by activating caspase 3. Each of these processes is reversed by DFX treatment, decelerating the Lands cycle, reducing TAG synthesis, and blocking apoptosis in IVH survivors.

### DFX treatment reduces IVH-induced elevation in oxylipin-regulating enzymes, specifically lowering microglial soluble epoxy hydroxylase enzyme

To identify the mechanisms by which IVH escalates oxylipin production and DFX treatment reverses this process, we quantified the enzymes of the LOX, COX, and CYP pathways. We evaluated kits with and without IVH, as well as DFX-treated kits versus vehicle-treated kits with IVH.

Western blot analyses revealed that 5-, 12-, and 15-LOX, as well as COX-2 levels, were increased in rabbits with IVH compared to controls without IVH at D3 (Fig. 7C). The 5-LOX and 12-LOX expressions remained higher in kits with IVH relative to controls at D7 (Fig. S7B). In addition, IVH elevated the soluble epoxide hydroxylase (sEH), and CYP4A (Fig. 7C) levels at D3.

Next, we quantified the impact of DFX treatment on these enzymes. DFX treatment reduced 15-LOX in kits with IVH at D3 (Fig. 7F), but did not affect 5-LOX and 12-LOX levels. These three lipoxygenases (5-, 12-, 15-LOX) were similar in DFX- and vehicle-treated kits at D7. Additionally, COX-2 and sEH expressions were reduced in DFX-treated kits relative to controls at D3, but no such impact was observed for CYP4A. Together, IVH activates several enzymes of the COX, LOX, and CYP pathways, which are rescued by DFX treatment.

Since the inhibition of the sEH enzyme reduces microglial-mediated inflammation in Alzheimer’s disease [44], we evaluated sEH in the microglia of DFX- and vehicle-treated kits with IVH. Double labeling with Iba1 and sEH antibodies revealed that sEH was predominantly expressed in the microglial cytoplasm of kits with IVH. In addition, sEH^+^ and Iba1^+^sEH^+^ cells were reduced in DFX- compared to vehicle-treated controls (Fig. 7G). Together, DFX treatment reduces sEH protein levels and sEH^+^ microglial density, suggesting a novel mechanism by which DFX reduces inflammation in kits with IVH. The effects of IVH and DFX treatment were evaluated with sex included as a covariate, and no significant differences in enzyme levels were detected (Tables S2 and S3)

In summary, IVH accelerates the Lands cycle by activating cPLA2 and LPCAT3, raises TAG synthesis by stimulating DGAT1, and elevated oxylipin levels; the DFX treatment mitigates these effects (Fig. 7H).

### In human infants, IVH activates enzymes for iron, lipid, and oxylipin/eicosanoid metabolism, specifically in microglia

**Fig. 8:**
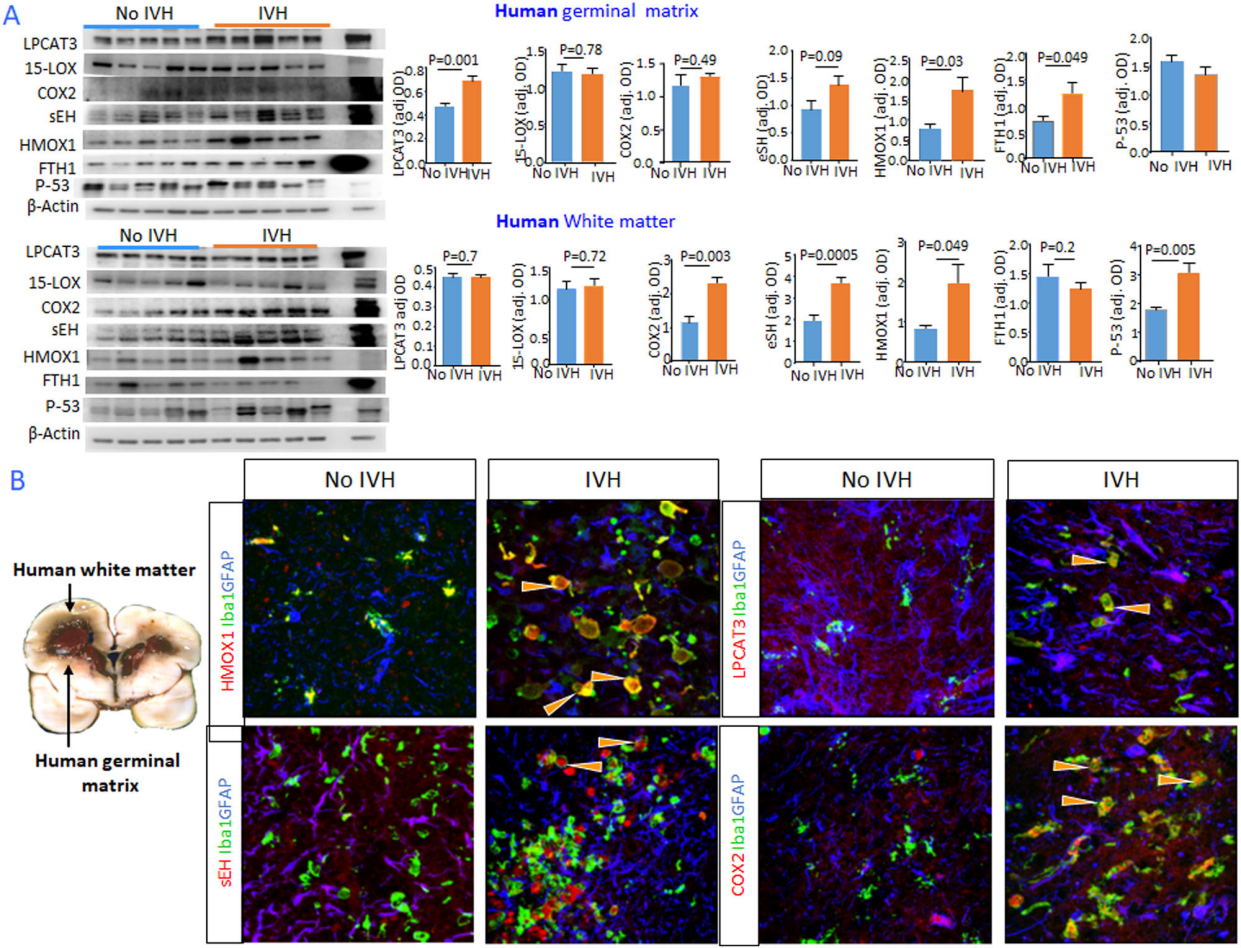
IVH increases the expression of HMOX1, LPCAT3, COX2, and sEH in the microglia of human premature infants. **A** Representative Western blot analyses for HMOX1, LPCAT3, COX2, FTH1, P53, 15-LOX, and sEH. The assay was performed on brain homogenates from the germinal matrix or white matter of human infants with and without IVH. Adult rat brains were used as positive controls. Values were normalized to β-actin levels. The bar graph shows mean ± s.e.m (*n* = 5 each). The student t-test was used. **B** Representative immunofluorescence of coronal sections from 23-week premature infants with and without IVH labeled with HMOX1, sEH, COX2, or LPCAT3 with Iba1 antibodies. Periventricular germinal matrix and white matter were evaluated, as indicated in the image. Note increased reactivity HMOX1, LPCAT3, COX2, and sEH on the microglia (arrowhead) of infants with IVH relative to controls without IVH. Scale bar, 20 µm.

To assess the translational potential of the study, we measured key enzymes involved in iron, lipid, and oxylipin metabolism in human autopsy samples from preterm infants. Western blot analyses were performed on tissues harvested from the germinal matrix and white matter of premature infants with and without IVH (23-27 gestational weeks). We demonstrated that LPCAT3, HMOX1, and FTH1 were elevated in the germinal matrix of infants with IVH compared to controls without IVH (Fig. 8A). However, 15-LOX, COX2, sEH, and P53 levels were comparable between infants with and without IVH. In the white matter, COX2, sEH, HMOX1, and P53 expressions were higher in infants with IVH relative to controls without IVH (Fig. 8A), but there was no such difference in the levels of 15-LOX and FTH1. There were no differences related to infant sex (Tables S2 and S3).

To identify the molecular mechanisms at the cellular level, we evaluated the expression of HMOX1, LPCAT3, sEH, and COX2 in the microglia and astrocytes by immunolabeling the coronal sections from human samples (Fig. 8B). To this end, we immunolabeled each of these molecules with Iba1- and GFAP-specific antibodies. Although HMOX1 was expressed in the microglia of the white matter and germinal matrix of both infants with and without IVH, HMOX1 reactivity was more extensive in microglia in infants with IVH than in controls. LPCAT3 expression was abundant in the microglia of the germinal matrix of the infants with IVH and was weak-to-absent in infants without IVH. Likewise, COX2 was widely expressed on Iba1^+^ microglia in the white matter of infants with IVH, but was almost absent in infants without IVH. Finally, sEH expression was abundant on the microglia and weak on the astrocytes of kits with IVH. However, sEH was almost absent in astrocytes and microglia of infants without IVH. Together, the occurrence of IVH increased HMOX1, LPCAT3, sEH, and COX2 levels in human infants, just as in rabbit kits, and these proteins were predominantly expressed in microglia. This suggests that microglia play a critical role in IVH-induced ferroptosis and the dysregulation of lipid metabolism.

## Discussion

The present study identified that IVH activated the enzymes cPLA2 and LPCAT3, which accelerated the Lands cycle and led to an accumulation of lysophospholipids and free fatty acids. IVH also upregulated COX, LOX, and CYP enzymes, increasing oxylipin levels. Moreover, IVH activated the DGAT1 enzyme, which raised TAG production. These metabolic changes triggered by IVH resulted in ferroptosis, apoptosis, and inflammation. Importantly, iron chelation by DFX treatment reversed each of these pathological changes. DFX therapy downregulated the levels of cPLA2 and LPCAT3 enzymes (decelerating the Lands cycle), reducing the levels of lysophospholipids and free fatty acids. Additionally, it reduced the expression of the DGAT1 enzyme, diminishing TAG generation. Furthermore, DFX inhibited COX, LOX, and CYP enzymes, attenuating oxylipin production. Consistently, DFX treatment mitigated ferroptosis, inflammation, myelination failure, and neurological dysfunction. In line with findings from our rabbit model, analyses of autopsy samples from premature infants revealed evidence of ferroptosis and apoptosis. Additionally, human infants with IVH exhibited elevated levels of enzymes involved in phospholipid metabolism and eicosanoid/oxylipin production. These observations deepen our understanding of how iron excess impacts lipid metabolism and identify the mechanisms by which iron chelation induces recovery, highlighting the translational potential of this study.

In this study, we identified specific groups of enzymes, biochemical pathways, and neural cells (specifically microglia) that are activated when there is an excess of iron. Many enzymes involved in lipid metabolism depend on iron; for instance, those responsible for synthesizing fatty acids, forming cholesterol, and metabolizing phospholipids [45]. We report an increased expression of enzymes that regulate the Lands Cycle and oxylipin production in premature human infants and rabbit kits with IVH. These distinct changes in enzyme expression may be related to increased transcription or post-translational modifications. Furthermore, iron in its ferrous form can indirectly affect enzyme expression by inducing oxidative stress and inflammation [45]. Therefore, therapies aimed at restoring iron homeostasis in the brain are crucial for survivors of IVH.

The phospholipid composition of fatty acyl chains is regulated by the reacylation and deacylation pathways called the Lands cycle. The deacylation is catalyzed by PLA2, while the reacylation occurs through LPCAT3 enzymes. We demonstrated pathological acceleration in the Lands cycle in IVH. The cPLA2 enzymes were upregulated in kits with IVH, hastening the deacylation of fatty acyl chains. This process resulted in the release of lysophospholipids and PUFA (specifically arachidonic acids, 20:4). The IVH-activated LPCAT3 enzyme catalyzed reacylation, elevating levels of PE, PS, and PG. The accumulation of PUFA contributed to increased oxylipin levels in kits with IVH. Similar disruptions in the Lands cycle have been reported in some neurological conditions, including Alzheimer’s disease, Parkinson’s disease, and stroke [9, 13]. Importantly, DFX treatment in rabbits with IVH decelerated the Lands cycle by downregulating LPCAT3 and cPLA2 enzymes, which in turn reduced lysophospholipid and PUFA levels. A decline in PUFA (n-3 and n-6) would reduce oxylipin production and alleviate inflammation [9]. Furthermore, the activation of cPLA2 and LPCAT3 has been linked to the development of neurological diseases. For example, cPLA2 activation plays a critical role in the pathogenesis of ischemic stroke, Alzheimer’s disease, Parkinson’s disease, and traumatic brain injuries [46–48]. Elevated levels of LPCAT3 are also associated with damage in subarachnoid hemorrhage and dementia [49–51]. Together, these studies identified a series of findings: IVH activates the enzymes involved in the Lands cycle and oxylipin synthesis, leading to the production of eicosanoids, lysophospholipids, and oxidized phospholipids, all of which would contribute to white matter injury in newborns with IVH. Importantly, each of these adverse effects can be mitigated by iron chelation with DFX.

Another novel finding was a significant accumulation of TAGs in kits with IVH and rescue through DFX treatment. We also observed lipid droplet (triacylglycerol, TAG) accumulation in the microglia of kits with IVH, which was mitigated by DFX treatment. The IVH-triggered TAG accumulation was linked to increased expression of DGAT1, while DFX-induced TAG depletion was attributed to reduced levels of these enzymes [14]. In line with our studies, Zika virus infection enhances DGAT1-mediated TAG synthesis and lipid droplet formation in neuronal stem cells [17]. However, this association has not been established in any animal model of neurological disorder. TAG accumulation in the microglia may serve as a crucial PUFA reservoir, contributing to oxylipin generation and ferroptosis in newborns with IVH [52]. Together, DFX-induced depletion of TAG is a vital and previously unknown mechanism by which DFX could offer neuroprotection in IVH survivors.

Our studies revealed that IVH accelerated the production of many oxylipins by activating enzymes of COX, LOX, and CYP-450 pathways; these reactions were blocked by DFX treatment. Additionally, DFX downregulated the protein levels of soluble epoxy hydrolase (sEH) and reduced the number of microglia expressing sEH. The sEH enzyme degrades the beneficial and anti-inflammatory epoxy fatty acids (EETs) into dihydroxy-eicosatrienoic acids (DHETs). Therefore, inhibiting sEH through DFX could lead to decreased cerebral inflammation and injury in survivors of IVH. Consistent with our studies, sEH levels are elevated in the brains of patients with Parkinson’s disease (PD), and pharmacological inhibition of the sEH enzyme or silencing the sEH gene shows clinical recovery in a mouse model of PD [53]. Although oxylipins are known to mediate inflammatory response directly, the emerging concept is that eicosanoids/oxylipins can be incorporated into phospholipids by the enzymes of the Lands cycle [9]. These enzymatically oxidized phospholipids, formed in immune cells, play crucial roles in ferroptosis, apoptosis, and various diseases. Accordingly, IVH-generated oxylipins would enzymatically oxidize phospholipids, contributing to inflammation. Together, these studies reinforce our novel finding that DFX-triggered inhibition of 15-LOX, COX-2, and sEH enzymes promotes neurological recovery by reducing the generation of pro-inflammatory oxylipins or enzymatically activated phospholipids.

Ferroptosis occurs when lipid peroxides accumulate by two main mechanisms: iron-catalyzed free-radical lipid peroxidation and an enzymatically regulated process catalyzed by lipoxygenases (15-LOX). Iron is a critical component of lipoxygenase and several other enzymes, including cytochrome P450, xanthine oxidase, NADPH oxidases, mitochondrial complexes I and III, as well as catalase and peroxidases [54]. Therefore, iron chelation not only affects iron-catalyzed lipid peroxidation but could also impact the activity of iron-containing enzymes. It is challenging to differentiate the damaging effects of iron-catalyzed non-enzymatic and enzymatic reactions. The present study found that DFX inhibited ferroptosis by reducing iron. Indeed, DFX decelerates the Fenton reaction, generation of reactive oxygen species, and subsequent lipid peroxidation. Moreover, DFX treatment reduced cytoplasmic phospholipase A2 (PLA2) levels, thereby increasing the production of polyunsaturated fatty acids (PUFAs). Additionally, the expressions of 15-LOX, COX2, and sEH enzymes were downregulated, thereby reducing eicosanoid production. This decline in iron-containing enzymes, such as COX2 and 5-LOX, may be linked to reduced iron availability induced by DFX treatment. These findings are consistent with the observed decrease in 15-LOX levels in chondrocytes treated with DFX in culture experiments [55]. It is important to note that DFX treatment did not affect key enzymes of the GPX4-dependent ferroptotic pathway, including GPX4 and SLC7A11. Among the GSH-independent pathways that were not evaluated in this study are inducible nitric oxide synthase (iNOS), guanosine triphosphate cyclohydrolase 1 (GCH1), dihydroorotate dehydrogenase (DHODH), prominin-2, monounsaturated fatty acids (MUFA), and other regulatory pathways [56]. Future studies exploring these pathways could provide further insights into the mechanisms related to DFX.

DFX treatment alleviated WMI and neurological dysfunction in the present study. These findings are reinforced by the previous studies, in which DFX treatment reduces white matter loss and inflammation in adult rat intracranial hemorrhage models [57, 58]. Likewise, a study using a piglet model of intracranial hemorrhage has shown that DFX therapy reduces inflammation and WMI [59]. Since DFX is an FDA-approved iron chelator for transfusion-dependent anemias in adults and children [60], present and previous studies strengthen the rationale of DFX treatment in infants with IVH.

DFX is an FDA-approved drug that has shown potential in both preclinical and clinical studies involving brain hemorrhage. However, the literature reports mixed findings on DFX’s ability to cross the blood-brain barrier (BBB) [61]. A systemic reduction in iron levels may also contribute to decreased iron concentration in the brain. The half-life of DFX in humans is approximately 8 h, although it is unknown in rabbits. Rodents generally exhibit a higher drug metabolism [62]. Therefore, we used a higher dose and administered it twice daily. DFX chelates ferric iron and hemosiderin, forming a water-soluble complex, which is eliminated in the urine. DFX also chelates aluminum and can reduce aluminum-induced free radical generation. In adult models of brain hemorrhage, systemic administration of DFX confers protection [59, 63, 64]. A clinical trial involving 291 patients with intracranial hemorrhage revealed that treatment with DFX accelerated and positively influenced neurological recovery [21]. Although DFX has several known adverse effects, including allergy to this compound, headache, dizziness, cough, hearing impairment, and acute renal failure, the benefit of the DFX treatment is remarkable. Together, our revelation of DFX-induced reduction in ferroptosis, WMI, and improvement in neurological function, based on solid mechanistic underpinnings, sets the stage for a clinical trial of DFX in premature infants with IVH. Moreover, identifying a new mechanism of DFX-driven change in lipid and eicosanoid/oxylipin metabolism will open new avenues of research and treatment for conditions where iron overload causes tissue damage to the brain or other organs.

## Materials and methods

### Human tissues

The Institutional Review Board of Albert Einstein College of Medicine, Bronx, NY, reviewed and approved the use of postmortem brain samples from premature infants for the present study (Ref# 2019-10439). Informed consent was obtained from the parents before obtaining autopsy samples. We followed all the guidelines and regulations of our institution’s Human Rights Committee. The postmortem samples comprised forebrain tissue, which were taken from premature infants with and without IVH of 23-27 gestational weeks. These infants were ≤7 days old, and the autopsy samples were harvested within 18 h of their demise. We excluded those premature infants who suffered hypoxic-ischemic encephalopathy, meningitis, culture-proven sepsis, major brain or spinal cord malformation, and chromosomal defects. The wall of the cerebral hemisphere in premature infants comprised the ventricular zone (VZ), subventricular zone (SVZ), intermediate zone, cortical plate, and marginal zone, as described by the Boulder Committee. In the present manuscript, we use the terms “intermediate-zone embryonic white matter” interchangeably with “white matter,” and “germinal matrix” with “ganglionic eminence.” The details of autopsy samples are in Extended Data Table S1.

### Human tissue collection and processing

We processed human tissues as in our prior studies [29]. About 3-4 mm-thick Coronal slices were cut at the level of the head of the caudate nucleus from the frontal-parietal lobe. The coronal blocks included the cortical plate, embryonic white matter, and ganglionic eminence. The samples were placed in 4% paraformaldehyde in PBS (0.1 M, pH 7.4) for 18 h and then cryoprotected by incubating them in a 15% sucrose solution in PBS, followed by a 30% sucrose solution in PBS. The tissues were then frozen after embedding in optimum cutting temperature compound (Sakura, Japan). Frozen coronal blocks were sectioned into 15-μm-thick slices using a cryostat.

### Animals

The Institutional Animal Care and Use Committee of Albert Einstein College of Medicine, Bronx, NY, reviewed and approved this study (ref #00001150). We followed all methods in accordance with the Animal Care and Use Committee’s guidelines. We employed our well-established E29 rabbit model of glycerol-induced IVH, which has been extensively published [2, 3, 29, 30, 65]. We purchased timed-pregnant New Zealand rabbits from Charles River Laboratories, Inc. (Wilmington, MA), and performed a C-section to deliver the preterm kits at 29 days of gestation (full-term = 32 days). Neonatal kits were cared for in an infant incubator at 35^o^C. To induce IVH, we injected rabbit kits at four hours of age of either sex with 50% glycerol (6.5 gm/kg) intraperitoneally. Intraperitoneal glycerol results in IVH by causing intravascular dehydration and elevating serum osmolality, which diminishes intracranial pressure and ruptures fragile vessels in the ganglionic eminences [29]. We used rabbit milk replacer (Wombaroo, Glen Osmond, Australia) to gavage-feed the kits in a volume of 3-4 ml every 12 h (100 ml/kg/day) during the first two days, and then feeds were enhanced to 125,150, 200, 250 and 280 ml/kg at postnatal days 3, 5, 7, 10 and 14, respectively. We quantified the severity of IVH by measuring ventricular volume (length, breadth & depth in coronal & sagittal views) on head ultrasound at 24 h age using an Acuson X700 (Siemens) ultrasound machine. Based on ventricular size, kits with IVH were graded as moderate (70-150 mm^3^) or severe (151–250 mm^3^) IVH. A ventricular volume <70 mm^3^ was considered as either an absence of IVH or the presence of small or microscopic hemorrhage (glycerol-treated without IVH). The kits with moderate-to-severe IVH of either sex were randomized into treatment or control groups in such a manner that the severity of IVH was similar between the comparison groups. Although IVH occurs more frequently in males, IVH-induced WMI is not associated with the male or female sex. Hence, we included animals of both sexes in the study. We excluded kits with cardiorespiratory arrest requiring resuscitation, diarrhea, failure to gain weight, and seizures.

### DFX treatment

Rabbit kits with IVH were randomized into two treatment groups: DFX (deferoxamine mesylate, Fresenius Kabi, IL, USA) and Saline. DFX was administered at 50 mg/kg twice daily IM for seven days or earlier if kits were euthanized before seven days.

### Rabbit tissue collection and processing

We processed the tissues from the rabbit kits as described in previous studies [29]. The brain slices were fixed into 4% paraformaldehyde in phosphate-buffered saline overnight and were cryoprotected by immersing them into 15% sucrose in PBS buffer for 24 h and followed by 30% sucrose for the following 24 h. Next, we froze the tissue slices after embedding the tissues into an Optimum Cutting Temperature compound (Fisher Health Care, Houston, TX). Frozen coronal blocks were cut into 18 µm thick coronal sections on the cryostat. For Western blot analyses, a 2 mm thick coronal slice was harvested at the level of the mid-septal nucleus from the cerebral hemisphere.

The detailed methods are provided in the Supplementary Data file for immunohistochemistry, Stereological quantification of myelination, astrogliosis, and microglia, quantification of ferroptosis and TUNEL^+^ cells, Iron staining (Prussian Blue staining), Western blot analyses, Quantitative Real-Time Polymerase Chain Reaction (qRT-PCR), Dissection of MGE, RNA seq, and analyses, Neurobehavioral assessment, Lipidomic and eicosanoid studies, Neutral lipid staining, and measurement of Isoprostanes, isofurans and glutathione.

### Statistical analyses

We selected a sample size of 5 (each group) to assess the effect of IVH and DFX treatment based on the mean and standard deviation, achieving a power of greater than 80% and a *p*-value of less than 0.05. The data are presented as means ± standard error of the means (SEM). To compare the TUNEL^+^ and TfR1^+^ cells as well as western blot analyses in human infants with and without IVH, we used Student’s t-test or Mann-Whitney U test. We used one-way ANOVA to compare iron staining at D3, D7, and D14. To compare TfR1^+^ cells at D3 and D7, we employed repeated measures of ANOVA. All two-group comparisons for IVH vs. no IVH and DFX vs. vehicle treatment were done, using the Student t-test (normal distribution) or Mann-Whitney U test (not normal distribution). For all three-group comparisons, either ANOVA (parametric) or Kruskal-Wallis test (non-parametric test) was applied. Where ANOVA was employed, all post hoc comparisons between means were done by Tukey’s multiple comparison test at *P* < 0.05.

To account for biological variance attributable to sex in mixed-sex cohorts, we compared groups using Analysis of Covariance (ANCOVA) with “group” as the fixed factor and “sex” as a covariate. The dependent variable was modeled as a linear function of treatment Group and Sex, following the structure: Outcome ~ Group + Sex. All statistical assumptions, including normality of residuals and homogeneity of regression slopes, were verified. Analyses were performed using custom Python scripts with statsmodels v0.14.6 and scipy v1.16.3 libraries. Statistical parameters, including *P*-values, F-values, degrees of freedom (DF), and variance, are reported in Tables S2 and S3.

## Supplementary information

Supplementary material

REPRODUCIBILITY CHECK LIST

Uncropped Western blots

RT PCR DATA--IVH vs no IVH at D3

RT-PCR DATA --EFFECT IF DFX treatment

Dataset 1

## Data Availability

We will share all data and original code reported in this paper. Raw scRNA-seq data will be available in the Gene Expression Omnibus (GEO). Lipidomic and eicosanoid assays are attached to the manuscript. The corresponding author is the primary point of contact.
