# Supplementary material for: Iron chelation targets lipid metabolism to reduce white matter injury in germinal matrix hemorrhage
Source: Cell Death Dis. 2026 May 23;17(1):648. doi: 10.1038/s41419-026-08866-z (PMC13376222; doi:10.1038/s41419-026-08866-z)
Supplement: Supplementary file 1 — Supplementary material [file 41419_2026_8866_MOESM1_ESM.docx]

**Supplementary Information for**

**Iron chelation targets lipid and oxylipin metabolism to reduce white matter injury in germinal matrix hemorrhage**

Bokun Cheng;^1,2^ Akanksha Mishra,^1,2^ Xusheng Zhang, ^3^ Zaw Myo Hein,^4^ Nourelhoda Gouda,^1^ Karen Schaeffer,^1^ Mikhail Kislin,^2^ Yunping Qiu, ^5^ Fereshteh Zandkarimi,^6^ Irwin Kurland,^5^ Praveen Ballabh,^1,2^

^1^Department of Pediatrics, Albert Einstein College of Medicine, Bronx, NY; ^2^**Dominick** P**. Purpura** Department of Neuroscience, Albert Einstein College of Medicine, Bronx, NY; ^3^Computational Genomics Core, Albert Einstein College of Medicine, Bronx, NY, ^4^College of Medicine, Ajman University, Ajman, UAE; ^5^[Department of Medicine](https://einsteinmed.edu/departments/medicine/) (Endocrinology), Albert Einstein College of Medicine, Bronx, NY; ^6^Department of Chemistry, Columbia University, NY 10027

**Number of Supplementary Figures: Fig. S1-9**

**Tables:** **Supplementary Table:** S1

**Methods** (Supplementary methods)

**
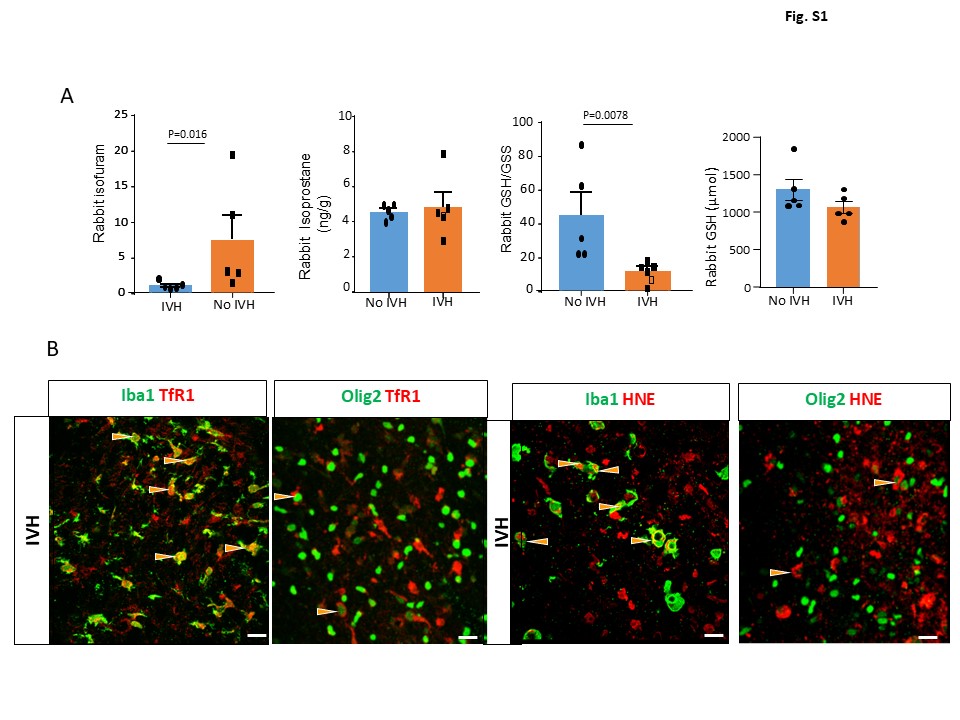
Fig. S1: A) IVH induces oxidative stress and ferroptosis.**  Isoprostane, isofuran, glutathione (GSH/GSSG), and GSH levels were measured in kits with and without IVH at D3. Data are mean ± SEM. The Student's t-test was used. The Isofuran and GSH/ GSSG ratio was elevated in IVH. B) TfR1 or 4-HNE is expressed more frequently in the microglia but less often on Olig2^+^ oligodendrocytes. Double labeling of coronal sections from kits with IVH (D3) using a) TfR1 with Iba1 or Olig2 and b) 4HNE with Iba1 or Olig2 antibodies**.** Arrowheads show microglia and oligodendrocytes undergoing ferroptosis. Scale bar, 20 µm.

**
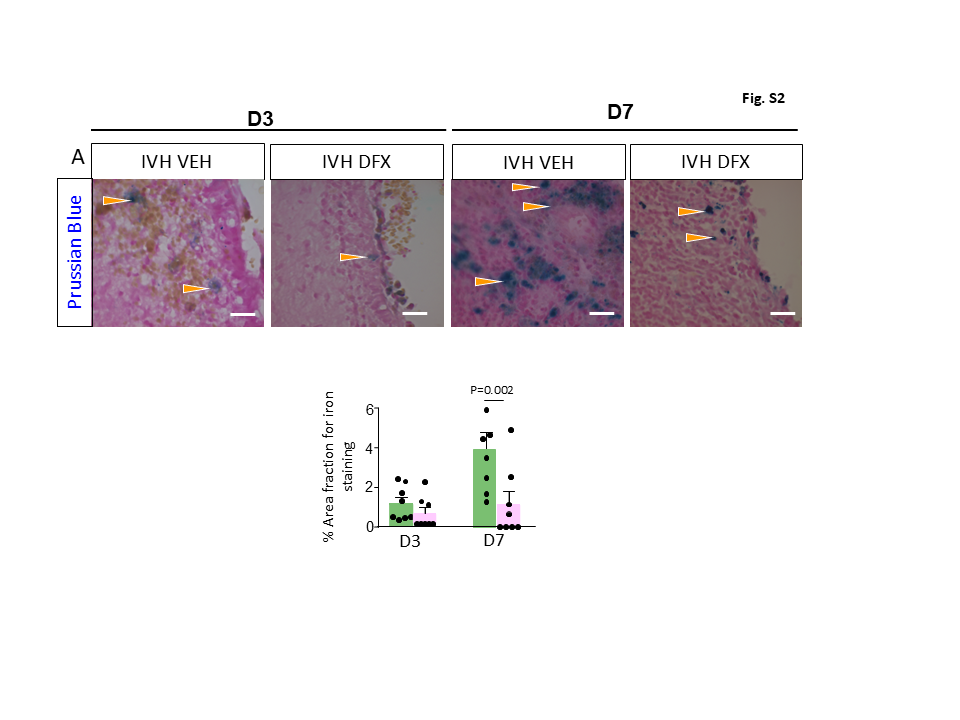
Fig. S2. Deferoxamine (DFX) treatment reduces iron load. A)** Representative Prussian blue staining of coronal sections from the periventricular region of DFX- and vehicle-treated kits with IVH shows iron labeling at D3 and D7. Note reduced iron staining in DFX-treated kits relative to controls. The bar graph shows mean ± s.e.m (n=7-10 each). Two-way ANOVA was used. Scale bars, 20 µm. **B)  
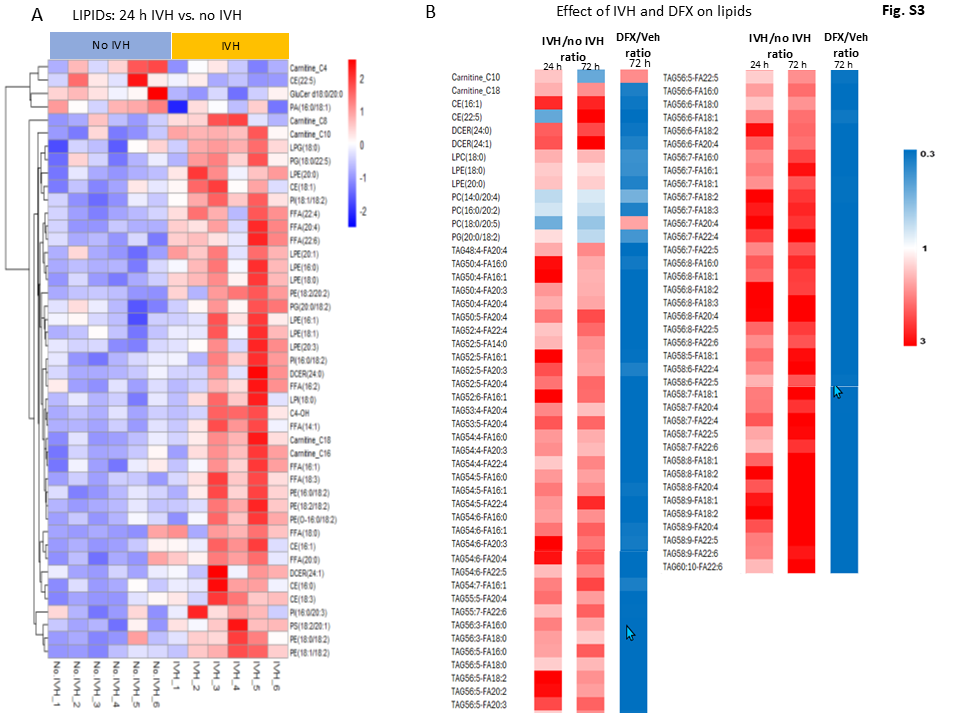
**

**Fig. S3: IVH elevates many lipids at 24 h age, and DFX treatment rescues. A)** The heatmap illustrates the effect of IVH on lipid levels at 24 hours of age. Note that most of the lipids are elevated in kits with IVH. The annotation on the X-axis shows DFX vs. saline. Annotations on the Y-axis depict the type of lipids. The red, white, and blue colors represent high, average, and lower expression of lipids, respectively. **B)** The heatmap shows the ratio of lipid levels of IVH and no IVH (IVH/no IVH) kits at 24 and 72 h age (effect of IVH) as well as the ratio for DFX and saline treatment (IVH, DFX/ IVH, saline Annotation on the Y-axis depicts the name of lipid and the ratio of their concentration for IVH/no IVH at 24, 72 h, and the ratio of lipids for DFX/saline treatment. The red and blue colors represent elevated and reduced ratios of lipids, respectively. Note that several of the lipids are elevated in kits with IVH at 24 and 72 hours of age, and DFX therapy reduces their levels

**
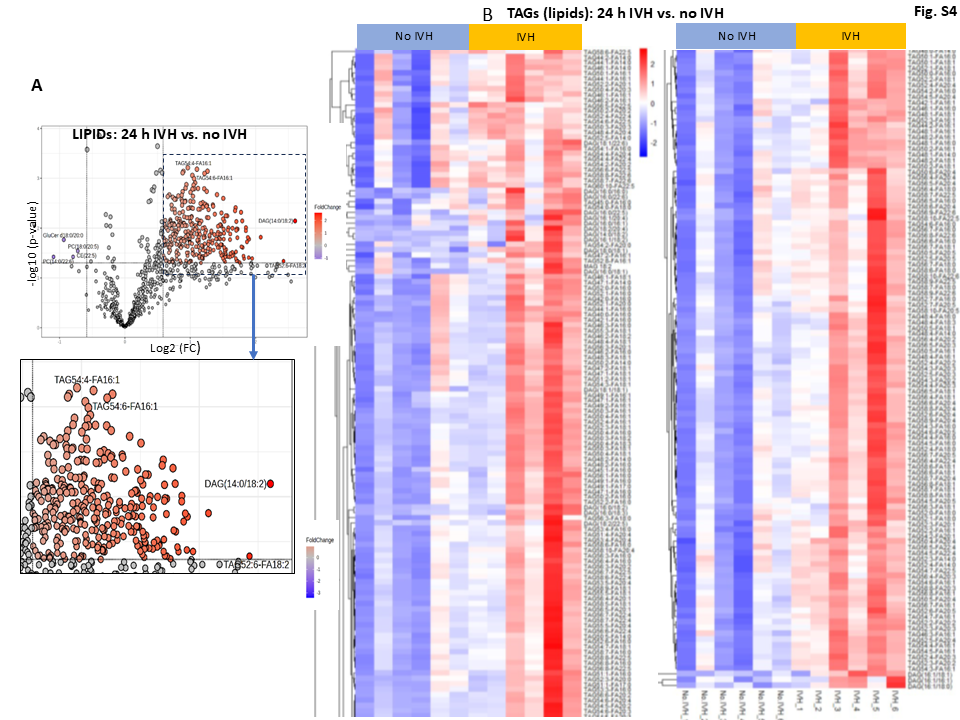
Fig. S4: IVH elevates TAGS at 24 h, which DFX treatment reduces. A)** Volcano plots illustrate the effect of IVH on the lipid profile at 24 hours. Log2 (FC) values are plotted on the X-axis and -log10 (p-value) on the Y-axis. Note TAGs are elevated in IVH at 24 h of age. Lower panel is the magnified view of the boxed area in the upper panel for better visiblity. **B)** The heatmap illustrates the effect of IVH on TAG levels at 24 hours of age. The annotations on the x-axis indicate IVH versus no IVH. Annotations on the Y-axis depict the type of TAGs. The red, white, and blue colors represent high, average, and lower expression of TAGs, respectively**.**

**
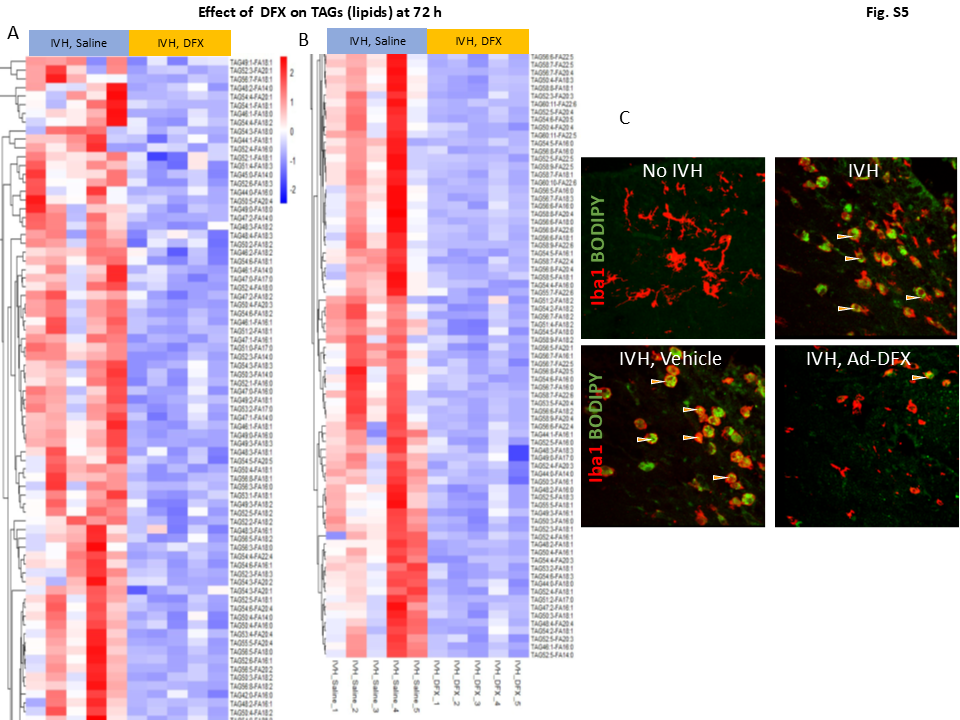
Fig. S5: A,B) The effect of DFX on the levels of TAGs at 72 h of age (Heat-Map).** Note that the TAGs are reduced with DFX treatment. The annotation on the X-axis indicates DFX versus saline at 72 hours of age. Annotations on the y-axis indicate the type of tags. The red, white, and blue colors represent high, average, and lower expression of TAGs, respectively. **C)** Double immunolabeling of rabbit brain sections with Iba1 and BODIPY (lipid droplets) shows lipid droplets in microglia of kits with IVH. DFX treatment reduces lipid droplets after DFX treatment.

**
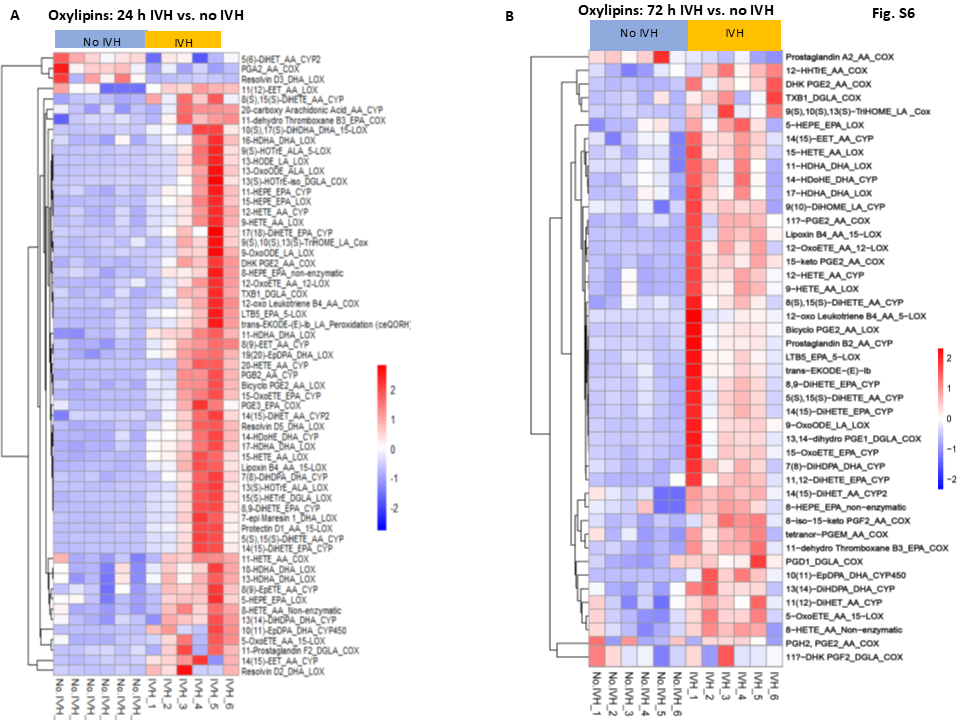
Fig. S6: IVH elevates oxylipins (eicosanoids) at 24 and 72 hours of age. A, B)** The heatmap shows the effect of IVH on the eicosanoid levels at 24 h and 72 h of age. The X-axis represents IVH vs. no IVH at 24 and 72 h of age. Annotations on the Y-axis depict the type of eicosanoids. The red, white, and blue colors represent high, average, and lower expression of eicosanoids, respectively. Note that all eicosanoids are elevated in kits with IVH.

**
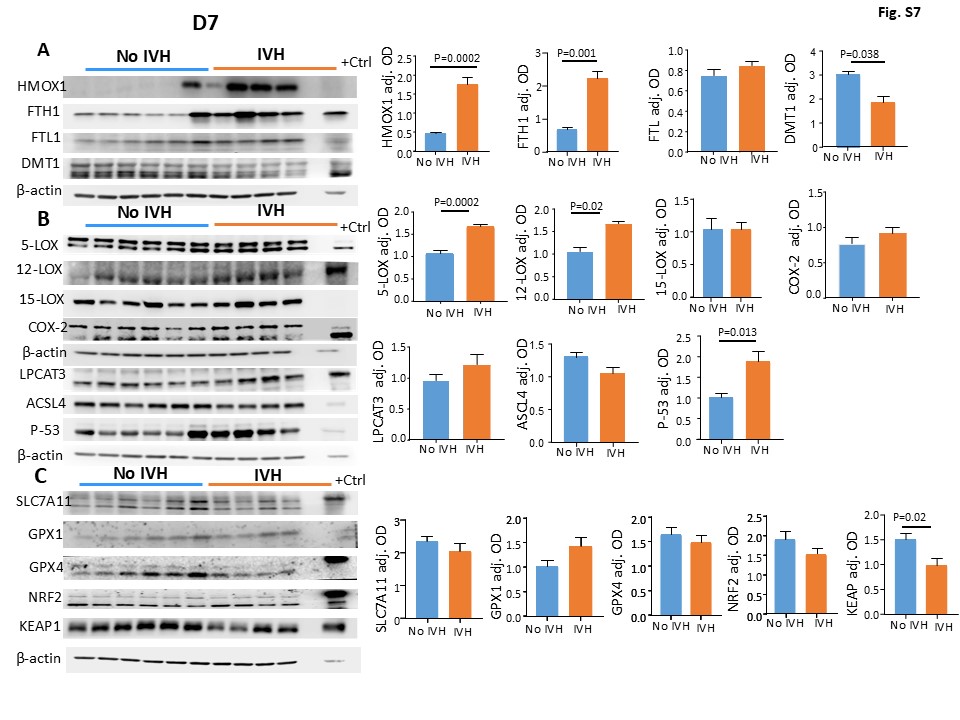
Fig. S7: IVH increases HMOX1, FTH1, 5-LOX, 12-LOX, and P53 at D7. A)** Representative Western blot analyses for molecules playing key roles in iron metabolism, including HMOX1 (28-33kD), FTH1(21kD), FLT1(19kD), and DMT1 (55/70/100kD). The assay was performed on brain homogenates from kits with and without IVH at D7. Adult rat brains were used as positive controls. Values were normalized to β-actin levels. The bar graph shows mean ± s.e.m (n=5 each). The student t-test was used. **B)** Typical Western blot analyses for molecules inducing ferroptosis, including 5-LOX (78kD), 12-LOX (76kD), 15-LOX (75kD), COX2 (70kD), LPCAT3 (52kD), ASCL4 (75kD), and P53 (53kD). The assay was performed on brain homogenates from kits with and without IVH at D7. Adult rat brains were used as positive controls. Values were normalized to β-actin levels. The bar graph shows the mean ± s.e.m. (n = 5 each). The student t-test was used. **C)** Representative Western blot analyses for molecules that protect against ferroptosis, including SLC7A11 (35kD), GPX1 (22kD), GPX4 (20/22kD), KEAP1 (60-64 kD), and NRF2 (97- 100kD). The assay was performed on brain homogenates from IVH and non-IVH kits at D7. Adult rat brains were used as positive controls. Values were normalized to β-actin levels. The bar graph shows the mean ± s.e.m. (n = 5 each). The student t-test was used

**
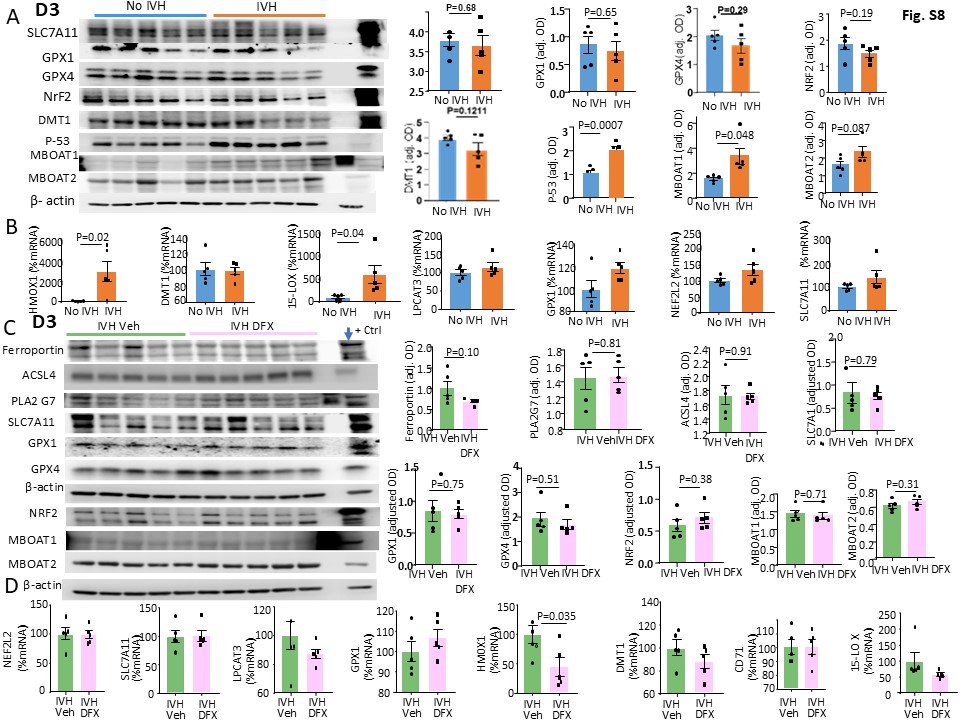
**

**Fig. S8. Effect of IVH and DFX on key molecules playing a role in oxidative stress and ferroptosis at D3. A)** Representative Western blot analyses on brain homogenates from kits with IVH vs. without IVH for molecules regulating oxidative stress and ferroptosis, including SLC7A11 (35kD), GPX1 (22kD), GPX4 (20/22kD), KEAP1 (60-64 kD), P53 (53kD), DMT1 (70kD), NRF2 (97- 100kD), MBOAT1 (56kD), and MBOAT2 (57kD) at D3. Student T-test was employed. Values were normalized to β-actin levels. The bar graph shows mean ± s.e.m (n=5 ea). **B)** Gene expression was assayed by real-time qPCR using kits with and without IVH, with TaqMan probes. Data are mean ± s.e.m. (n=5).  HMOX1 and 15-LOX were elevated in kits with IVH. P-values are indicated. **C)** Representative Western blot analyses for brain homogenates from IVH kits treated with DFX or vehicle for molecules regulating oxidative stress and ferroptosis, including ferroportin (63kD), SLC7A11 (35kD), PLA2G7 (29kD), ASCL4 (75kD), GPX1 (22kD), GPX4 (20/22kD), NRF2 (97-100kD), MBOAT1 (56kD), and MBOAT2 (57kD). The rat brain was taken as positive control. Values were normalized to β-actin levels. The bar graph shows the mean ± s.e.m (n = 5 each); a Student T-test was employed. **D)** Gene expression was assayed by real-time qPCR using IVH kits treated with DFX or vehicle, and TaqMan probes were employed. Data are mean ± s.e.m. (n=5).  HMOX1 was reduced in DFX-treated kits. P-values are indicated.

**
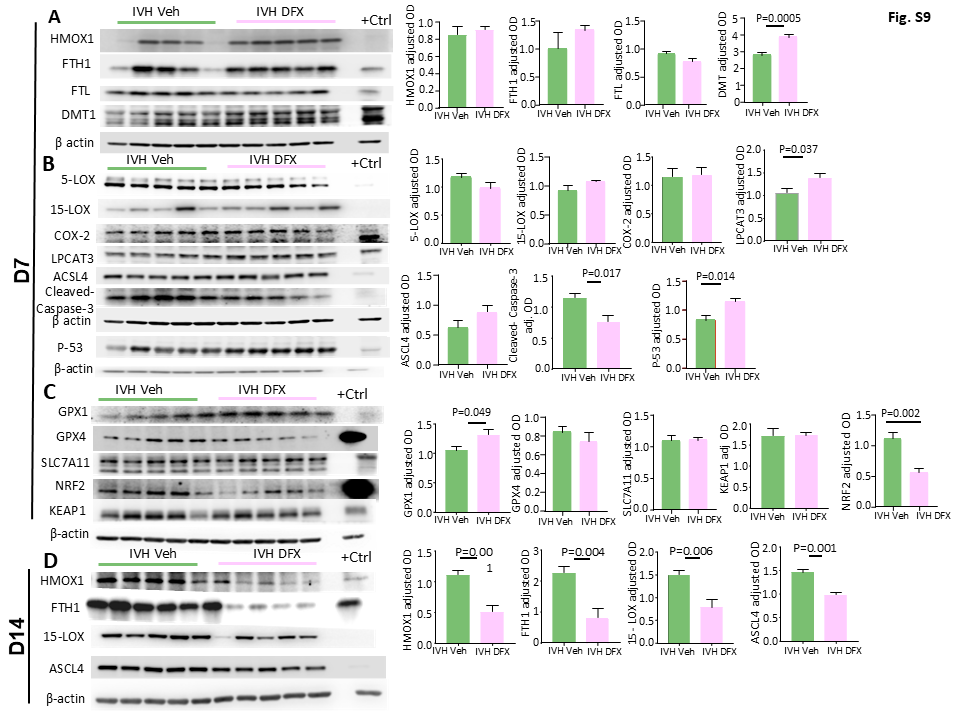
**

**Fig. S9: DFX treatment elevated DMT1, P53, and GPX4 and diminished HMOX1, FTH1, and 15-LOX at D14. A)** Representative Western blot analyses for molecules playing key roles in iron metabolism, including HMOX1 (28-33kD), FTH1(21kD), FLT1(19kD), and DMT1 (55/70/100kD). The assay was performed on brain homogenates from DFX- and vehicle-treated kits at D7. Adult rat brains were used as positive controls. Values were normalized to β-actin levels. The bar graph shows the mean ± s.e.m. (n = 5 each). The student t-test was used. **B)** Typical Western blot analyses for molecules that induce ferroptosis include 5-LOX (78kD), 15-LOX (75kD), COX2 (70kD), LPCAT3 (52kD), ASCL4 (75kD), cleaved caspase 3 (17-19kD), and P53 (53kD). The assay was performed on brain homogenates from DFX- and vehicle-treated kits at D7. Adult rat brains were used as positive controls. Values were normalized to β-actin levels. The bar graph shows the mean ± s.e.m. (n = 5 each). The student t-test was used. **C)** Representative Western blot analyses for molecules that protect against ferroptosis, including SLC7A11 (35kD), GPX1 (22kD), GPX4 (20/22kD), KEAP1 (60-64 kD), and NRF2 (97-100kD). at D7. The assay was performed on brain homogenates from DFX- and vehicle-treated kits at D7. Adult rat brains were used as positive controls. Values were normalized to β-actin levels. The bar graph shows mean ± s.e.m (n=5 ea). The student t-test was used. **D)** Typical Western blot analyses for molecules preventing ferroptosis include HMOX1 (28-33kD), FTH1(21kD), 15-LOX (75Kd), and ASCL4 (75kD) at D14. The assay was performed on brain homogenates from DFX- and vehicle-treated kits at D14. Adult rat brains were used as positive controls. Values were normalized to β-actin levels. The bar graph shows mean ± s.e.m (n=5 ea). The student t-test was used.

**Table S1:** Demographics of human infants with and without IVH

Post-conceptional age Sex Birth weight (g) IVH / No IVH Cause of death

(Weeks)

_______________________________________________________________________

| 25 | Male | 690 IVH grade 3 Respiratory failure |
| --- | --- | --- |
| 24 | Female | 610 IVH grade 3 Pulmonary hemorrhage |
| 25 | Male | 630 IVH grade 2 Respiratory failure |
| 23 | Female | 520 IVH grade 2 Pulmonary Hemorrhage |
| 23 | Female | 530 IVH grade 4 Respiratory failure |
| 24 | Female | 710 no IVH Respiratory failure |
| 23 | Male | 530 no IVH Cardio-Respiratory failure |
| 26 | Male | 680 no IVH Clinical sepsis |
| 27 | Female 750 no IVH Persistent pulmonary hypertension | |
| 23 | Female 570 no IVH Respiratory failure | |

­­­­­­­­­­­­­­­­­­­­­­­­­­­___________________________________________________________________________________

**Table S2: Effect of sex as a biologic variable by employing ANCOVA (data from Fig. 1-8).**

A) Human data related to Fig. 1 (Microscopic quantification of cells)

| **Outcome** | **comparison** | **P value**  **Group** | **F value**  **Group** | **DF_Group** | **Var%Group** | **P value**  **Sex** | **F value Sex** | **Var%Sex** |
| --- | --- | --- | --- | --- | --- | --- | --- | --- |
| Apoptosis | IVH vs. no IVH | 1.11E-06 | 241.14 | (1, 7) | 97.1 | 0.66397 | 0.21 | 0.1 |
| TfR1^+^ | IVH vs. no IVH | 0.000275 | 81.94 | (1, 5) | 94.2 | 0.80623 | 0.07 | 0.1 |

B) Area faction of iron labeling (Fig. 2B)

| **Para-**  **meter** | **F_Full model** | **DF_**  **Full** | **P_**  **Full** | **P**  **Group** | **F_**  **Group** | **DF_**  **Group** | **Var%**  **Group** | **P_**  **Sex** | **F_**  **Sex** | **Var%**  **Sex** | **Post-Hoc**  **Tukey's Test** | | |
| --- | --- | --- | --- | --- | --- | --- | --- | --- | --- | --- | --- | --- | --- |
| Prussian | 4.08 | (3,20) | 0.021 | 0.01238 | 5.51 | (2, 20) | 33.5 | 0.6229 | 0.25 | 1.2 | D3 vs D7 | D7 vs D14 | D3 vs D14 |
|  |  |  |  |  |  |  |  |  |  |  | 0.013 | 0.025 | 0.983 |

C) Effect of IVH on ferroptosis related to Fig. 2C

|  | Metric | Comparison | P_value  Group | F_value  Group | DF_  Group | Var%_  Group | P_value  Sex | F_  Sex | Var%_  Sex |
| --- | --- | --- | --- | --- | --- | --- | --- | --- | --- |
| D3 | CD71^+^ | no IVH vs IVH | 9.66E-05 | 62.84 | (1, 7) | 85.7 | 0.105589 | 3.45 | 4.7 |
|  | CD71^+^ HNE^+^ | no IVH vs IVH | 0.0036769 | 18.28 | (1, 7) | 64.9 | 0.134101 | 2.87 | 10.2 |
| D7 | CD71^+^ | no IVH. vs IVH. | 0.0003609 | 41.2 | (1, 7) | 85.5 | 0.930162 | 0.01 | 0 |
|  | CD71^+^  HNE^+^ | no IVH vs IVH | 0.0001563 | 53.97 | (1, 7) | 87.6 | 0.441372 | 0.67 | 1.1 |

D) Iron assay for kits without IVH, with IVH, and treated with DFX, using ICM-MS (Fig. 4A)

| **Para-**  **meter** | **F_Full model** | **DF_**  **Full** | **P_**  **Full** | **P_**  **Group** | **F_**  **Group** | **DF_**  **Group** | **Var%**  **Group** | **P_**  **Sex** | **F_**  **Sex** | **Var%**  **Sex** | **Post-Hoc**  **Tukey's Test** | | |
| --- | --- | --- | --- | --- | --- | --- | --- | --- | --- | --- | --- | --- | --- |
| Iron level | 74.68 | (3,11) | 1.34E-07 | 7.40E-08 | 103.37 | (2, 11) | 94.9 | 0.0626 | 4.29 | 0.3 | noIVH vs IVH | noIVH vs IVH, DEF | IVH vs IVH, DEF |
|  |  |  |  |  |  |  |  |  |  |  | <0.001 | 0.0039 | <0.001 |

E) Effect of DFX treatment on kits with IVH on ferroptosis and inflammation (Fig. 4B)

| Metric | Comparison | P_value  Group | F_Group | DF_Group | Var%_Group | P_Value  Sex | F_Sex | Var%_  Sex |
| --- | --- | --- | --- | --- | --- | --- | --- | --- |
| CD71^+^ | DFX vs SALINE | 0.00213 | 22.37 | (1, 7) | 69.4 | 0.135334 | 2.85 | 8.8 |
| CD71^+^ HNE^+^ | DFX vs SALINE | 0.00677 | 14.39 | (1, 7) | 48 | 0.02194 | 8.6 | 28.7 |
| CD11B^+^ | DFX vs SALINE | 0.04588 | 5.87 | (1, 7) | 45.5 | 0.871527 | 0.03 | 0.2 |
| IBA1^+^ | DFX vs SALINE | 0.21075 | 1.9 | (1, 7) | 21.3 | 0.97559 | 0 | 0 |

F) Effect of DFX treatment on myelination (IHC and Western blot), gliosis, and behavior in rabbits (Fig. 4C-F)

| **Parameter** | **Comparison** | **P value**  **Group** | **F value**  **Group** | **DF_Group** | **Var%Group** | **P value**  **Sex** | **F value Sex** | **Var%Sex** |
| --- | --- | --- | --- | --- | --- | --- | --- | --- |
| Myelin  (IHC) | VEH vs DFX | 0.17641 | 2.26 | (1, 7) | 22.5 | 0.401538 | 0.8 | 7.9 |
| GFAP  (IHC) | VEH vs DFX | 0.002762 | 20.35 | (1, 7) | 72.1 | 0.380753 | 0.87 | 3.1 |
| MBP western | VEH vs DFX | 0.019126 | 9.18 | (1, 7) | 49.9 | 0.179033 | 2.23 | 12.1 |
| MAG western | VEH vs DFX | 0.012207 | 11.24 | (1, 7) | 59.1 | 0.411444 | 0.76 | 4 |
| CNPase western | VEH vs DFX | 0.187598 | 2.13 | (1, 7) | 22.3 | 0.530158 | 0.44 | 4.6 |
| GFAP WESTERN | VEH vs DFX | 0.032321 | 7.09 | (1, 7) | 44.7 | 0.224932 | 1.77 | 11.2 |
| Distance travel | VEH vs DFX | 0.034114 | 5.6 | (1, 13) | 30 | 0.786576 | 0.08 | 0.4 |
| Speed | VEH vs DFX | 0.026608 | 6.25 | (1, 13) | 31.3 | 0.410888 | 0.72 | 0.36 |
| Time active | VEH vs DFX | 0.038515 | 5.4 | (1, 12) | 30.9 | 0.78919 | 0.07 | 0.4 |

G) IVH activates key enzymes and DFX treatment rescues at D3, as shown in Fig. 7 (Western blot analyses)

| **Para-meter** | **Com-**  **parison** | **P value**  **Group** | **F value**  **Group** | **DF_**  **Group** | **Var%_**  **Group** | **P value**  **Sex** | **F value**  **Sex** | **Var%_**  **Sex** |
| --- | --- | --- | --- | --- | --- | --- | --- | --- |
| HMOX1 | IVH vs. no IVH | 0.00194 | 23.14 | (1, 7) | 76 | 0.59622 | 0.31 | 1 |
| FTH1. | IVH vs. no IVH | 3.58E-02 | 6.72 | (1, 7) | 35.7 | 0.05882 | 5.08 | 27 |
| FTL. | IVH vs. no IVH | 0.67995 | 0.19 | (1, 7) | 2.6 | 0.83365 | 0.05 | 0.7 |
| FSP1. | IVH vs. no IVH | 0.49205 | 0.54 | (1, 6) | 7.6 | 0.50146 | 0.51 | 7.3 |
| LPCAT3. | IVH vs. no IVH | 0.00042 | 39.03 | (1, 7) | 82.5 | 0.29363 | 1.29 | 2.7 |
| DGAT1. | IVH vs. no IVH | 0.00517 | 16.02 | (1, 7) | 69.4 | 0.81951 | 0.06 | 0.2 |
| DGAT2. | IVH vs. no IVH | 0.08451 | 4.04 | (1, 7) | 34.1 | 0.40566 | 0.78 | 6.6 |
| cPLA2. | IVH vs. no IVH | 0.00517 | 16.02 | (1, 7) | 69.4 | 0.81951 | 0.06 | 0.2 |
| PLA2G7. | IVH vs. no IVH | 0.00042 | 39.11 | (1, 7) | 83.3 | 0.39302 | 0.83 | 1.8 |
| 5-LOX | IVH vs. no IVH | 0.00184 | 23.59 | (1, 7) | 73.7 | 0.27531 | 1.4 | 4.4 |
| 12-LOX | IVH vs. no IVH | 0.00949 | 12.52 | (1, 7) | 60.8 | 0.33488 | 1.07 | 5.2 |
| 15-LOX | IVH vs. no IVH | 0.00124 | 27.12 | (1, 7) | 68.2 | 0.04935 | 5.63 | 14.2 |
| COX2. | IVH vs. no IVH | 0.00598 | 15.12 | (1, 7) | 65.2 | 0.33478 | 1.07 | 4.6 |
| CY4A | IVH vs. no IVH | 0.01653 | 9.82 | (1, 7) | 58.4 | 0.96978 | 0 | 0 |
| sEH | IVH vs. no IVH | 0.00072 | 32.6 | (1, 7) | 77.2 | 0.14745 | 2.65 | 6.3 |

Effect of DFX treatment

| **Parameter** | **Comparison** | **P_Group** | **F_Group** | **DF_Group** | **Var%_Group** | **P_Sex** | **F_Sex** | **Var%_Sex** |
| --- | --- | --- | --- | --- | --- | --- | --- | --- |
| HO1. | DFX vs Saline | 0.08542 | 4.01 | (1, 7) | 35.7 | 0.65299 | 0.22 | 2 |
| FTH1. | DFX vs Saline | 0.12913 | 2.96 | (1, 7) | 29.7 | 0.92508 | 0.01 | 0.1 |
| FTL | DFX vs Saline | 0.01914 | 9.17 | (1, 7) | 56 | 0.66056 | 0.21 | 1.3 |
| LPCAT3. | DFX vs Saline | 0.66117 | 0.21 | (1, 7) | 2.2 | 0.17100 | 2.33 | 24.4 |
| DGAT1. | DFX vs Saline | 0.00322 | 19.21 | (1, 7) | 73.3 | 0.99228 | 0 | 0 |
| DGAT2. | DFX vs Saline | 0.09360 | 3.76 | (1, 7) | 33.4 | 0.50726 | 0.49 | 4.3 |
| cPLA2. | DFX vs Saline | 0.00322 | 19.21 | (1, 7) | 73.3 | 0.99228 | 0 | 0 |
| p53. | DFX vs Saline | 0.79737 | 0.07 | (1, 7) | 0.8 | 0.2600 | 1.5 | 17.5 |
| caspase3. | DFX vs Saline | 0.0056 | 15.52 | (1, 7) | 68.8 | 0.8284 | 0.05 | 0.2 |
| 5lox | DFX vs Saline | 0.68617 | 0.18 | (1, 7) | 2.4 | 0.60211 | 0.3 | 4 |
| 12-lox | DFX vs Saline | 0.44593 | 0.65 | (1, 7) | 7.9 | 0.45956 | 0.61 | 7.4 |
| 15-lox | DFX vs Saline | 0.25493 | 1.54 | (1, 7) | 14.3 | 0.18158 | 2.2 | 20.5 |
| cyp4a | DFX vs Saline | 0.27599 | 1.4 | (1, 7) | 16.1 | 0.60094 | 0.3 | 3.4 |
| sEH. | DFX vs Saline | 0.80868 | 0.06 | (1, 6) | 0.8 | 0.17992 | 2.3 | 27.5 |
| COX2. | DFX vs Saline | 0.04643 | 5.83 | (1, 7) | 43.2 | 0.44543 | 0.65 | 4.8 |
| FSP1. | DFX vs Saline | 0.02537 | 8.01 | (1, 7) | 52.9 | 0.73515 | 0.12 | 0.8 |

**H)** IVH increases the expression of HMOX1, LPCAT3, COX2, and sEH in periventricular germinal matrix and white matter of human premature infants (Western blot analyses, Fig. 8)

**Germinal matrix**

| **Para-**  **meter** | **Comparison** | **P_value**  **Group** | **F_value**  **Group** | **DF**  **_Group** | **Var%_**  **Group** | **P_value**  **Sex** | **F value**  **Sex** | **DF_**  **Sex** | **Var%_**  **Sex** |
| --- | --- | --- | --- | --- | --- | --- | --- | --- | --- |
| 15LOX | no IVH vs. IVH | 0.774009 | 0.09 | (1, 7) | 1 | 0.202378 | 1.98 | (1, 7) | 21.8 |
| COX2. | no IVH vs. IVH | 0.524586 | 0.45 | (1, 7) | 6 | 0.92512 | 0.01 | (1, 7) | 0.1 |
| eSH. | no IVH vs. IVH | 0.094945 | 3.72 | (1, 7) | 31 | 0.293141 | 1.29 | (1, 7) | 10.7 |
| HMOX1. | no IVH vs. IVH | 0.043708 | 6.03 | (1, 7) | 46.2 | 0.900515 | 0.02 | (1, 7) | 0.1 |
| FTH1. | no IVH vs. IVH | 0.063608 | 4.85 | (1, 7) | 40.1 | 0.645919 | 0.23 | (1, 7) | 1.9 |
| P53. | no IVH vs. IVH | 0.263426 | 1.48 | (1, 7) | 17.4 | 0.848556 | 0.04 | (1, 7) | 0.5 |

**White matter**

| 15LOX | no IVH vs. IVH | 0.741529 | 0.12 | (1, 7) | 1.6 | 0.79127 | 0.08 | (1, 7) | 1.1 |
| --- | --- | --- | --- | --- | --- | --- | --- | --- | --- |
| COX2. | no IVH vs. IVH | 0.007981 | 15.21 | (1, 6) | 71.2 | 0.71879 | 0.14 | (1, 6) | 0.7 |
| eSH. | no IVH vs. IVH | 0.043979 | 6.46 | (1, 6) | 51.3 | 0.739907 | 0.12 | (1, 6) | 1 |
| HMOX1. | no IVH vs. IVH | 0.056589 | 5.2 | (1, 7) | 39.9 | 0.395309 | 0.82 | (1, 7) | 6.3 |
| FTH1. | no IVH vs. IVH | 0.414224 | 0.75 | (1, 7) | 7.8 | 0.216061 | 1.85 | (1, 7) | 19.3 |
| P53. | no IVH vs. IVH | 0.006893 | 14.29 | (1, 7) | 64.8 | 0.410557 | 0.77 | (1, 7) | 3.5 |

**Table S3: Effect of sex as a biologic variable by employing ANCOVA (data from Fig. S1-9).**

1. IVH induces oxidative stress (Fig. S1)

| **Para**  **meter** | **Comparison** | **P_value**  **Group** | **F_value**  **Group** | **DF_**  **Group** | **Var%_**  **Group** | **P_value**  **Sex** | **F value**  **Sex** | **Var%_**  **Sex** |
| --- | --- | --- | --- | --- | --- | --- | --- | --- |
| Isoprostane | IVH vs. no IVH | 0.922726 | 0.01 | (1, 7) | 0.1 | 0.765005 | 0.1 | 1.4 |
| Isofuran | IVH vs. no IVH | 0.333369 | 1.08 | (1, 7) | 12.6 | 0.521245 | 0.46 | 5.3 |
| GSH/GSS | IVH vs. no IVH | 0.013981 | 10.59 | (1, 7) | 51.5 | 0.129038 | 2.96 | 14.4 |

1. Effect of IVH on enzymes affecting iron, phospholipid, and oxidative stress (Western blot analyses, Fig S7A-C**)**

| Para-meter | Com  parison | P_value  Group | F_  Group | DF_  Group | Var%_  Group | P_value  Sex | F_  Sex | Var%  _Sex |
| --- | --- | --- | --- | --- | --- | --- | --- | --- |
| HO1 | IVH vs. no IVH | 0.0006948 | 33.12 | (1, 7) | 79.2 | 0.2307556 | 1.72 | 4.1 |
| FTH1 | IVH vs. no IVH | 0.0003007 | 43.73 | (1, 7) | 83.4 | 0.2292458 | 1.74 | 3.3 |
| DMT1 | IVH vs. no IVH | 0.010015 | 12.24 | (1, 7) | 63.6 | 0.90865 | 0.01 | 0.1 |
| 5LOX | IVH vs. no IVH | 0.000329 | 42.43 | (1, 7) | 83.3 | 0.26244 | 1.49 | 2.9 |
| 12LOX | IVH vs. no IVH | 0.02450 | 8.15 | (1, 7) | 51 | 0.392814 | 0.83 | 5.2 |
| 15LOX | IVH vs. no IVH | 0.706332 | 0.15 | (1, 7) | 1.9 | 0.38983 | 0.84 | 10. |
| COX2 | IVH vs. no IVH | 0.175606 | 2.27 | (1, 7) | 22.9 | 0.45355 | 0.63 | 6.4 |
| LPCAT3 | IVH vs. no IVH | 0.130121 | 2.94 | (1, 7) | 26 | 0.27703 | 1.39 | 12.3 |
| ASCL4 | IVH vs. no IVH | 0.008767 | 12.94 | (1, 7) | 58.1 | 0.17166 | 2.32 | 10.4 |
| P53 | IVH vs. no IVH | 0.040505 | 6.29 | (1, 7) | 45.1 | 0.44299 | 0.66 | 4.7 |
| SLC7A1 | IVH vs. no IVH | 0.480020 | 0.56 | (1, 7) | 7.2 | 0.69852 | 0.16 | 2.1 |
| GPX1 | IVH vs. no IVH | 0.108741 | 3.38 | (1, 7) | 31.1 | 0.50647 | 0.49 | 4.5 |
| GPX4 | IVH vs. no IVH | 0.589436 | 0.32 | (1, 7) | 4.3 | 0.72051 | 0.14 | 1.9 |
| NRF2 | IVH vs. no IVH | 0.080973 | 4.15 | (1, 7) | 33.3 | 0.29027 | 1.31 | 10.5 |
| KEAP1 | IVH vs. no IVH | 0.049314 | 5.64 | (1, 7) | 44.3 | 0.79125 | 0.0 | 0.6 |

C. Effect of IVH on key molecules playing a role in oxidative stress and ferroptosis at D3 (Fig. S8A, Western blot)

| **Parameter** | **Comparison** | **P_Group** | **F_Group** | **DF_Group** | **Var%_Group** | **P_Sex** | **F_Sex** | **Var%_Sex** |
| --- | --- | --- | --- | --- | --- | --- | --- | --- |
| SLC7A11. | IVH vs. no IVH | 0.062639 | 4.89 | (1, 7) | 40.9 | 0.808868 | 0.06 | 0.5 |
| GPX1. | IVH vs. no IVH | 0.180717 | 2.29 | (1, 6) | 19.9 | 0.12257 | 3.23 | 28 |
| GPX4. | IVH vs. no IVH | 0.239618 | 1.65 | (1, 7) | 18.1 | 0.512428 | 0.48 | 5.2 |
| DMT1. | IVH vs. no IVH | 0.123948 | 3.06 | (1, 7) | 29.1 | 0.518432 | 0.46 | 4.4 |
| P53. | IVH vs. no IVH | 0.004009 | 17.68 | (1, 7) | 54 | 0.024965 | 8.08 | 24.7 |
| MBOAT1. | IVH vs. no IVH | 0.004648 | 16.7 | (1, 7) | 57.6 | 0.054617 | 5.31 | 18.3 |
| MBOAT2. | IVH vs. no IVH | 0.012321 | 11.19 | (1, 7) | 47.6 | 0.054289 | 5.33 | 22.7 |

D. Effect of DFX on key molecules playing a role in oxidative stress and ferroptosis at D3 (Fig. S8C, western blot)

| **Parameter** | **Comparison** | **P value**  **Group** | **F value**  **Group** | **DF_Group** | **Var%Group** | **P value**  **Sex** | **F value Sex** | **Var%Sex** |
| --- | --- | --- | --- | --- | --- | --- | --- | --- |
| ferropotin | DFX vs. Saline | 0.06562 | 4.75 | (1, 7) | -0.85 | 0.27553 | 1.4 | 10.6 |
| ASCL4. | DFX vs. Saline | 0.761033 | 0.1 | (1, 7) | -0.44 | 0.60068 | 0.3 | 4.1 |
| SLC7A11. | DFX vs. Saline | 0.52634 | 0.44 | (1, 7) | -0.79 | 0.34095 | 1.04 | 12.3 |
| GPX1. | DFX vs. Saline | 0.43876 | 0.67 | (1, 7) | -0.59 | 0.24282 | 1.63 | 17.5 |
| GPX4. | DFX vs. Saline | 0.98612 | 0 | (1, 7) | -0.82 | 0.15231 | 2.58 | 26.9 |
| NRF2. | DFX vs. Saline | 0.292514 | 1.3 | (1, 7) | -0.18 | 0.45450 | 0.63 | 7 |
| MBOAT1. | DFX vs. Saline | 0.177209 | 2.25 | (1, 7) | -0.46 | 0.03773 | 6.54 | 41.4 |
| MBOAT2. | DFX vs. Saline | 0.68180 | 0.18 | (1, 7) | -0.08 | 0.18118 | 2.2 | 23.5 |

E. Effect of IVH on key molecules playing a role in oxidative stress and ferroptosis at D3 (Fig. S8B, S8D, RT-qPCR)

| **Para-**  **meter** | **Comparison** | **P_value**  **Group** | **F_value**  **Group** | **DF_**  **Group** | **Var%_**  **Group** | **P_**  **Sex** | **F_**  **Sex** | **Var%_**  **Sex** |
| --- | --- | --- | --- | --- | --- | --- | --- | --- |
| SLC7A11. | IVH vs. no IVH | 0.243111 | 1.620 | (1, 7) | 18.5 | 0.704985 | 0.16 | 1.8 |
| NRF2. | IVH vs. no IVH | 0.254384 | 1.540 | (1, 7) | 18 | 0.907263 | 0.01 | 0.2 |
| LPCAT3. | IVH vs. no IVH | 0.61044 | 0.280 | (1, 7) | 3.8 | 0.645104 | 0.23 | 3.1 |
| HMOX1. | IVH vs. no IVH | 0.092105 | 3.8 | (1, 7) | 34.6 | 0.682364 | 0.18 | 1.7 |
| GPX1. | IVH vs. no IVH | 0.377771 | 0.89 | (1, 7) | 9 | 0.199054 | 2.01 | 20.3 |
| DMT1. | IVH vs. no IVH | 0.377771 | 0.89 | (1, 7) | 9 | 0.199054 | 2.01 | 20.3 |
| 15LOX | IVH vs. no IVH | 0.178754 | 2.23 | (1, 7) | 23.4 | 0.589581 | 0.32 | 3.3 |
| NRF2. | DFX vs. saline | 0.868534 | 0.03 | (1, 7) | 0.4 | 0.508663 | 0.48 | 6.5 |
| HMOX1. | DFX vs. saline | 0.900087 | 0.02 | (1, 7) | 0.2 | 0.759128 | 0.1 | 1.4 |
| SLC711A1. | DFX vs. saline | 0.790801 | 0.08 | (1, 7) | 1 | 0.698364 | 0.16 | 2.3 |
| LPCAT3. | DFX vs. saline | 0.347148 | 1.04 | (1, 6) | 13.9 | 0.523159 | 0.46 | 6.1 |
| GPX1. | DFX vs. saline | 0.159699 | 2.47 | (1, 7) | 21.4 | 0.191701 | 2.09 | 18.1 |
| DMT1. | DFX vs. saline | 0.016849 | 9.73 | (1, 7) | 36.5 | 0.016228 | 9.9 | 37.2 |
| CD71. | DFX vs. saline | 0.812135 | 0.06 | (1, 6) | 1 | 0.709083 | 0.15 | 2.5 |
| 15LOX | DFX vs. saline | 0.066456 | 4.72 | (1, 7) | 33.6 | 0.171187 | 2.32 | 16.6 |

F. Effect of DFX treatment on enzymes at D7 and D14 (Fig. S9A-D).

| **Para**  **meter** | **Comp-**  **arison** | **P_value**  **Group** | **F_value**  **Group** | **DF_**  **Group** | **Var%_**  **Group** | **P_value**  **Sex** | **F_value**  **Sex** | **Var%_**  **Sex** |
| --- | --- | --- | --- | --- | --- | --- | --- | --- |
| **D7** |  |  |  |  |  |  |  |  |
| HMOX1 | DFX vs.Saline | 0.82261 | 0.05 | (1, 7) | 0.6 | 0.14523254 | 2.69 | 27.6 |
| FTH1 | DFX vs.Saline | 0.39225 | 0.83 | (1, 7) | 10.5 | 0.75485624 | 0.11 | 1.3 |
| FTL | DFX vs.Saline | 0.06474 | 4.79 | (1, 7) | 35 | 0.21130894 | 1.89 | 13.8 |
| DMT1. | DFX vs.Saline | 0.00061 | 34.45 | (1, 7) | 80.6 | 0.29114616 | 1.3 | 3 |
| 5LOX | DFX vs.Saline | 0.19140 | 2.09 | (1, 7) | 22.4 | 0.63663987 | 0.24 | 2.6 |
| 15LOX | DFX vs.Saline | 0.27082 | 1.43 | (1, 7) | 14.6 | 0.27850116 | 1.38 | 14.1 |
| COX. | DFX vs.Saline | 0.73813 | 0.12 | (1, 7) | 1.6 | 0.43744476 | 0.68 | 8.7 |
| LPCAT3 | DFX vs.Saline | 0.06187 | 4.93 | (1, 7) | 40.9 | 0.74058433 | 0.12 | 1 |
| ASCL4 | DFX vs.Saline | 0.14050 | 2.76 | (1, 7) | 27.9 | 0723914423 | 0.14 | 1.4 |
| p53. | DFX vs.Saline | 0.02431 | 8.18 | (1, 7) | 53.9 | 0.98746770 | 0 | 0 |
| caspase-3 | DFX vs.Saline | 0.02597 | 7.92 | (1, 7) | 53 | 0.87744695 | 0.03 | 0.2 |
| GPX1. | DFX vs.Saline | 0.02676 | 7.8 | (1, 7) | 46.1 | 0.18830384 | 2.12 | 12.5 |
| GPX4 | DFX vs.Saline | 0.48787 | 0.54 | (1, 7) | 6.9 | 0.68488071 | 0.18 | 2.3 |
| SLC7A11 | DFX vs.Saline | 0.99295 | 0 | (1, 7) | 0 | 0.76885094 | 0.09 | 1.3 |
| KEAP1. | DFX vs.Saline | 0.92757 | 0.01 | (1, 7) | 0.1 | 0.56947888 | 0.36 | 4.8 |
| NRF2. | DFX vs.Saline | 0.00183 | 23.61 | (1, 7) | 71.7 | 0.17087846 | 2.33 | 7.1 |
| **D14** |  |  |  |  |  |  |  |  |
| HMOX1 | DFX vs.Saline | 0.00450 | 16.91 | (1, 7) | 70.2 | 0.69200491 | 0.17 | 0.7 |
| FTH1. | DFX vs.Saline | 0.01531 | 10.16 | (1, 7) | 58.9 | 0.76038447 | 0.1 | 0.6 |
| 15LOX | DFX vs.Saline | 0.01555 | 10.09 | (1, 7) | 58 | 0.60080850 | 0.3 | 1.7 |
| ASCL4. | DFX vs.Saline | 0.001451 | 25.68 | (1, 7) | 78.6 | 0.97645014 | 0 | 0 |

**Supplementary Methods**

**Immunohistochemistry (IHC):** Immunohistochemical staining was performed as described previously ^1^. The primary antibodies employed in experiments included **m**ouse monoclonal TfR1 (catalog #MAB1765, EMD Millipore), rabbit cleaved caspase-3 (catalog # 9664, Cell signaling Inc. ), rat polyclonal 4HNE (catalog# AB46545 Abcam), goat Iba1(catalog# ab5076, Abcam), rat monoclonal CD11B (Thermofisher), mouse monoclonal myelin basic protein (catalog# ab62631), mouse monoclonal GFAP (Catalog # AB5804, Sigma), soluble epoxy hydrolase (catalog# SC-166961, Santa Cruz Biotech), mouse monoclonal Cox2 (catalog# SC-19999, Santa Cruz Biotech), mouse monoclonal LPCAT3 (catalog# 67882-1-Ig, Proteintech), rabbit polyclonal ( catalog# 10701-1-AP, Proteintech). Briefly, we hydrated the 4% paraformaldehyde-fixed sections from rabbit kits or human infant brain in 0.1M PBS, blocked the sections with normal donkey serum in PBS with 0.01% Triton-X (PBST), and then incubated them with the primary antibodies diluted in PBS at 4°C overnight. After several washes in PBS, the sections were incubated with secondary antibody diluted in 2% normal donkey serum in PBS at room temperature for 60 minutes. Finally, after washing in PBS a few times, the sections were mounted with Slow Fade Light Antifade reagent (Molecular Probes, Invitrogen, CA) and were visualized under a confocal microscope (Nikon Instruments, Japan). Stereological quantification of cells was performed using a fluorescent microscope (Axioskop 2 plus, Carl Zeiss Inc) with motorized specimen stage for automated sampling (ASI, Eugene, OR), CCD color video camera (Microfire; Optronics, Goleta, CA), and stereology software (Stereologer, SRC, Baltimore, MD).

**Stereological quantification of myelination, astrogliosis, and microglia.** We evaluated many stereological parameters using a computerized software system (*Stereologer,* Stereology Resource Center, Chester, MD). Briefly, coronal sections of 30 μm thickness were cut on a cryostat with a section sampling interval of 120 μm, to achieve 5-6 sections at the level of mid-septal nucleus. The sections were double-labeled with myelin basic protein (MBP)-specific antibody and DAPI (nuclear stain) and the myelination was quantified as follows. The reference spaces (corona radiata and corpus callosum) were outlined on the coronal section using a 5x objective. The volume of the outlined area was assessed using a point counting probe (frame 25 μm x 25 μm; guard zone 2 μm, inter-frame interval = 300 μm). The total volume fraction (myelin load) of myelin stained by MBP antibody through a defined reference space in corpus callosum and corona radiata was measured using the object area fraction probe using a 60x oil lens. For the area fraction probe (frame 25 μm x 25 μm; guard zone 2 μm, interframe interval 300 μm), the investigator clicked on the grid points that overlapped the myelin fibers in sections labeled with MBP. The area fraction of myelination was measured as the ratio of product of the area per point and number of points hitting reference area the over the product of the area per point and number of points hitting the sampled area [a(point)• ∑P_samp_], as reported previously. A coefficient of error (CE) < 0.10 was considered acceptable. To assess gliosis, we quantified total volume fraction of astrocyte cell body and glial fibers in a similar manner as for myelin. For microglia count (labeled against Iba1 and CD11B), optical dissector method (frame, 25 X 25 µm; guard zone, 2 µm, interframe interval, 280 µm) was employed.

**Quantification of ferroptosis and TUNEL^+^ cells**: To assess ferroptosis, we double-labeled brain sections with TfR1 (transferrin receptor 1) and 4HNE antibodies. To evaluate apoptosis, we performed TUNEL (Fluorescent in situ detection of DNA fragmentation) and DAPI staining. We quantified TUNEL^+^ and TfR1^+^ cells in periventricular regions (dorsal and medial) using a confocal microscope (Nikon). Four coronal sections were obtained at the level of the mid-septal nucleus (five 20 μm sections collected at 100 μm intervals). A blind investigator performed quantification in a random and unbiased fashion. Cells were counted in ~25 images (5 images x 4-5 sections) for each brain region in every kit (n=5 kits per group).

**Iron staining (Prussian Blue staining):** The frozen sections from rabbit kits or human brain sections were immersed in a freshly prepared hydrochloric acid-potassium ferrocyanide working solution for 10 minutes. After rinsing the sections in distilled water, they were counterstained with a Nuclear Fast Red solution for 5 minutes. After another wash in tap water, the section was rehydrated in alcohol, treated with xylene, and mounted in the resinous medium.

**Western blot analyses:** We homogenized the frozen, dissected tissue from the MGE and cerebral cortex in a lysis buffer (3% SDS, 10% glycerol, and 62.5 mM Tris-HCl) using a mechanical homogenizer, and then sonicated the lysate before centrifugation. The supernatant protein concentration was determined using a BCA protein assay kit (Pierce Kit #23227, Thermo Scientific, Rockford, IL), and dilutions of BSA were employed to create a standard curve. After boiling the samples in Laemmli buffer (catalog #161-0737, Bio-Rad, CA), total protein samples were separated by SDS-PAGE. Equal amounts of protein (10-20 µg) were loaded onto 4-15% or 4-20% gradient precast gels (Bio-Rad, CA), based on the molecular weight of the target protein. Separated proteins were transferred onto a polyvinylidene difluoride (PVDF) membrane by electrotransfer. Membranes were then incubated overnight with primary antibodies. We detected target proteins with the chemiluminescence ECL system (GE Healthcare) by using secondary antibodies conjugated with horseradish peroxidase (Jackson Immuno-Research, West Grove, PA). As described previously ^1^, the blots from each experiment were densitometrically analyzed using Image J, and optical density (OD) values for each protein of interest were normalized to β-actin. The antibodies used for Western blot analyses were Rabbit cleaved caspase-3 (Cell signaling, catalog # 9604), rabbit polyclonal heme oxygenase 1 (Proteintech, catalog # 10701-1-AP), rabbit polyclonal ferritin heavy chain 1 ( Cell Signaling Inc., catalog # 4393), rabbit polyclonal DMT1/SLC11A2 (Cell signaling Inc., catalog # 150830, mouse monoclonal 𝛽-actin (Sigma, catalog # A5316), mouse monoclonal 5-LO (Santa Cruz Biotech, catalog # SC-136195), mouse monoclonal 12-LO (Santa Cruz Biotech, catalog # SC-365194), mouse monoclonal 15-LO (Santa Cruz Biotech, catalog # SC133085), mouse monoclonal COX2 (BD Biosciences, catalog #610204), mouse monoclonal LPCAT3 (Proteintech, catalog # 67882-1-Ig), mouse monoclonal ASCL4 (Santa Cruz Biotech, catalog #SC365230), mouse monoclonal P53 (Santa Cruz Biotech, catalog #SC-263), rabbit polyclonal XCT/SLC7A11 (Cell signaling Inc, catalog # 12691), ), rabbit polyclonal GPX1 (Cell signaling Inc, catalog #3286) ), rabbit polyclonal GPX4 (Cell Signaling Inc. Catalog # 52455), rabbit polyclonal KEAP1 (Cell signaling Inc. Catalog # 8047), Rabbit polyclonal NRF2 (Cell Signaling Inc. Catalog # 12721), rabbit polyclonal HIF1𝛼 (Novus Biologicals, catalog # NB100-654), mouse monoclonal myelin basic protein (Abcam, catalog #Ab62631), mouse monoclonal myelin associated glycoprotein (Abcam, catalog # ab89780), mouse monoclonal CNPase (Sigma, Catalog # C5922), rabbit monoclonal GFAP (Sigma, catalog #AB5804), mouse monoclonal CYP4A (Santa Cruz Biotech, catalog # SC-271983), rabbit monoclonal CYP2C8/9/18/19 (Proteintech, 16546-1-AP), She (Santa Cruz Biotech, catalog #SC166961), mouse monoclonal ASCL4 (Santa Cruz Biotech, catalog #SC365230), rabbit polyclonal KEAP-1 (Cell Signaling Inc. Catalog # 8047).

**Quantitative Real-Time Polymerase Chain Reaction (qRT-PCR)**: As described previously, gene expression was quantified by real-time PCR ^2^. Briefly, total RNA was isolated using an RNeasy Mini kit (catalog #74104, Qiagen) from coronal brain slices taken at the level of the midseptal nucleus. cDNA was synthesized using Superscript II RT enzyme (catalog # 05081955001, Roche, Indianapolis, IN) and a real-time quantitation using an ABI QuantStudio^TM^ real-time PCR system (ThermoFisher Sci.). TaqMan probes were bought from Life Technologies. Their assay IDs were as follows: GAPDH (Oc03823402_g1), NRF2L2 (OC06782280_m1), GPX1 (OC04096645_g1), SLC7A11(OC06683691_m1), 15-LO (ALOX15, OC03823548_s1), HO1 (OC03396031_m1), LPCAT3 (OC06732656_g1), CD71 (OC06680198_m1), DMT1(slc11a2, OC06775845_m1), and rabbit SRY (NM_001171148.1)

**Dissection of MGE, RNA seq, and analyses**: Immediately after sacrificing the animal, a 2-3 mm coronal slice taken at the level of the head of the caudate nucleus was placed under a Zeiss Stereo (Discovery V12) microscope**,** which is equipped with MC1500 (Schott) illumination. For glycerol-treated kits with IVH and glycerol-treated kits without IVH (control), samples were run in duplicate (n=2 each). The lateral margin of the ventricle was dissected with a stabbing knife (Sharpoint). The dissected samples were immersed in chilled PBS buffer and dissociated employing a Neural Tissue Dissociation Kit (Catalog #130-107- 628, Miltenyi Biotec, CA, USA), according to the manufacturer’s instructions. Cell debris and red blood cells were removed by Debris Removal Solution (Miltenyi Biotec, CA, USA) and Red Blood Cell Lysis Solution (Miltenyi Biotec, CA, USA). Einstein Genomics Core processed the isolated cells to prepare a single-cell RNA seq library. Single Cell 3’ gene expression libraries consisted of standard Illumina paired-end constructs, which commence with P5 and end with P7. 16 bp 10x Barcodes were encoded at the beginning of TruSeq Read 1, while ten bp i5 and i7 sample index sequences were incorporated as sample index reads. TruSeq Read 1 and Read 2 were the standard Illumina sequencing primer sites used in paired-end sequencing. Sequencing libraries were assessed for quality on the Agilent TapeStation (Agilent Technologies, Palo Alto, CA, USA), and quantified by using Qubit 2.0 Fluorometer (Invitrogen, CA**)**. Pooled libraries were quantified using qPCR (Applied Biosystems, Carlsbad, CA, USA) before loading into the Illumina sequencing platform. The samples were sequenced at a configuration compatible with the recommended guidelines outlined by 10X Genomics. Raw sequence data (.bcl files) produced from the Illumina Novaseq 6000 were changed into fastq files and de-multiplexed using the 10X Genomics’ cell ranger fast command. Fastq files of each rabbit sample were aligned against mm10 genome v1.2.0 and converted to count matrices using Cell Ranger software v3.0.2. A unique molecular identifier (UMI) was identified to eliminate PCR duplicates. Quality control and downstream analysis were performed in R v4.0.2, using Seurat package v3.2.2 (Butler et al., 2018; Stuart et al.,2019). Cells with a gene count less than 200 or more than 5000 or with a mitochondrial gene ratio greater than 20% were filtered out. Differential gene expression analysis was performed to compare samples with different conditions using the Seurat FindMarkers function. Genes having greater than 1.2-fold change and adjusted p-value of < 0.01 were counted as differentially expressed. Gene set enrichment analysis (GSEA) was done using fgsea R package v1.14.0 against MSigDB v5 KEGG pathway database. Gene sets with p-values less than 0.05 were considered significant.

**Neurobehavioral assessment***:* Kits with IVH treated with DFX or vehicle underwent neurobehavioral assessments at D21 as before ^2^. Any-Maze Software (Stoelting Company, Wood Dale, IL) was used to record and analyze all behavioral assessments by tracking movement and time spent in delineated zones by an overhead video camera*.* We performed an open Field Test using a square arena of 40 × 40 × 30 inches and recorded a range of parameters such as distance traveled, speed, activity levels, gross locomotor activity, and exploration habits. Distance moved, velocity, and time spent in delineated zones were computed.

**Mass Spectrometric Lipidomic and Oxylipin Studies:** Lipidomic and eicosanoid assays were performed as described previously.^3,4^ In brief, brain tissue samples (approximately 30 mg) were used for each analysis of eicosanoids and lipidomics. About ten ceramic beads (1.0 mm dia., BioSpec) were added to each sample. A volume of 9 times the extraction solvent (Dulbecco’s Phosphate Buffered Saline for eicosanoids analysis and ethanol containing internal standards for lipidomics analysis) was added to the samples to the weight of the tissue. After four cycles of 30 seconds of bead beating (with a 1-minute interval), the samples were centrifuged at 12000 rpm at 4 °C for 10 minutes. For eicosanoid analysis, the supernatant was taken out and diluted to 1 mL with PBS. A mixture of 100 μL of stable isotope-labeled internal standards was spiked to the samples. The extracted eicosanoids were performed with Strata-X reversed-phase SPE columns (Phenomenex). Eicosanoids were eluted with methanol. The samples were dried under a gentle nitrogen flow. The dried samples were reconstituted in 100 μL of methanol and then pending LC injection. For lipidomics analysis, the supernatant was transferred to a sampling vial and dried under gentle nitrogen flow. The dried samples were reconstituted in 100 μL of ethanol and were pending LC injection. The samples were analyzed with AB Sciex 6500+ coupled with Waters Acquity UPLC. Scheduled Multiple Reaction Monitoring (MRM) mode was used for both eicosanoids and lipidomics analysis. Eicosanoids separation was performed with a BEH shield RP18 column (Waters), while lipids separation was performed with a CSH Fluoro-Phenyl column (Waters). A pooled quality control (QC) sample was added to the sample list. This QC sample was injected six times for coefficient variation (CV) calculation for data quality control .. Eicosanoids/lipids with CVs higher than 30% will be excluded from further statistical analysis. Data analysis was performed with MultiQuant software (AB Sciex). Standards used in the experiment were obtained from Cayman Chemicals.

**Measurement of Isoprostanes, isofurans, and glutathione:** Isoprostanes and isofurans were measured by the Neurochemistry Core Laboratory at Vanderbilt University Medical Center in Nashville, TN, under the supervision of Dr. Ginger Milne, as previously described in her articles.^5,6^ Briefly, the tissue sample is homogenized in ice-cold Folch solution. The sample tube was then flushed with a stream of nitrogen or argon for 30 to 60 seconds to remove air. After centrifugation, the top aqueous layer was carefully pipetted off and discarded. The organic layer was evaporated under a stream of nitrogen until dry. The lipids were resuspended in 1 mL of methanol containing 0.005% BHT, and then 1 mL of a 15% (wt/vol) aqueous KOH solution is added. The mixture was incubated at 37°C for 20 minutes. Following incubation, the mixture was acidified to pH 3 using 1N HCl, diluted to 0.01N HCl, and 1 ng of the internal standard [2H4]-15-F2t-IsoP is added. Chromatography/Negative Ion Chemical Ionization Mass Spectrometry (C/NICI-MS) was performed using an Agilent 5973 Inert Mass Selective Detector coupled with an Agilent 6890N Network GC system (Agilent Labs, Torrance, CA) that is interfaced with an Agilent computer. Gas chromatography (GC) was conducted using a 15 m, 0.25 µm film thickness, DB-1701 fused silica capillary column (J and W Scientific, Folsom, CA). The column temperature is programmed to increase from 190°C to 300°C at a rate of 20°C per minute.

  Total and oxidized Glutathione were measured by Bioassay System Service, Hayward, CA under supervision of Dr. Michael McDowell using EnzyChrom GSH/GSSG assay kit (EZTT 100 assay kit, Bioassay System, Hayward, CA) based on instructions in the manual. Briefly, the samples were homogenized in the extraction buffer. The supernatant was mixed with the MPA reagent, and the optical density was measured at 412 nm.

**Measurement of iron using inductively coupled plasma mass spectrometry (ICP-MS):** The analysis was conducted at the University at Buffalo Chemistry Instrument Center (UBCIC) at the University at Buffalo, SUNY.  Sample preparation consisted of solubilizing 100 mg of the brain slice taken at the level of the mid-septal septal nucleus into 65% nitric acid and incubating for 8 hours at 70°C. The samples were then diluted to 5% in deionized water. Iron sample analyses were performed using a Thermo Scientific XSERIES 2 Inductively Coupled Plasma Mass Spectrometer (ICP-MS). The sample introduction system consisted of a Burgener Ari Mist Nebulizer, a peltier-cooled (4°C) double pass glass spray chamber, a quartz torch, and a nickel sampler and skimmer cone. Sample transport from the ESI SC2 DX autosampler to the nebulizer was performed using a peristaltic pump. The instrument was operated in kinetic energy dissociation (KED) mode to minimize interference using a gas mixture of 7% hydrogen in helium for the cell gas.  Data were acquired and processed using the Thermo Plasma Lab software via internal standardization with scandium and indium.

**Neutral lipid staining:** Iba1-labeled sections were treated with 20 µg/ml of BODIPY 493/503 (4,4-Difluoro-1,3,5,7,8-Pentamethyl-4-Bora-3a,4a-Diaza-*s*-Indacene) diluted in 1x PBS for one hour. The brain section was washed in PBS and mounted with Slow Fade Light Antifade reagent (Molecular Probes, Invitrogen, CA), and was visualized under a confocal microscope (Nikon Instruments, Japan).

**Supplementary References**

1. Ballabh, P.*, et al.* Angiogenic inhibition reduces germinal matrix hemorrhage. *Nat Med* **13**, 477-485 (2007).

2. Krishna, S.*, et al.* PPAR-gamma activation enhances myelination and neurological recovery in premature rabbits with intraventricular hemorrhage. *Proc Natl Acad Sci U S A* **118**(2021).

3. Jao, J.*, et al.* Distinct Lipidomic Signatures in People Living With HIV: Combined Analysis of ACTG 5260s and MACS/WIHS. *J Clin Endocrinol Metab* **107**, 119-135 (2022).

4. Jao, J.*, et al.* Distinct cord blood C-peptide, adipokine, and lipidomic signatures by in utero HIV exposure. *Pediatr Res* **92**, 233-241 (2022).

5. Milne, G.L., Gao, B., Terry, E.S., Zackert, W.E. & Sanchez, S.C. Measurement of F2- isoprostanes and isofurans using gas chromatography-mass spectrometry. *Free Radic Biol Med* **59**, 36-44 (2013).

6. Milne, G.L., Sanchez, S.C., Musiek, E.S. & Morrow, J.D. Quantification of F2-isoprostanes as a biomarker of oxidative stress. *Nat Protoc* **2**, 221-226 (2007).
