# Supplementary material for: Iron chelation targets lipid metabolism to reduce white matter injury in germinal matrix hemorrhage
Source: Cell Death Dis. 2026 May 23;17(1):648. doi: 10.1038/s41419-026-08866-z (PMC13376222; doi:10.1038/s41419-026-08866-z)
Supplement: Supplementary file 3 — Uncropped Western blots [file 41419_2026_8866_MOESM3_ESM.docx]

**Fig. 4C**

**1. MBP**

**
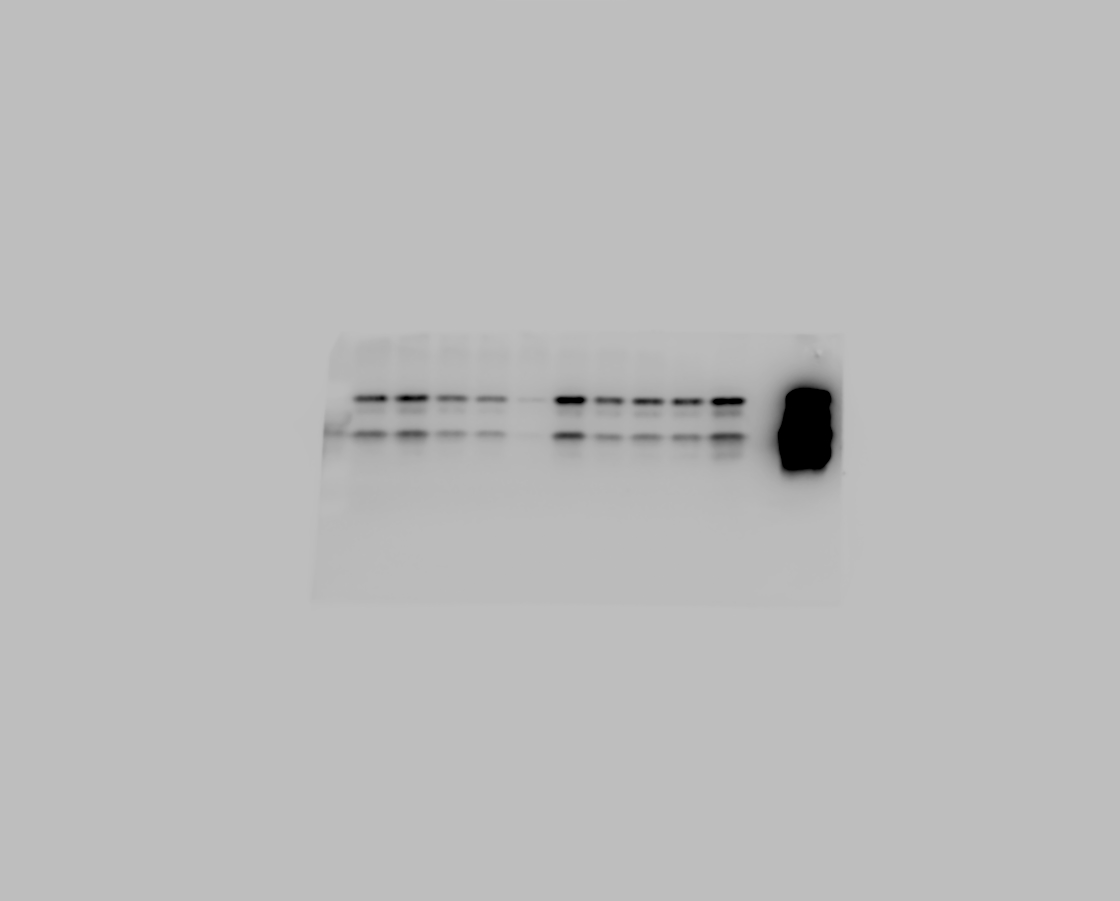
**

23kD

**2.MAG**

**
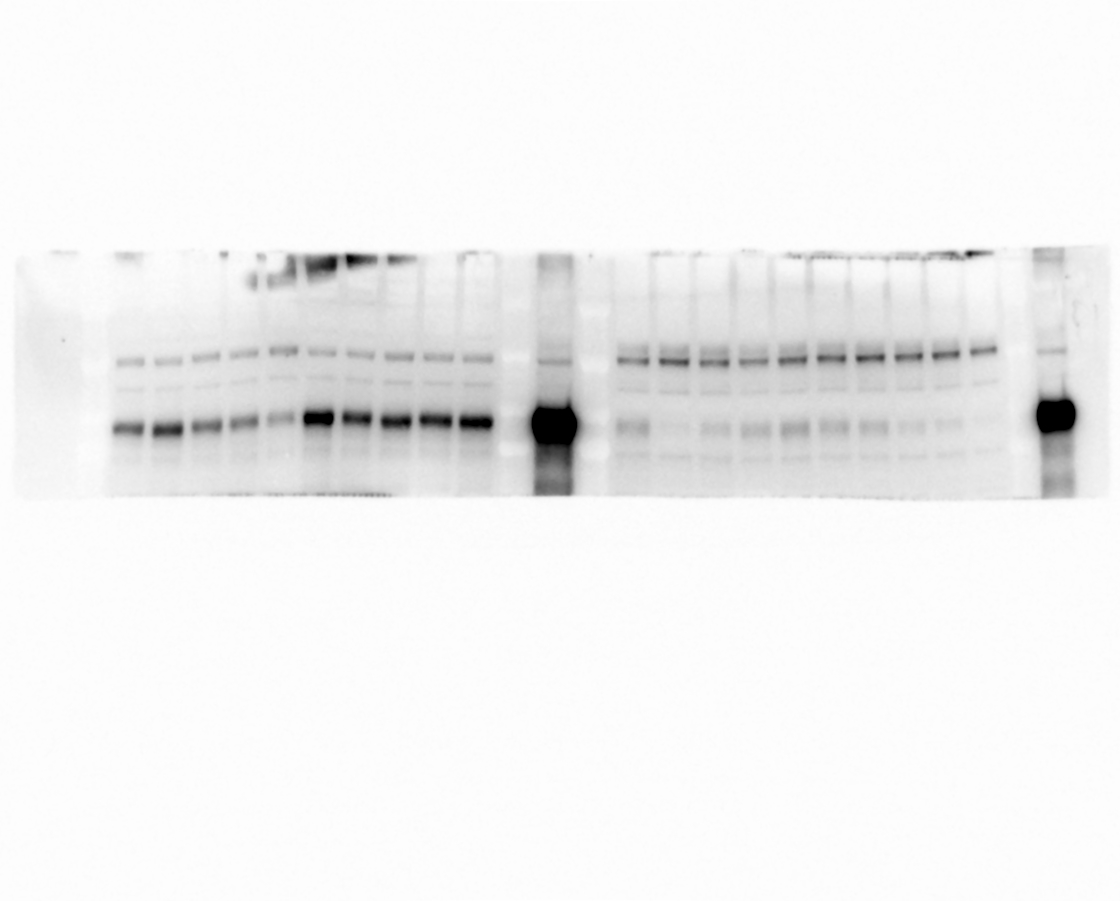
**

67-72kD

**3. Beta Actin**

**
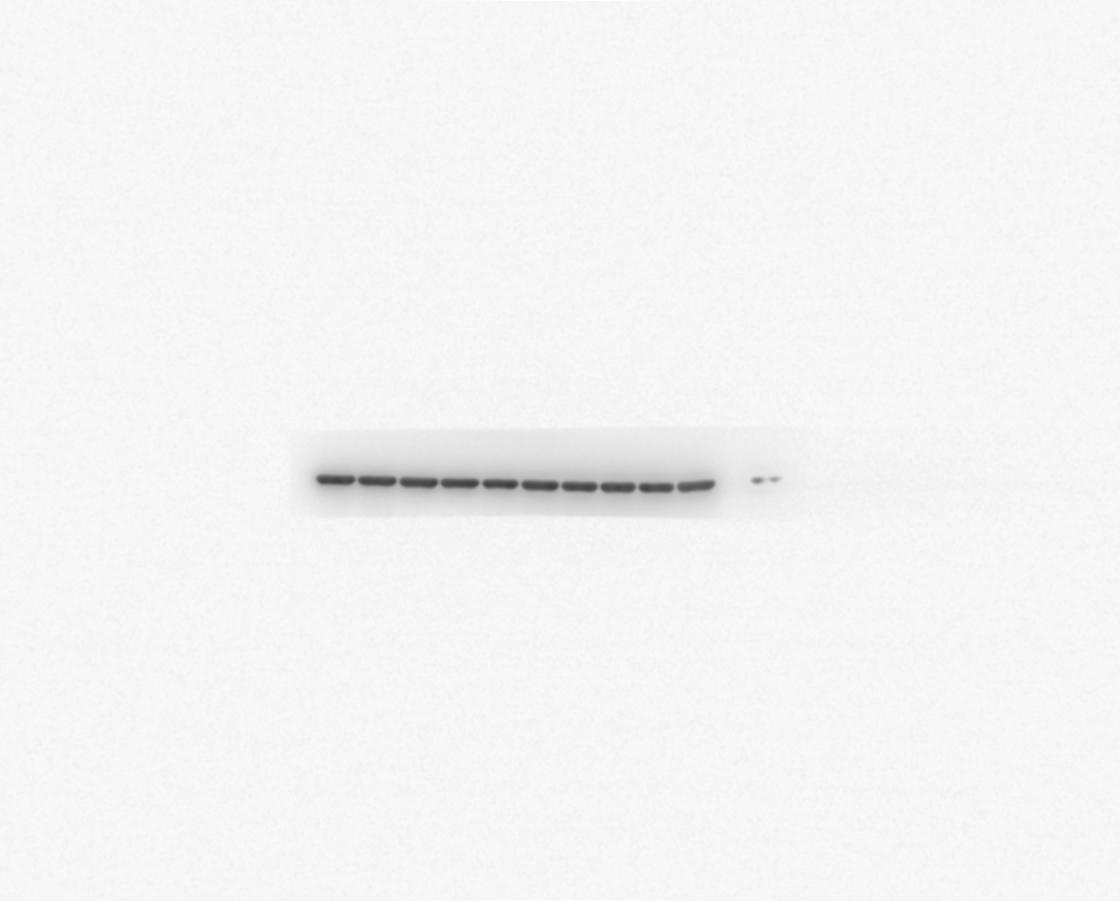
**

42kD

**4.CNPase**

**
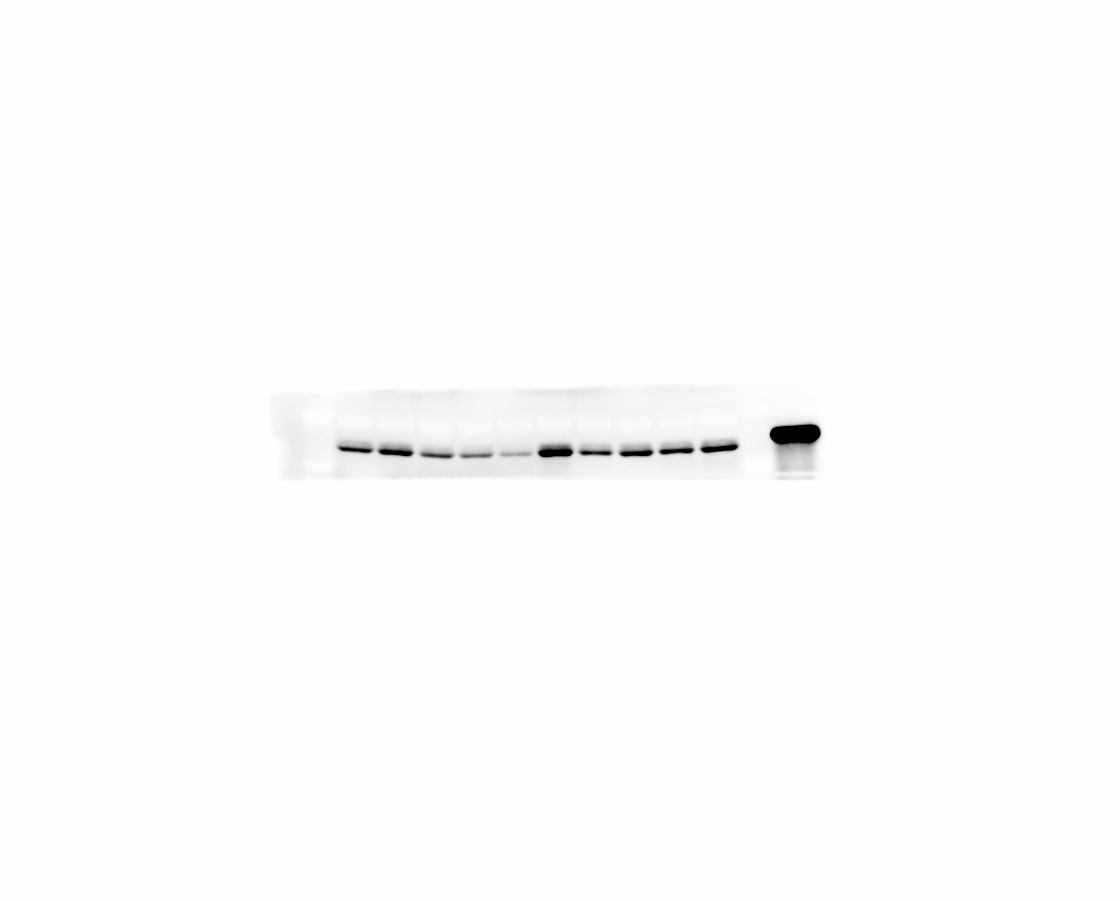
**

47kD

**
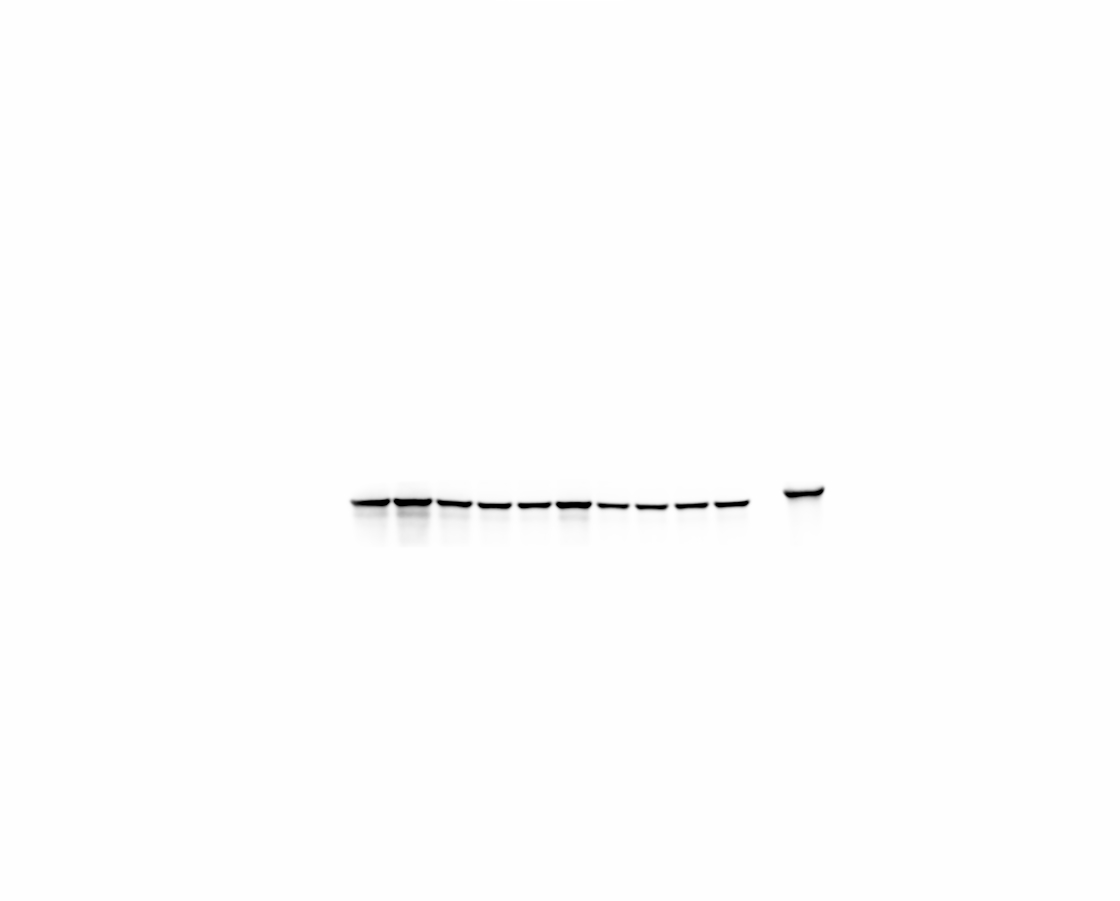
5.GFAP**

50kD

**6.Beta Actin**

**
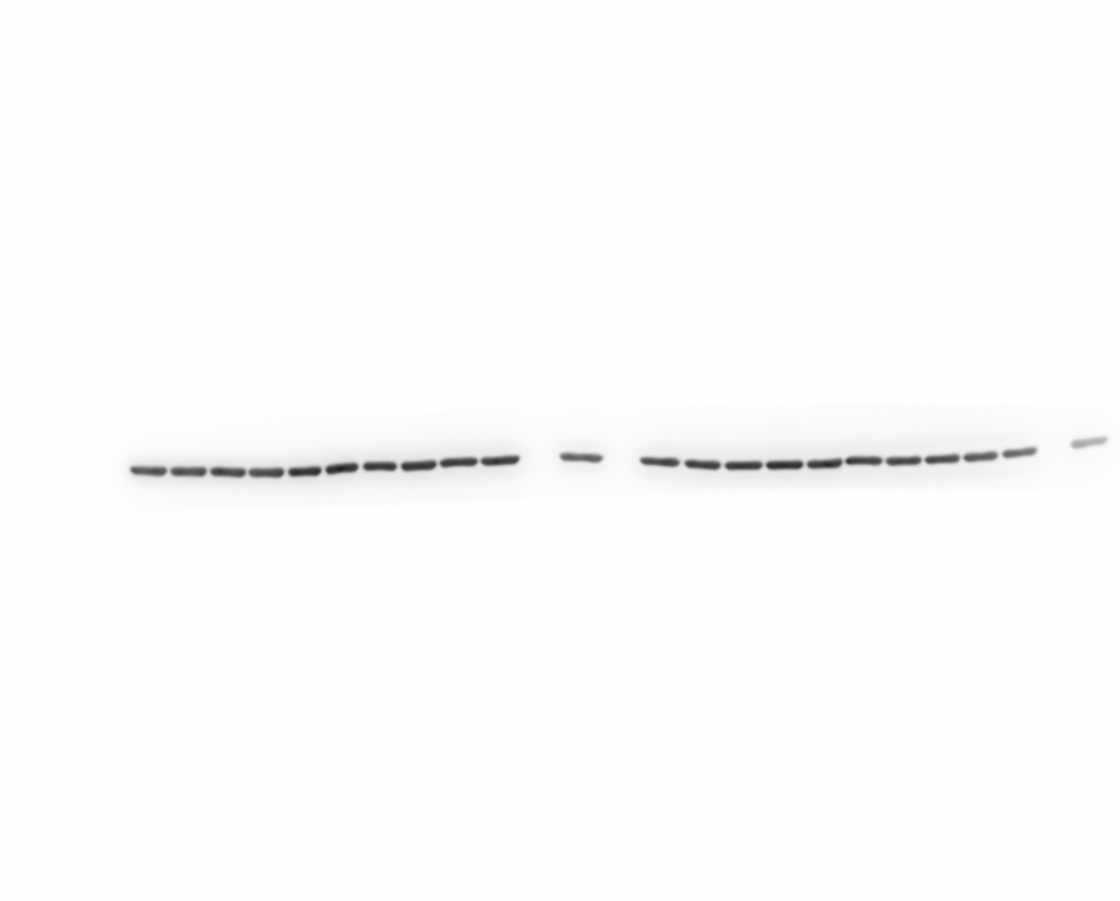
**

42kD

**Fig. 7 A**

1. **HMOX1**

**
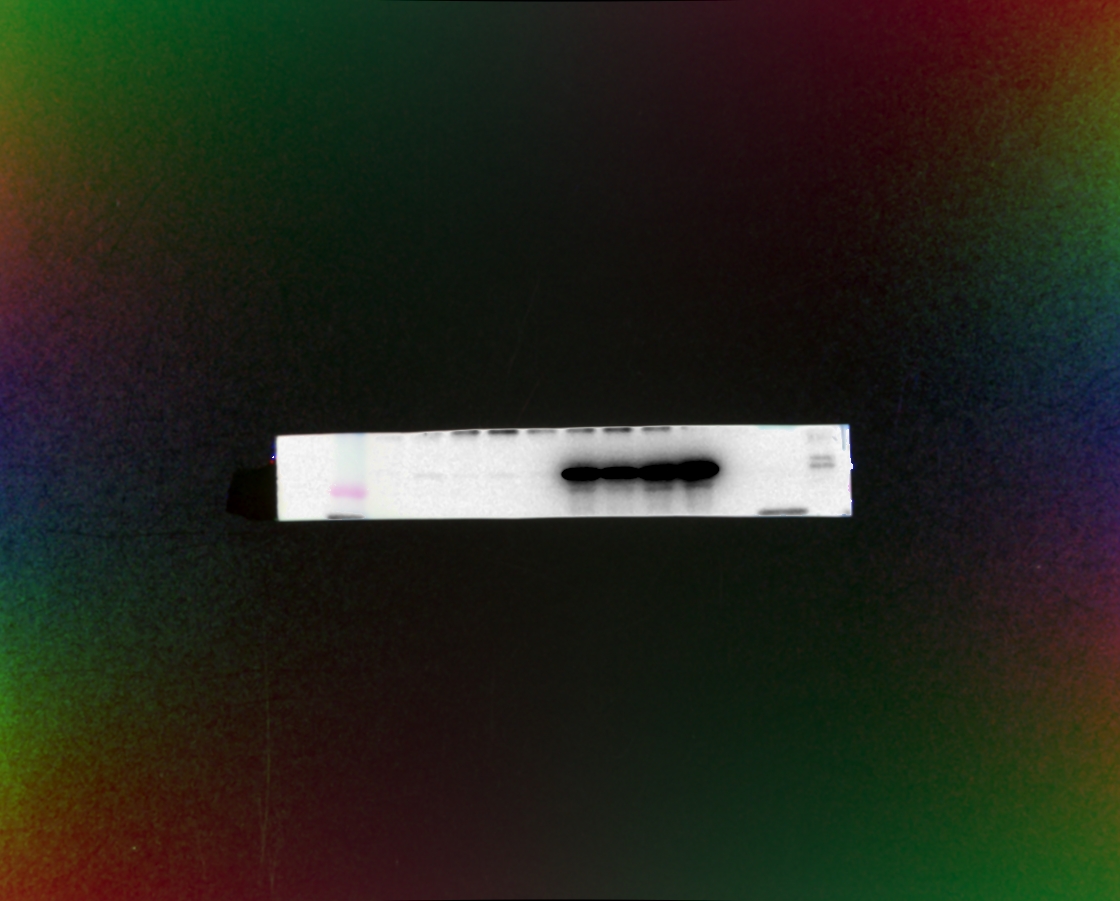
**

32kD

1.
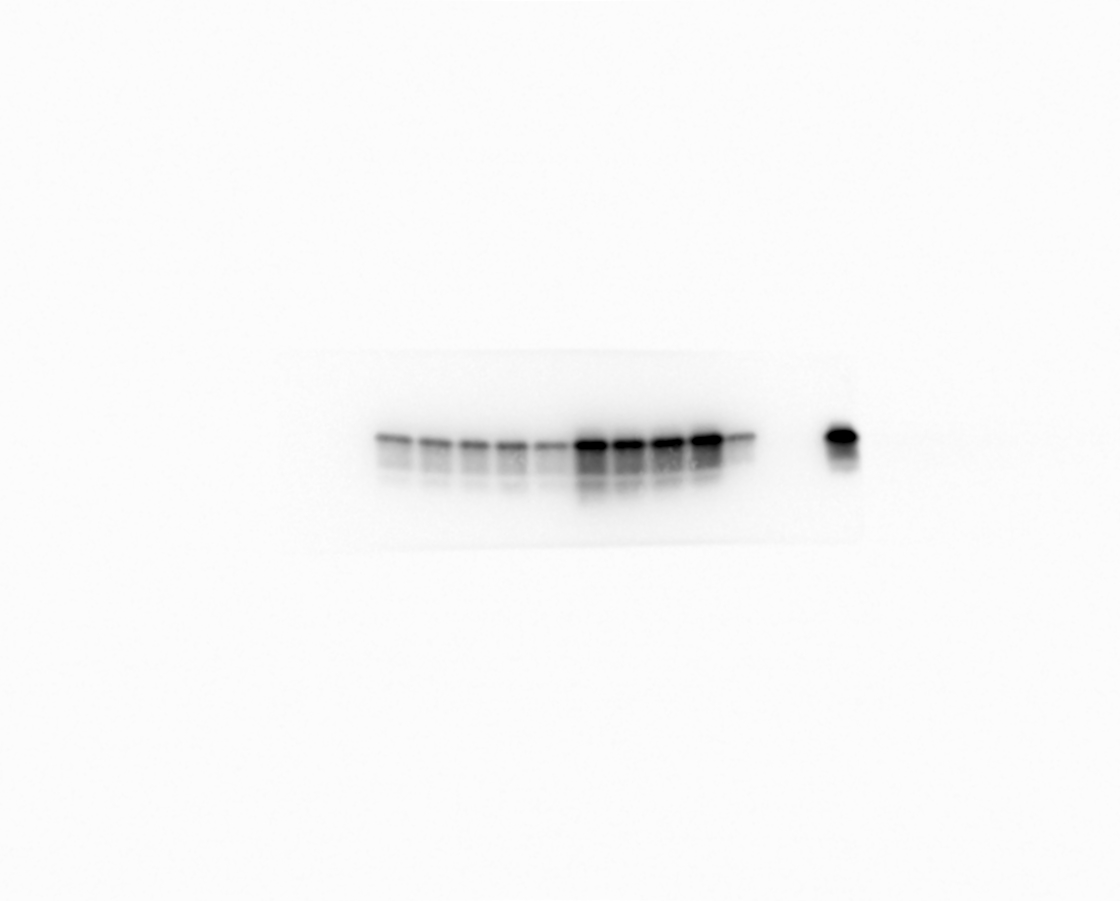
**FTH1**

21kD

**3.FTL**

**
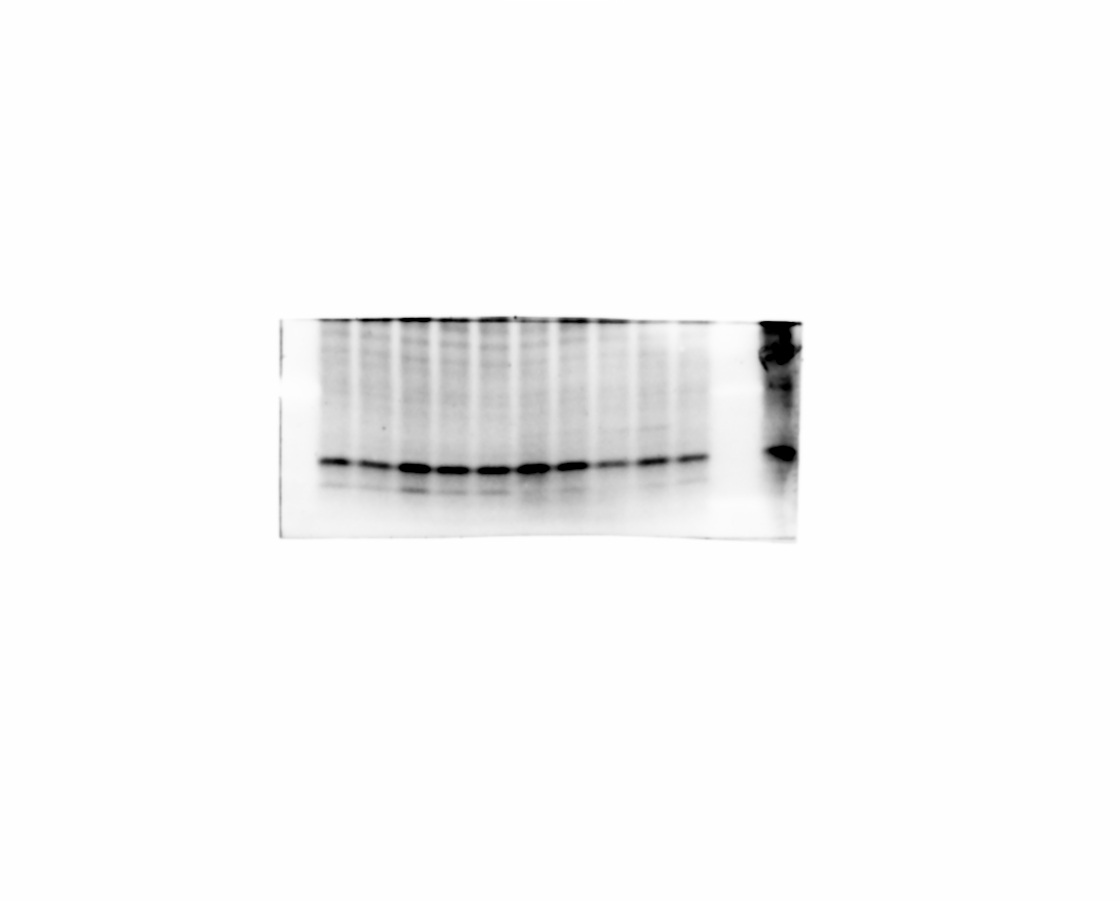
**

20kD

1. **FSP1 (40KD)**

**5 . Beta Actin**

**
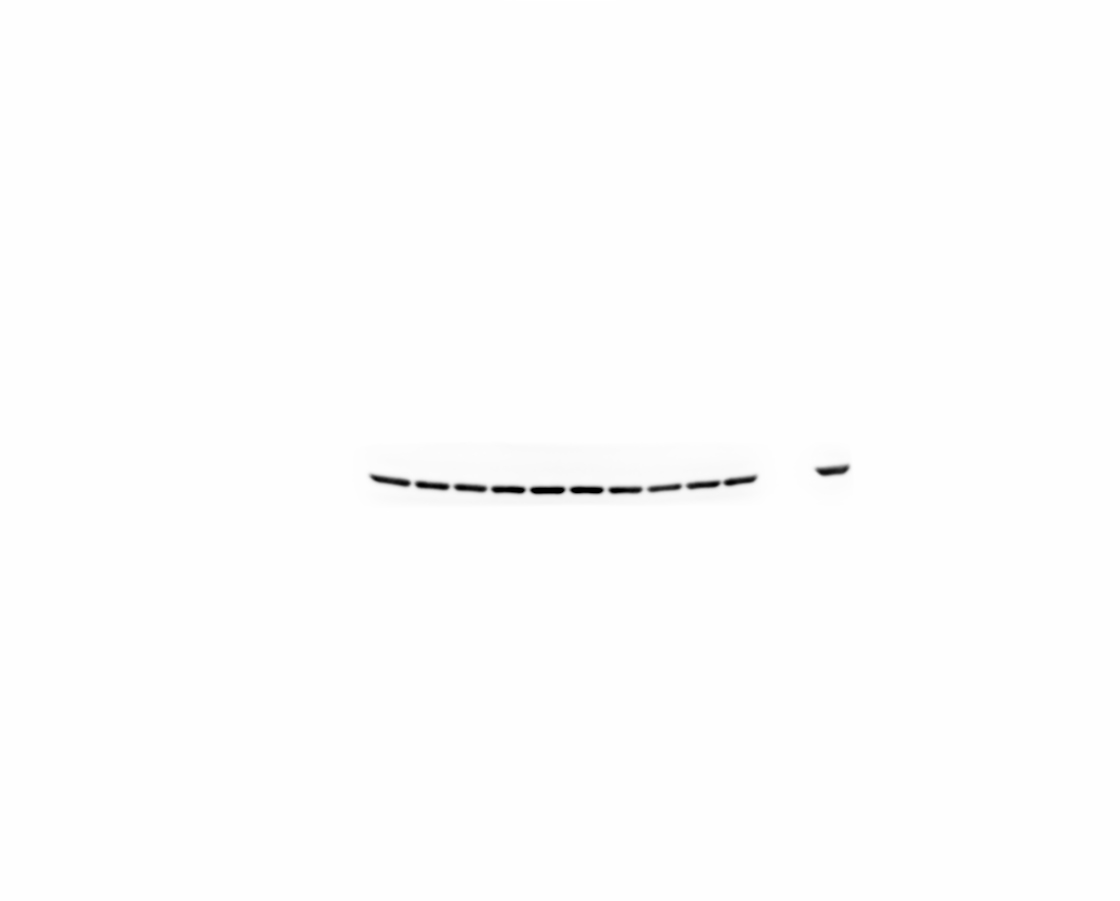
**

42kD

**Fig. 7 B**

**
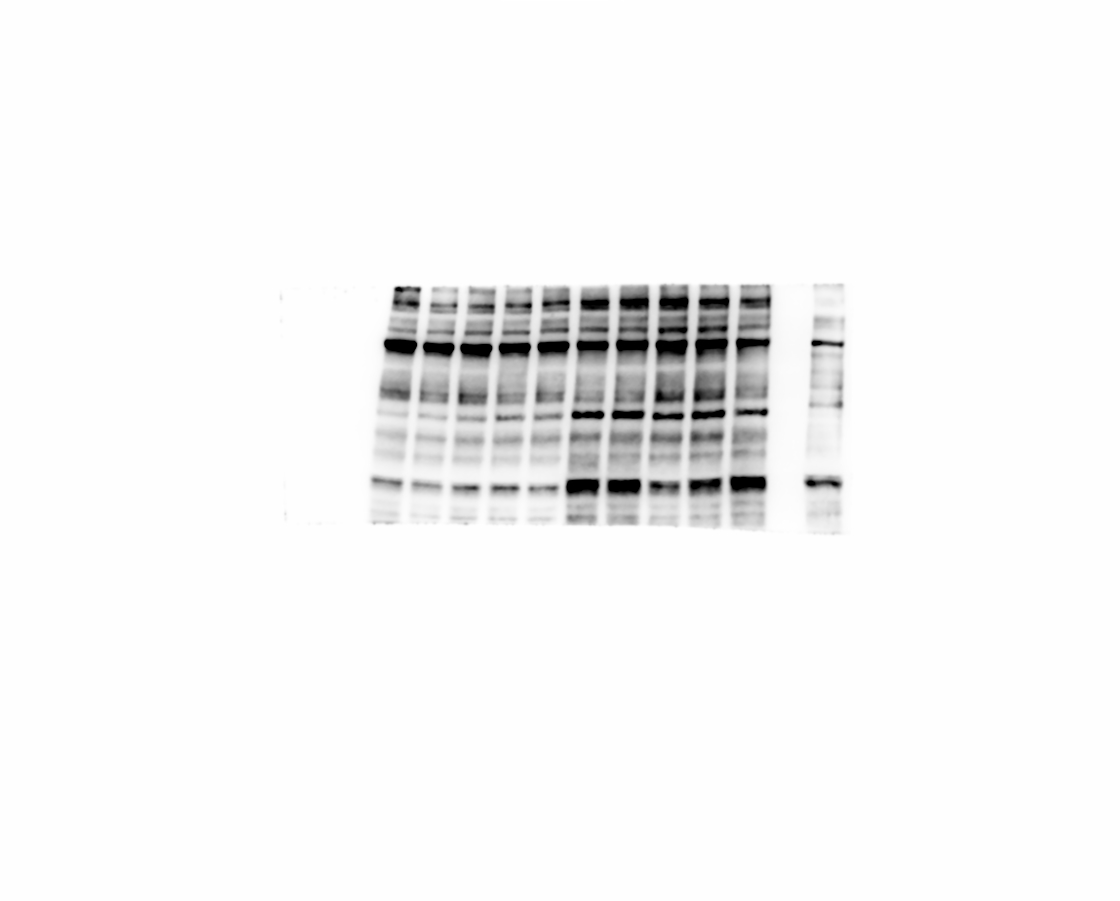
LPCAT3**

52kD

**
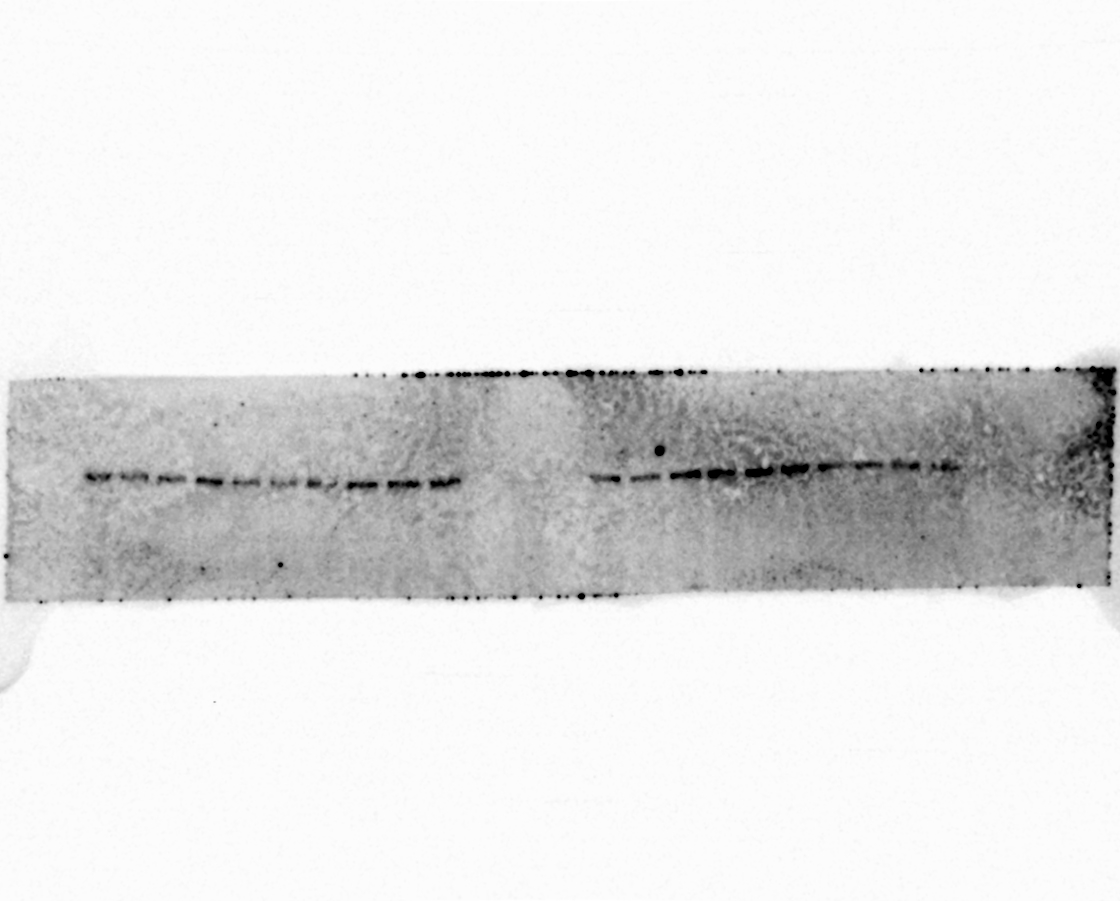
2.cPLA2**

110kD

1. **
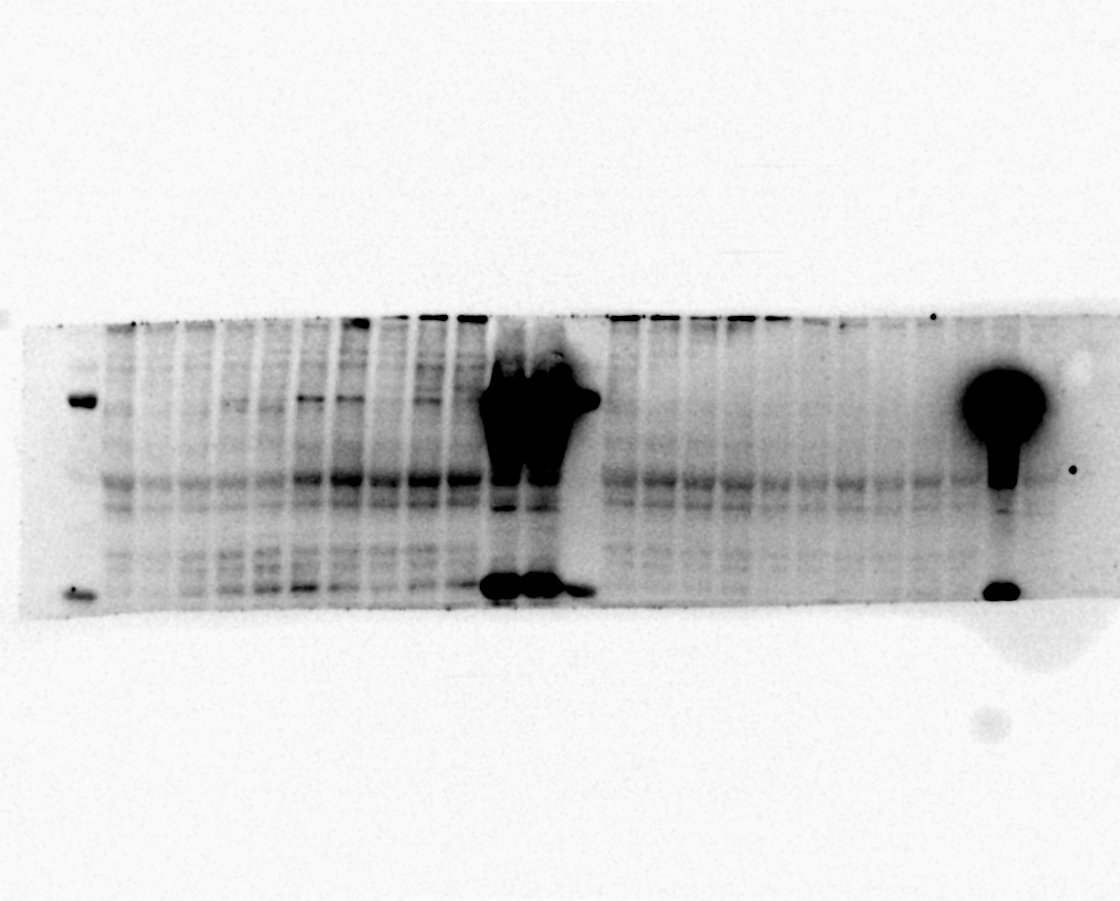
DGAT1**

55kD

**
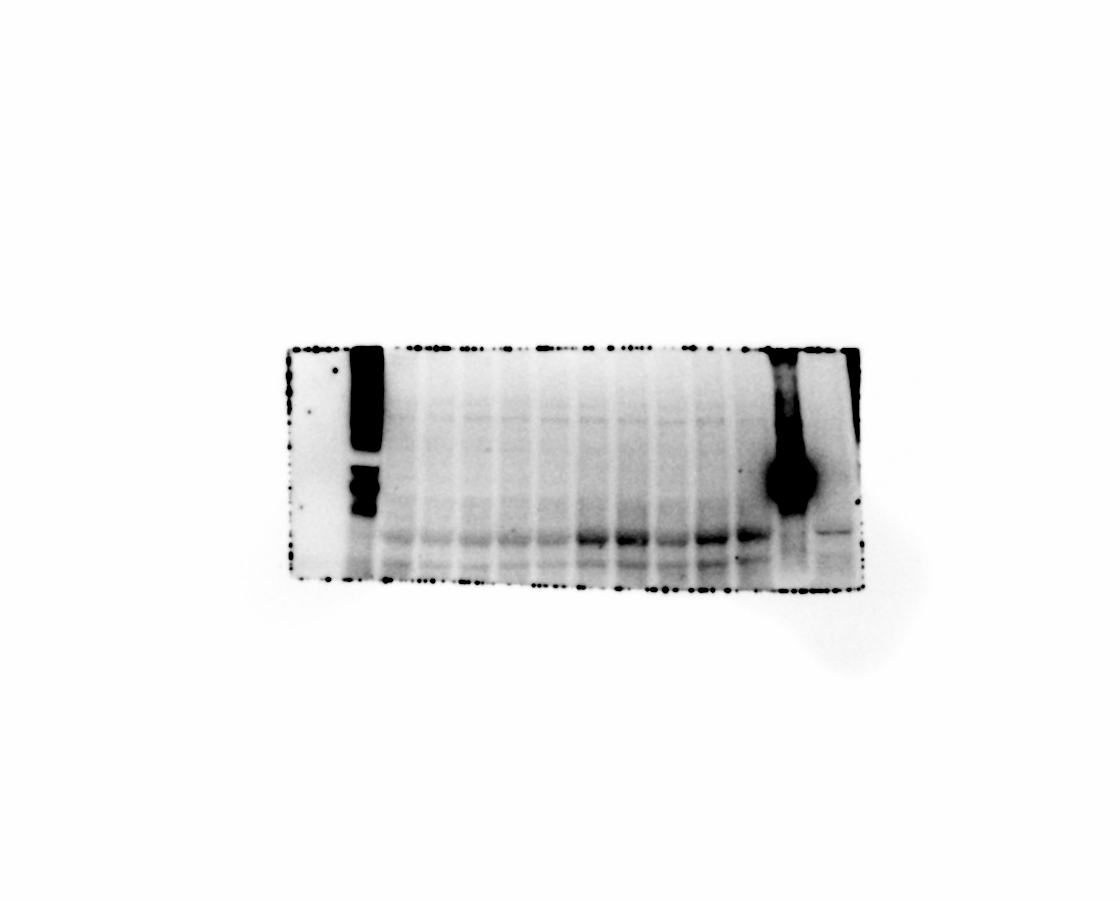
4.PLA2G7**

49kD

**
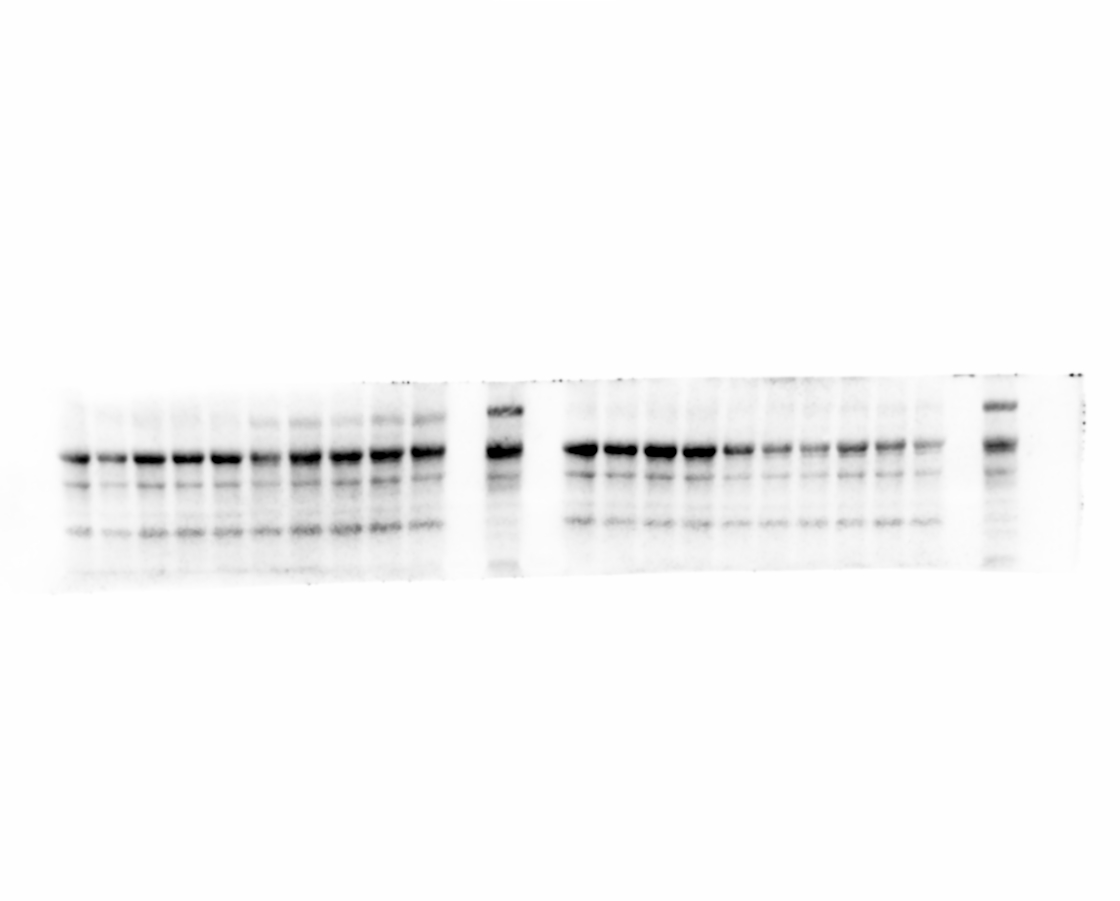
5.DGAT2**

40kD

**
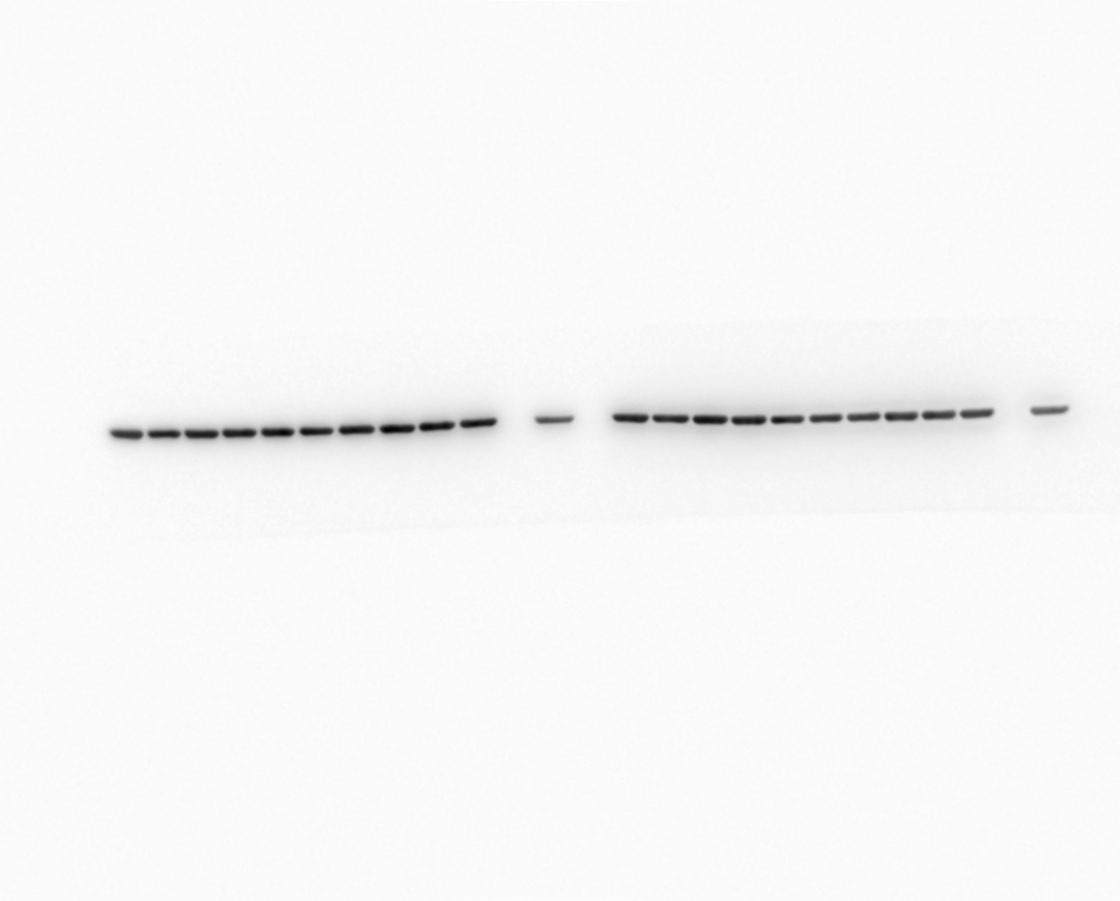
6.Beta Actin**

42kD

**
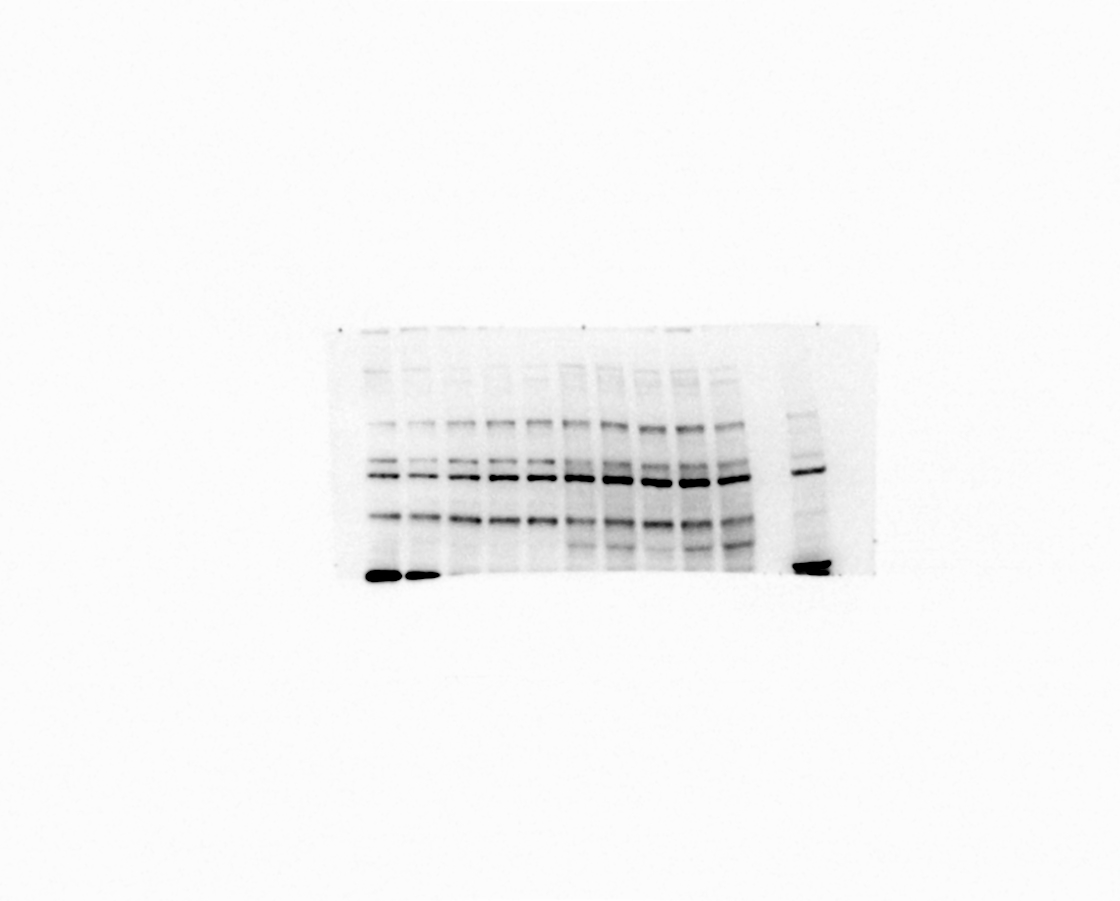
Fig. 7C**

1. **5-LOX**

78kD

**
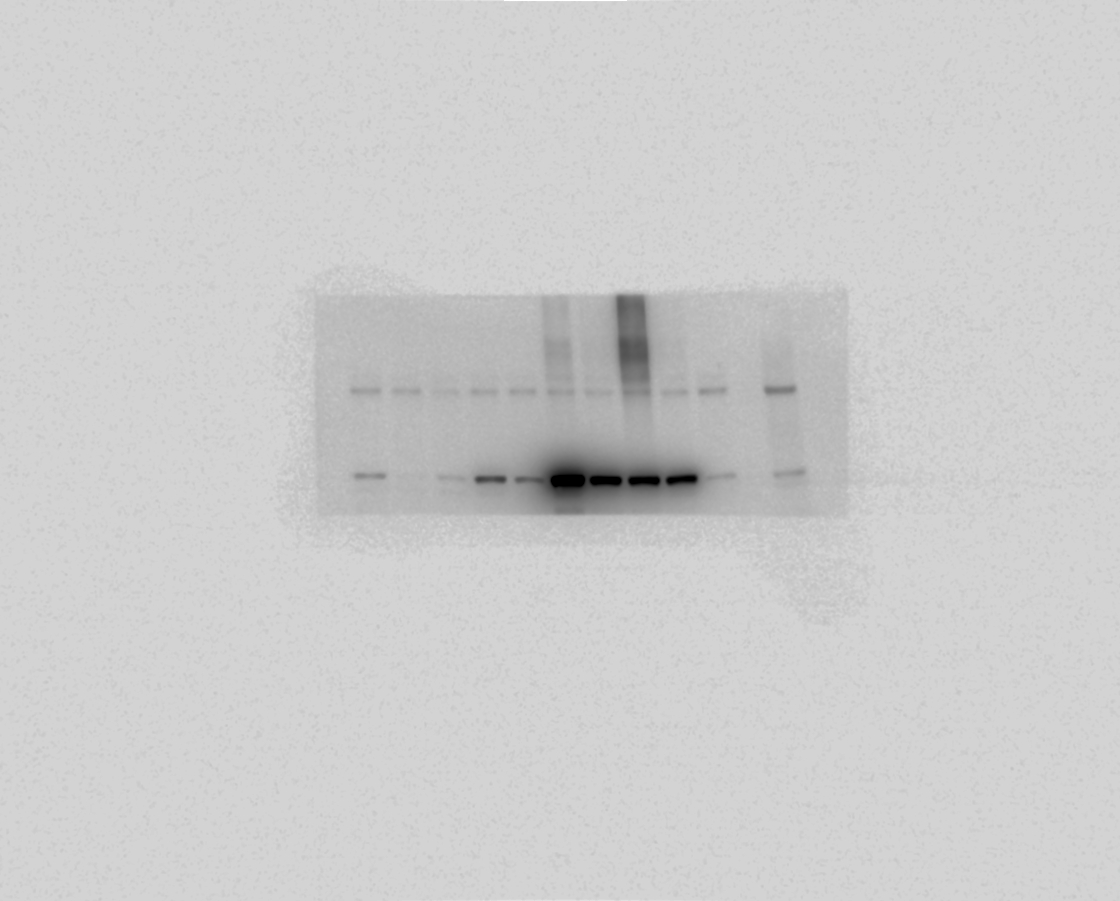
2. 15-LOX**

75kD

**3. 12-LOX**

**
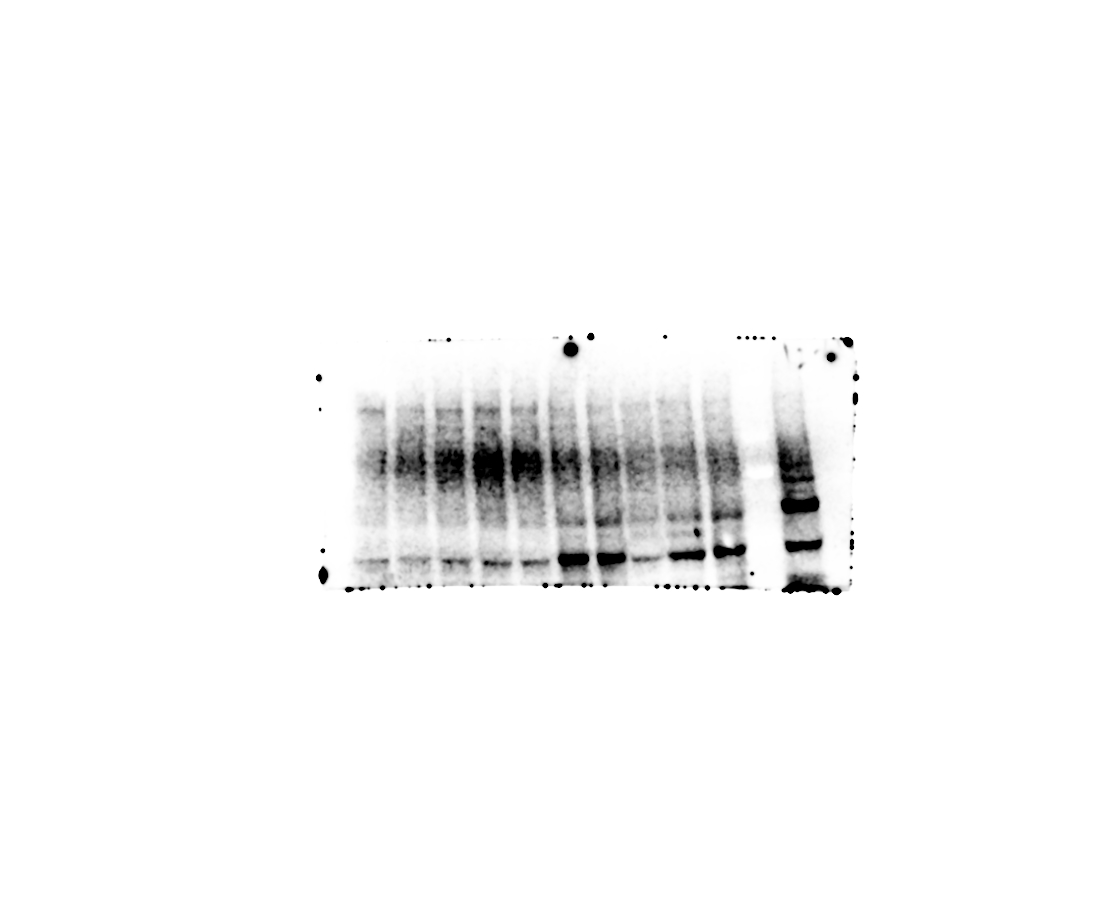
**

75kD

**4.COX2**

**
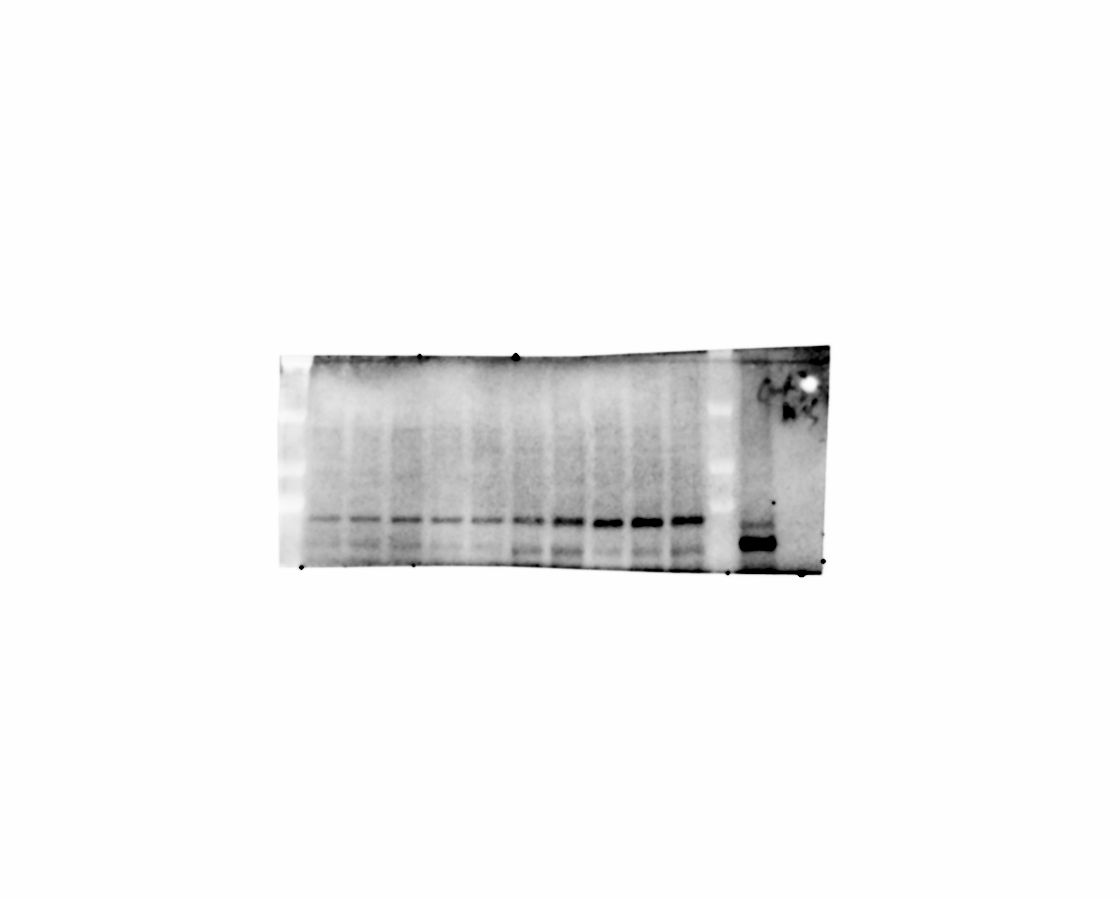
**

72kD

**5.CYP4A**

**
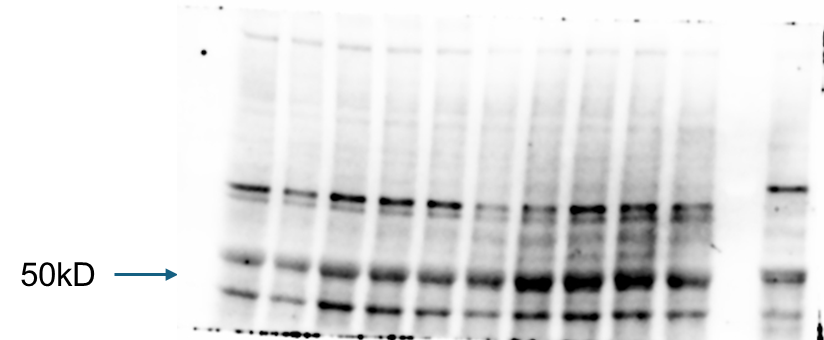
**

**6.sEH**

**
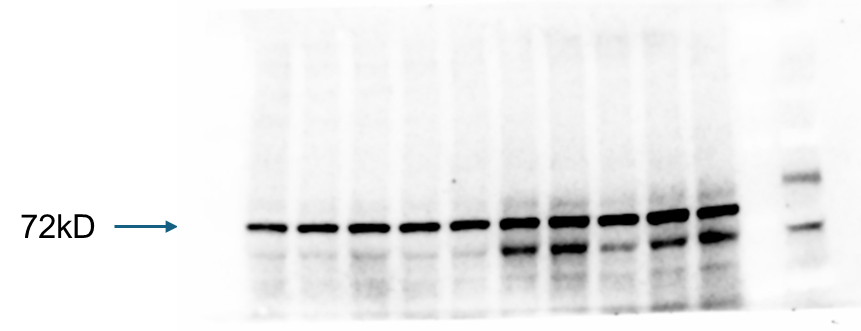
**

**7.Beta Actin**

**
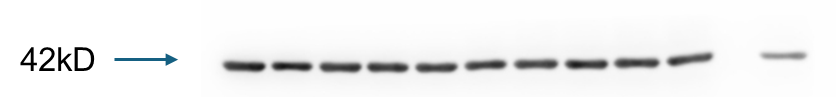
**

**Fig. 7D**

**1.HMOX1**

**
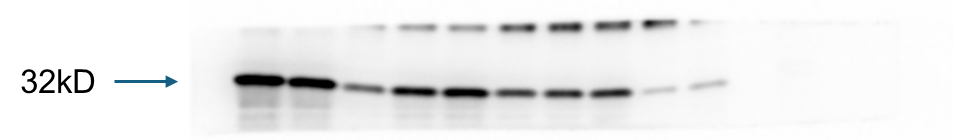
2.FTH1**

**
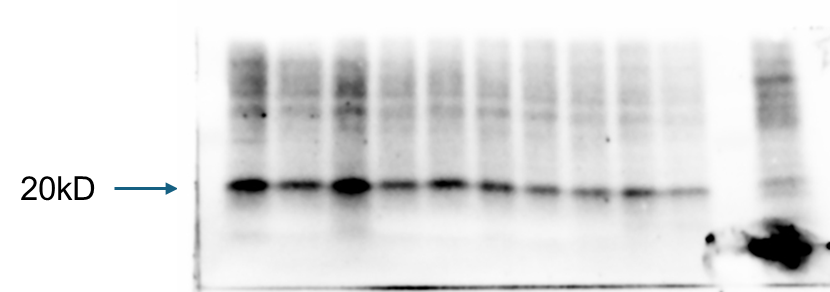

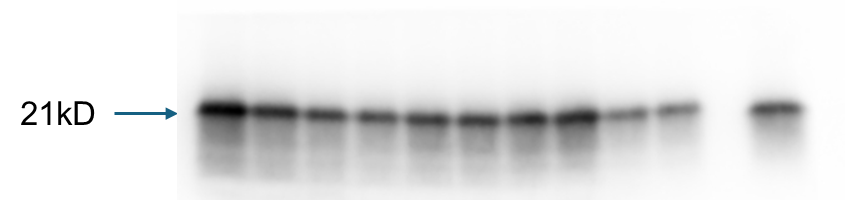
3.FTL**

**
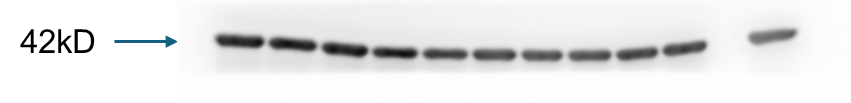
4. Beta Actin**

1. **Fsp1 (40 KD)**

**
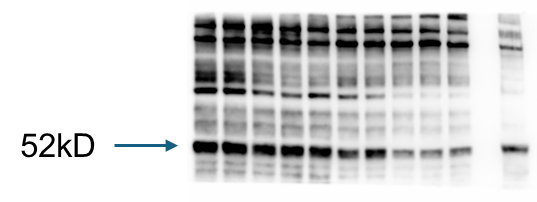
Fig. 7E**

**1.LPCAT3**

**2. cPLA2**

**
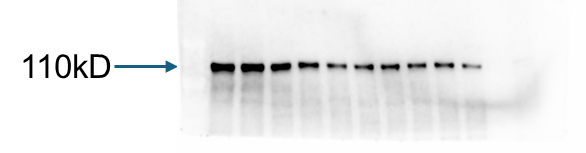
**

**
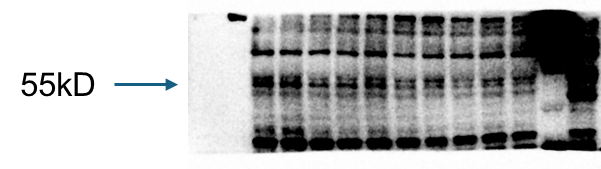
3.DGAT1**

**
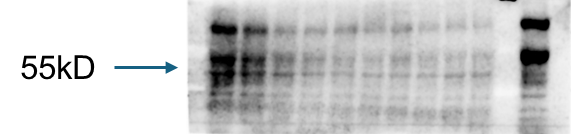
4.DGAT2**

**
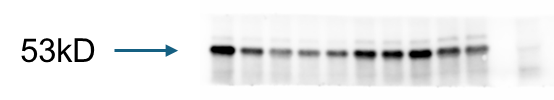
5.P53**

**
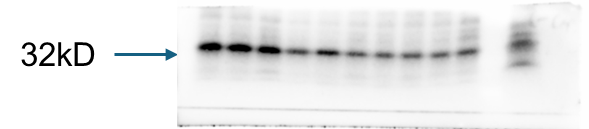
6.caspase3-**

**7.Beta Actin**

**
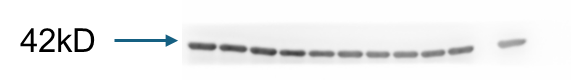
**

**Fig. 7F**

**5LOX**

**
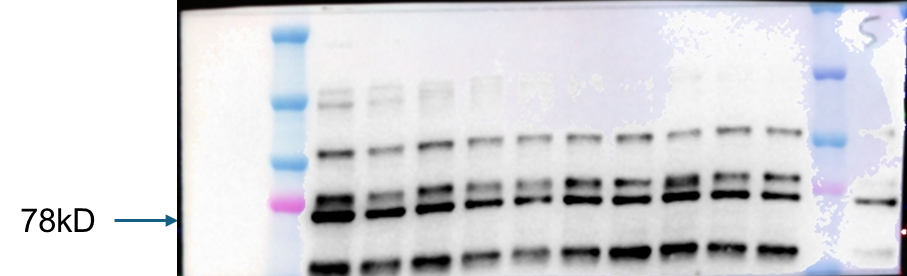
**

**2.12LOX-**

**
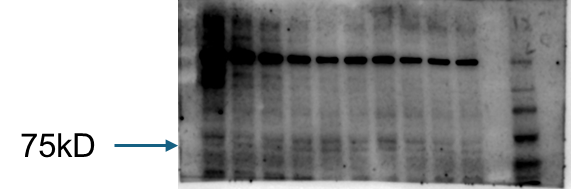
**

**3.15LOX**

**
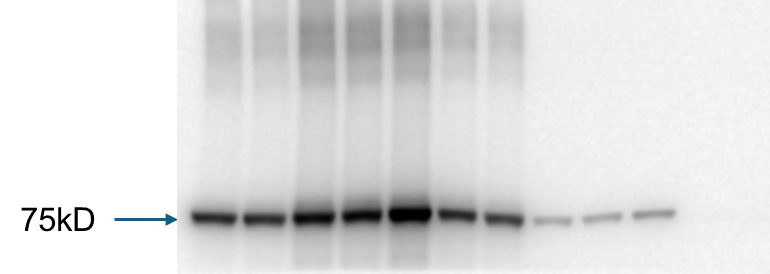
**

**4.sEH**

**
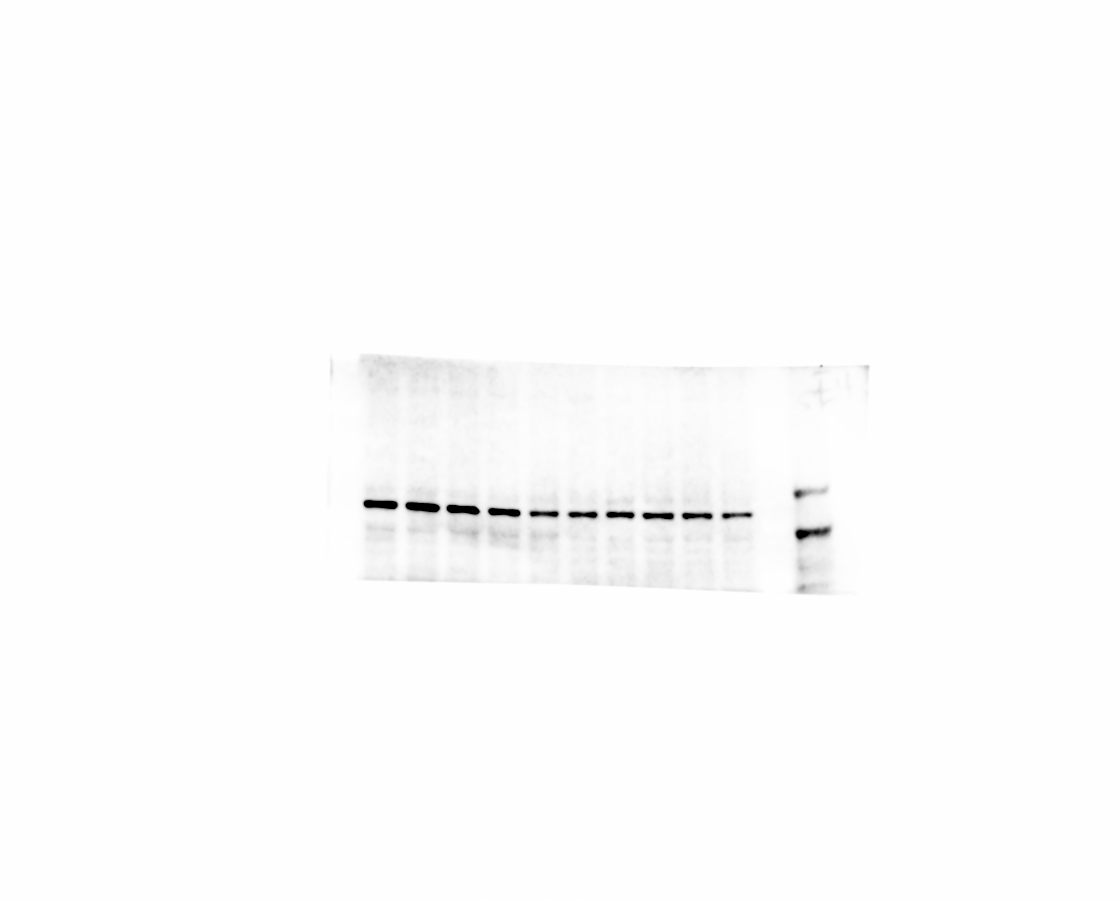
**

72kD

**5.COX2**

**
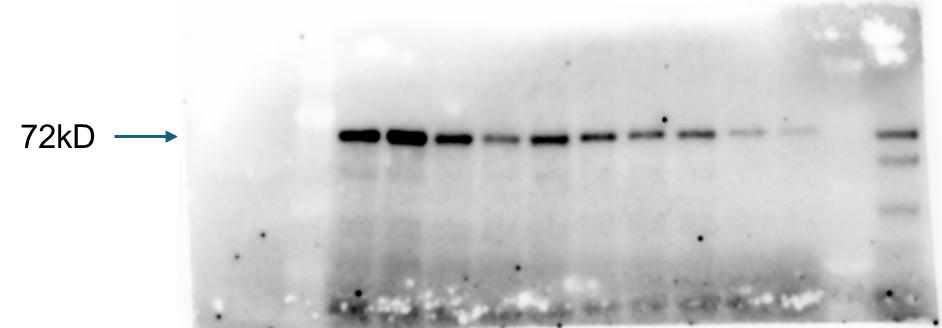
**

**6.CYP4A**

**
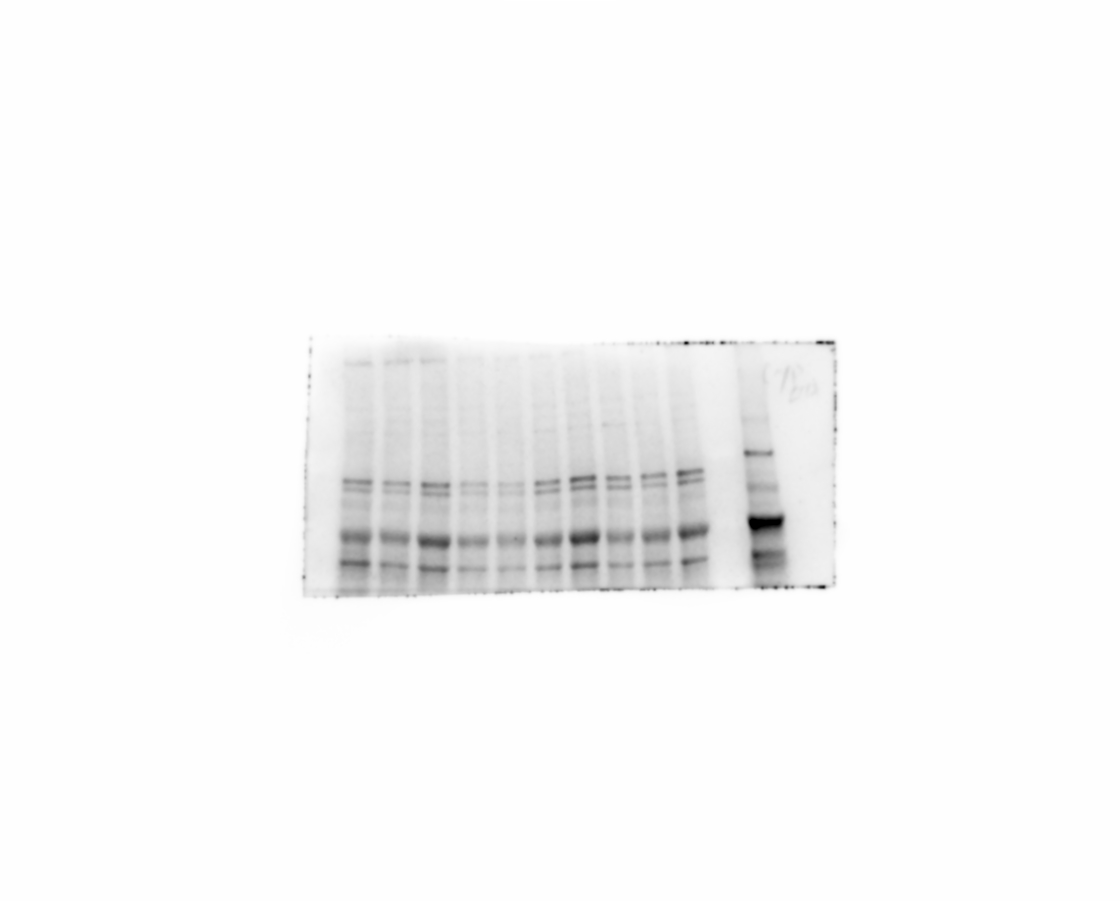
**

50kD

**7.Beta Actin**

**
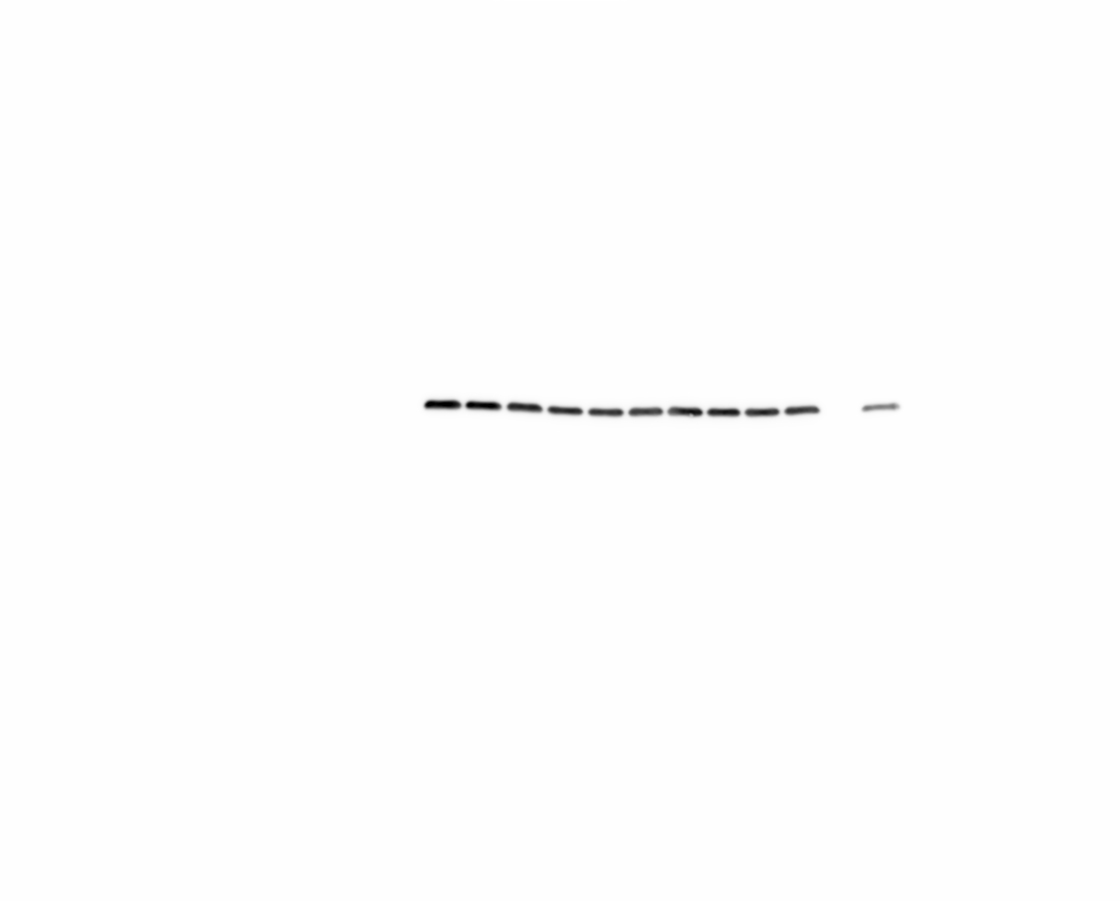
**

42kD

**
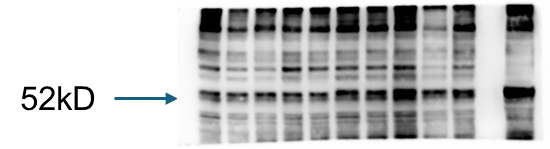
Fig 8.A Human germinal matrix (upper panel)**

**LPCAT3**

**2. 15LOX**

**
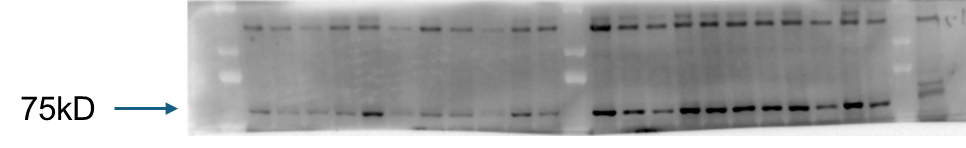
**

**3. COX2**

**
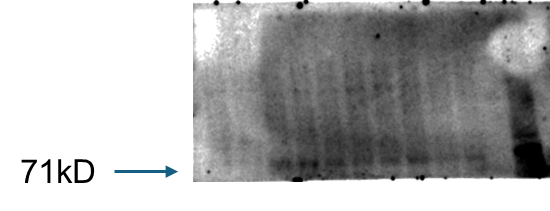
**

**
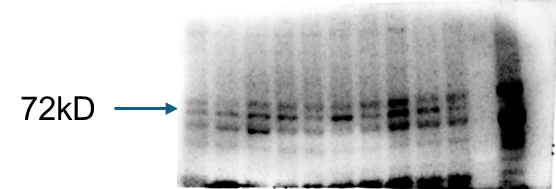
4.sEH**

**
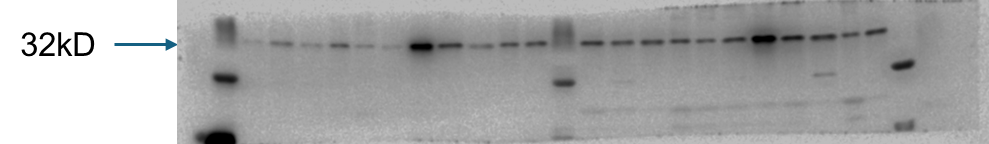
5.HMOX1**

**6.FTH1**

**
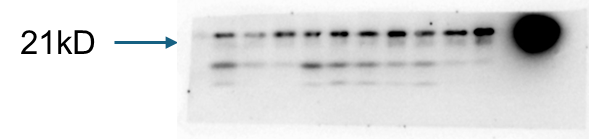
**

**7.P53**

**
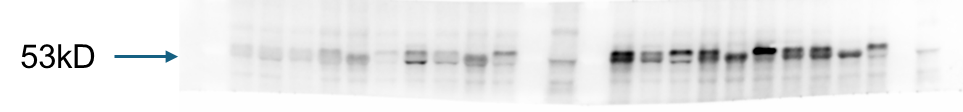
**

**8.Beta Actin**

**
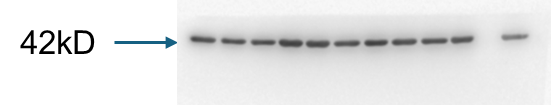
**

**Human White Matter (lower panel)**

1. **LPCAT3**

**
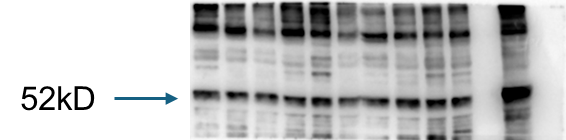
**

1. **15-LOX**

**
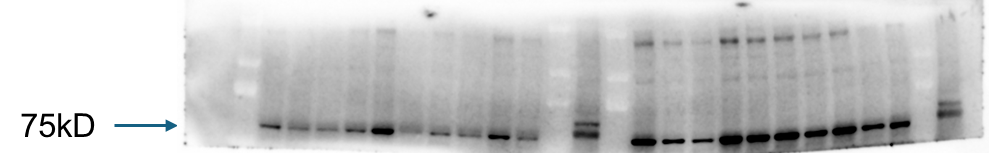
**

**75kd**

**3. COX2**

**
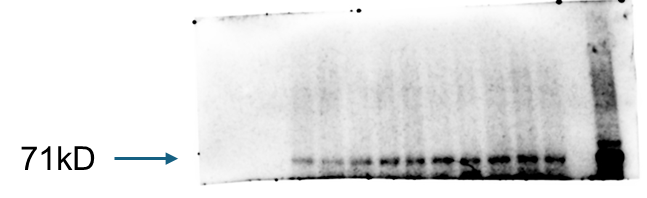
**

**
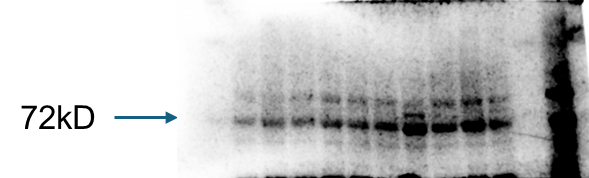
4. sEH**

**5. HMOX1**

**
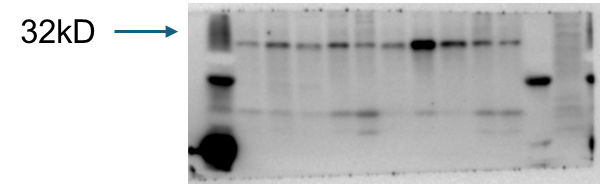
**

**
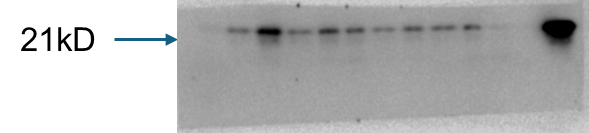
6. FTH1**

**
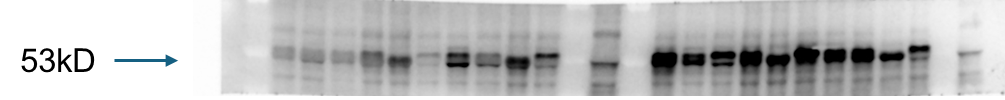
7.P-53 53kd**

**8. Beta Actin**

**
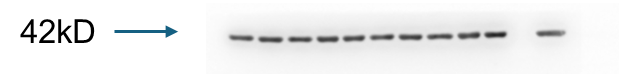
**
